# Evaluation of the impact of Illumina error correction tools on de novo genome assembly

**DOI:** 10.1186/s12859-017-1784-8

**Published:** 2017-08-18

**Authors:** Mahdi Heydari, Giles Miclotte, Piet Demeester, Yves Van de Peer, Jan Fostier

**Affiliations:** 1 Department of Information Technology, Ghent University-imec, IDLab, Ghent, B-9052 Belgium; 2 Center for Plant Systems Biology, VIB, Ghent, B-9052 Belgium; 3 Department of Plant Biotechnology and Bioinformatics, Ghent University, Ghent, B-9052 Belgium; 4 Bioinformatics Institute Ghent, Ghent, B-9052 Belgium; 5 Department of Genetics, Genome Research Institute, University of Pretoria, Pretoria, South Africa

**Keywords:** Next-generation sequencing, Error correction, Illumina, Genome assembly

## Abstract

**Background:**

Recently, many standalone applications have been proposed to correct sequencing errors in Illumina data. The key idea is that downstream analysis tools such as *de novo* genome assemblers benefit from a reduced error rate in the input data. Surprisingly, a systematic validation of this assumption using state-of-the-art assembly methods is lacking, even for recently published methods.

**Results:**

For twelve recent Illumina error correction tools (EC tools) we evaluated both their ability to correct sequencing errors and their ability to improve *de novo* genome assembly in terms of contig size and accuracy.

**Conclusions:**

We confirm that most EC tools reduce the number of errors in sequencing data without introducing many new errors. However, we found that many EC tools suffer from poor performance in certain sequence contexts such as regions with low coverage or regions that contain short repeated or low-complexity sequences. Reads overlapping such regions are often ill-corrected in an inconsistent manner, leading to breakpoints in the resulting assemblies that are not present in assemblies obtained from uncorrected data. Resolving this systematic flaw in future EC tools could greatly improve the applicability of such tools.

**Electronic supplementary material:**

The online version of this article (doi:10.1186/s12859-017-1784-8) contains supplementary material, which is available to authorized users.

## Background

Modern Illumina systems generate sequencing data with very high throughput and low financial cost. Illumina estimates that over 90% of sequencing data worldwide are generated on Illumina platforms. This data is characterized by a relatively short read length (100–300 bp) and a high accuracy (1–2% errors, mostly substitutions) [1]. Data generated on Illumina platforms suffers from various sources of bias, most notably a higher number of sequencing errors towards the 3’-end of the reads and a non-uniform distribution of reads across the genome [2].

Despite its short read length, Illumina data is often used for *de novo* genome assembly, sometimes complemented by data generated through other platforms. Most short-read assemblers first generate a de Bruijn graph from the input reads [3]. This graph represents all *k*-mers that occur in the input reads and the overlap between them. As such, de Bruijn graphs are used to efficiently establish the overlap between individual reads. The original genomic sequence is then represented as some path through the de Bruijn graph.

The presence of sequencing errors significantly complicates this task: a single sequencing error in a read results in up to *k* erroneous *k*-mers in the de Bruijn graph. These *k*-mers create artifacts in the de Bruijn graph such as spurious dead ends, parallel paths and chimeric connections [4]. Despite the low error rate, erroneous *k*-mers can vastly outnumber true *k*-mers, challenging the identification of the original sequence. To reduce the number of erroneous *k*-mers, trimming tools can be used as a primary solution to discard parts of each input read that have a per-base quality score below a user-defined threshold. However, this further reduces the read length and might aggravate the coverage bias.

Error correction tools (EC tools) on the other hand, try to identify and correct the sequencing errors. Often, this is achieved by generating a *k*-mer coverage spectrum from the input data and replacing poorly covered (and hence likely erroneous) *k*-mers by similar *k*-mers with a higher coverage. Sometimes, this process is further guided by using the per-base quality scores. Many standalone read error correction algorithms and implementations have been proposed for Illumina data, including ACE [5], BayesHammer [6], BFC [7], BLESS [8], BLESS 2 [9], Blue [10], EC [11], Fiona [12], Karect [13], Lighter [14], Musket [15], Pollux [16], Quake [17], QuorUM [18], RACER [19], SGA-EC [20] and Trowel [21]. For a comprehensive overview of the characteristics of these EC tools and those for other sequencing platforms, we refer to [22].

The key idea is that the prior application of EC tools on raw Illumina sequencing data provides assembly methods with cleaner input data and hence improves the quality of assembly both in terms of reduced fragmentation (i.e., longer contigs or scaffolds) and higher accuracy of the resulting assemblies. As a secondary goal, the prior use of EC tools may reduce the memory usage and the runtime of the assembly tool. This is useful when assembling larger genomes, a task that is typically quite resource-intensive.

Surprisingly, most EC tools are not evaluated on their ability to improve the quality of *de novo* genome assembly with modern assemblers, but rather directly on their ability to correct sequencing errors. Using simulated Illumina data, such an evaluation is straightforward as error-free data is known. In that case, the *error correction gain*, a metric that expresses to what degree the error rate is reduced, is used to describe the performance of EC tools. With real Illumina data, the error correction performance is typically assessed through the use of a read mapper: both corrected and uncorrected reads are aligned to their corresponding reference genome and various performance metrics are derived to express the reduction in mismatches in the respective alignments. EC tools that result in more aligned reads and/or alignments with fewer mismatches are assumed to be superior.

We argue that a lower average error-rate in the input data does not necessarily lead to better assembly results. First, the vast majority of sequencing errors are benign to the assembly process. For example, consider a sequencing error that gives rise to one or more erroneous *k*-mers that otherwise do not exist in the sequenced genome. In the de Bruijn graph, such sequencing error causes a spurious dead end or a short parallel path. These graph artifacts are easily detected and corrected for by many assembly tools assuming the corresponding true *k*-mers occur with sufficient coverage in the input reads. Only a relatively small fraction of sequencing errors is truly problematic, for example when they give rise to erroneous *k*-mers that do exist elsewhere in the genome. These errors thus give rise to spurious ‘chimeric’ connections between nodes in the de Bruijn graph that are otherwise distantly located in the original sequence. As such, they may result in misassemblies and/or shorter contig sizes. A second class of problematic errors are those that occur in regions with very low coverage. Such errors may render the assembly tool unable to detect overlap between reads because no *k*-mers are shared. Overall, an EC tool that is able to correct all benign sequencing errors and not a single problematic sequencing error might exhibit a high error correction gain but will not substantially improve the assembly process. Second, EC tools might introduce new errors in the sequence data. If such events are rare and unbiased, they may not pose a great threat to the assembly process. However, if EC tools systematically make the same mistake in a given context, the genome assembler may not be able to recover from this error.

Most state-of-the-art genome assembly tools have built-in algorithms to detect and handle sequencing errors, either directly or implicitly through a correction procedure on the de Bruijn graph. The prior use of standalone EC tools thus only makes sense if they outperform these built-in error correction algorithms. Table 1 lists for every EC tool the accuracy analyses that were performed in the accompanying publication. Even though all tools were evaluated for their ability to reduce sequencing errors, their ability to improve the genome assembly process is either lacking or performed with older assembly tools. Also, recent review papers on EC tools [23, 24] did not contain such analyses.

**Table 1:** List of EC tools evaluated in this paper

EC tool	Algorithm	Data structure	Indel support	Accuracy analysis	Assembly analysis	Year
ACE	*k*-mer	*k*-mer trie		Read level	-	2015
BayesHammer	*k*-mer	Hamming graph		Read level	SPAdes	2013
BFC	*k*-mer	Bloom filter		Read level	Velvet, ABySS [34]	2015
BLESS 2	*k*-mer	Bloom filter		Read level	Gossamer [35]	2016
Blue	*k*-mer	Hash table	$\checkmark $	Read level	Velvet	2014
Fiona	MSA	Suffix tree	$\checkmark $	Base level	-	2014
Karect	MSA	Partially-ordered graph	$\checkmark $	Read, base level	Velvet, SGA, Celera [36]	2015
Lighter	*k*-mer	Bloom filter		Read level	Velvet	2013
Musket	*k*-mer	Bloom filter		Base level	SGA	2013
RACER	*k*-mer	Hash table		Read level	-	2013
SGA-EC	MSA	Suffix array		Read level	SGA	2012
Trowel	*k*-mer	Hash table		Read, base level	Velvet, SOAPdenovo [37]	2014

The algorithmic approach is either *k*-mer spectrum based (‘*k*-mer’) or multiple sequence alignment based (‘MSA’). Tools can be further classified according to data structure and heuristics used. Some tools are able to correct insertions or deletions. In their accompanying publication, all tools were assessed directly on their ability to reduce error rate, either on the read or base level. Most tools did not use assembly analyses with modern assemblers in their evaluation. SPAdes was used for the evaluation of BayesHammer, but no comparison was made with assembly results from uncorrected data

In this paper, we review twelve recently published EC tools. We compiled a benchmark suite of eight public datasets sequenced from organisms with a genome size ranging from 2 to 116 Mbp and assessed the performance of the different EC tools both on their potential to correct the sequencing errors and on their ability to improve assembly results using four assemblers (DISCOVAR [25], IDBA [26], SPAdes [27] and Velvet [4]). We discuss the impact on the resulting assembly quality and investigate systematic errors in some of the EC tools. Finally, computational efficiency (memory usage and runtime) of the different EC tools is discussed. Note that the effect of error correction for other applications such as variant calling is beyond the scope of this paper.

## Methods

### Error correction tools

Twelve state-of-the-art (published in 2012 or later) EC tools for Illumina data were included in this review and listed in Table 1. We were unable to produce corrected reads with QuorUM and EC and hence these tools were excluded in this study.

EC tools have been classified according to their underlying algorithmic principles in several review papers [22, 23, 28]. In Table 1, tools were classified according to their main algorithmic approach: *k*-mer spectrum based or multiple sequence alignment (MSA) based. The *k*-mer spectrum based tools operate on the level of individual *k*-mers. First, the complete set of *k*-mers that occur in the input data and their corresponding frequency is determined. Second, reads that contain rarely occurring *k*-mers are assumed to contain sequencing errors and are modified, using a minimum edit distance strategy, such that these *k*-mers are replaced by similar, more frequently occurring *k*-mers. In contrast, MSA-based tools operate on the level of reads. First, reads that are assumed to represent overlapping genomic regions are clustered together and a consensus is obtained through multiple alignment. Second, reads are corrected according to the consensus alignment. While all EC tools considered in this review rely on either of these two approaches, there is still a great diversity in the specific implementation heuristics and data structures (bloom filter, hash table, suffix tree, …).

Most tools require users to specify a *k*-mer length to be used during the error correction procedure. The optimal value can differ from one dataset to another, depending on the coverage, genome size and error distribution. This optimal value was empirically obtained by running the EC tool multiple times with different *k*-mer sizes and selecting the *k*-mer size that yields the most contiguous SPAdes assembly results as measured in terms of N50. This optimal value was used to produce the results of Table 4. For all other tables and figures, the default or recommended *k*-mer size was used for all datasets. Parameters and settings are provided in Additional file 1: Section 1. All tools support multithreading, and with the exception of ACE and RACER, the number of parallel threads can be specified. Those tools were run with 32 threads. Runtime and peak memory usage were measured with the GNU ‘time -v’ command. We recorded elapsed (wall clock) time and peak resident memory usage. All tools were run on a machine with four Intel(R) Xeon(R) E5-2698 v3 @ 2.30 GHz CPUs (64 cores in total) and 256 GB of memory.

### Data

Tools are benchmarked on eight datasets for which both a high quality reference genome and real Illumina data are publicly available (see Table 2). Genome sizes range from 2 Mbp (*Bifidobacterium dentium*) to 116 Mbp (*Drosophila melanogaster*) while read coverage varies from 29 X to 612 X. Data is produced by the Illumina HiSeq, MiSeq and GAII platforms with read lengths varying between 100 bp and 251 bp. Two of the datasets have a variable read length due to read trimming, all other datasets have fixed read lengths.

**Table 2:** Real datasets used for the evaluation of EC tools

Abbr.	Organism	Reference ID	Genome size	Cov.	Sequencing platform	Read length	Trimmed reads	Dataset ID	Ref.
D1	*Bifidobacterium dentium*	Nc013714.1	2.6 Mbp	373 X	Illumina MiSeq	251 bp		SRR1151311	[23]
D2	*Escherichia coli K-12 DH10B*	NC010473	4.5 Mbp	418 X	Illumina MiSeq	150 bp		Ill. Data library	[10]
D3	*Escherichia coli K-12 MG1655*	NC000913	4.5 Mbp	612 X	Illumina GAII	100 bp		ERA000206	[10]
D4	*Salmonella enterica*	NC011083.1	4.7 Mbp	97 X	Illumina MiSeq	239 bp	$\checkmark $	SRR1206093	[23]
D5	*Pseudomonas aeruginosa*	ERR330008	6.1 Mbp	169 X	Illumina MiSeq	120 bp	$\checkmark $	ERR330008	[10]
D6	*Homo sapiens* Chr. 21	HG19	45.2 Mbp	29 X	Illumina HiSeq	100 bp		Ill. Data library	[10]
D7	*Caenorhabditis elegans*	WS222	97.6 Mbp	58 X	Illumina HiSeq	101 bp		SRR543736	[23]
D8	*Drosophila melanogaster*	Release 5	116.4 Mbp	52 X	Illumina HiSeq	100 bp		SRR823377	[23]

To assess the performance of tools on simulated data, synthetic Illumina reads for the same set of organisms were generated using ART [29]. The same coverage and read lengths were used as for the real data (Additional file 1: Section 2). ART also generates a corresponding set of error-free reads, which greatly facilitates the evaluation of EC tools on synthetic data.

### Error metrics

The error rate is the ratio of the total number of sequencing errors (substitutions or indels) and the number of nucleotides in the input data. Error correction performance is measured as follows: true positives (TP) correspond to corrected errors; true negatives (TN) correspond to initially correct bases left untouched; false positives (FP) correspond to newly introduced errors; false negatives (FN) correspond to unidentified errors. The error correction gain (EC gain) is defined as:

$$\text{EC gain}=\frac{\text{TP}-\text{FP}}{\text{TP}+\text{FN}}. $$

The EC gain measures the degree in which the error rate is reduced. A gain of 100% means all errors were corrected and no new errors were introduced. The sensitivity (true positive rate – TPR) is defined as follows:

$$\text{TPR} = \frac{\text{TP}}{\text{TP}+\text{FN}}. $$

### Evaluation of assembly results

To assess the impact of error correction on *de novo* assembly results, the following assemblers were used: DISCOVAR, IDBA, SPAdes and Velvet. All four assemblers have built-in error correction functionality. Velvet, IDBA and SPAdes remove erroneous *k*-mers through the identification of parallel paths (‘bubbles’ and ‘tips’) in the de Bruijn graph. SPAdes and IDBA iteratively increase the *k*-mer size. This way, they take advantage of shorter *k*-mers for a sensitive detection of overlap between reads and of longer *k*-mers for dealing with repeat resolution. DISCOVAR uses a different methodology: for each read, a group of ‘true friends’ is determined. These are reads that share a *k*-mer with the read and that do not have a high quality base difference with the read. DISCOVAR then corrects each read based on the consensus sequence obtained from the multiple sequence alignment of its true friends.

We investigated the underlying causes of suboptimal assembly results after error correction. MUMmer [30] was used to align contigs, and to check if the contig has no structural misassemblies. In order to determine the *k*-mer frequencies Jellyfish [31] was used.

## Results and discussion

### Ability of EC tools to correct sequencing errors

**Fig. 1:**
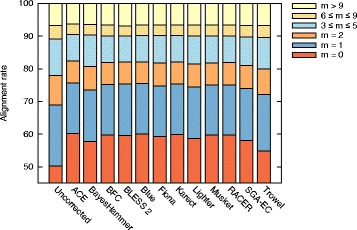
Mismatches in read alignment. Classification of (un)corrected reads for *D. melanogaster*, based on the number of mismatches in their alignment to the reference genome

In order to estimate the reduction in error rate through the use of EC tools, both uncorrected and corrected data were aligned to the corresponding reference genome using BWA [32]. For all datasets D1-D8 and EC tools, the fraction of reads that align with respectively *m*=0 and *m*>9 mismatches is reported in Additional file 1: Section 3.1. All EC tools are able to substantially reduce the number of mismatches required for read alignment. This is especially true for bacterial genomes, where often >95% of the corrected reads show perfect alignment with the reference. In contrast, for larger genomes, this is typically in the range of 60–80%. Error correction also reduces the fraction of highly erroneous reads (i.e., reads that require more than 9 mismatches to align), albeit to varying degrees. For the largest dataset D8 (*D. melanogaster*), Fig. 1 provides a more detailed breakdown of the number of mismatches *m* required for read alignment. Initially, about 50% of the uncorrected reads perfectly align. ACE shows the highest increase of this figure to 60.14%. ACE also has the lowest percentage of highly erroneous reads.

After applying error correction to a read, there is no guarantee that BWA will again align that read to the same genomic location. Therefore, this evaluation metric might favor overly aggressive EC tools that transform reads into similar reads that do exist in the genome, but that do not represent the actual sequenced genomic region. Therefore, in an alternative evaluation metric, we assume that the error-free read is represented by the segment of the reference genome to which the uncorrected read aligns. Uncorrected reads that can not be mapped to the reference genome are excluded from this evaluation. As BayesHammer and BLESS 2 do not provide a one-to-one correspondence between input and output, they are not included in this evaluation.

Table 3 shows the EC gain, the percentage of corrected errors and the number of newly introduced errors per Mbp of read data for each of the eight datasets. Detailed confusion matrices are provided in Additional file 1: Section 3.2.2. Major differences in EC gain can now be observed between the different EC tools. All EC tools perform much better on the smaller bacterial genomes (D1-D5), than on the larger eukaryotes (D6-D8). For all datasets, Karect shows the highest number of true positives (errors that were successfully corrected) and the lowest number of false negatives (uncorrected errors). With the exception of dataset D7 (*C. elegans*) and D8 (*D. melanogaster*), Karect also has the lowest number of false positives (newly introduced errors). Overall, Karect has the highest error correction gain for all datasets.

**Table 3:** Accuracy comparison of EC tools in terms of EC gain, percentage of corrected errors, and number of newly introduced errors per Mbp of read data

	D1	D2	D3	D4	D5	D6	D7	D8
Error correction gain (%)								
ACE	96.3	97.9	98.7	96.2	91.1	41.7	-3.3	25.9
BFC	78.7	84.3	80.2	81.4	78.6	52.8	63.3	24.1
Blue	98.5	98.8	98.7	96.7	95.4	51.1	65.2	28.8
Fiona	87.4	94.6	97.5	85.5	91.4	55.0	65.8	29.8
Karect	99.4	99.8	99.7	98.5	98.2	63.1	75.5	34.3
Lighter	85.4	93.8	92.5	80.1	84.6	45.7	50.3	21.7
Musket	91.3	93.6	93.4	88.0	87.1	49.5	59.2	23.5
RACER	92.3	94.4	97.0	88.3	94.0	17.4	32.6	22.3
SGA-EC	55.3	67.2	45.5	53.1	65.2	48.7	60.6	23.0
Trowel	38.4	49.4	38.8	40.5	46.8	13.2	1.1	10.5
Percentage of corrected errors (sensitivity)								
ACE	97.7	98.5	99.2	98.0	97.0	61.3	73.8	34.5
BFC	78.8	84.4	80.2	81.4	78.7	54.1	63.8	24.7
Blue	98.7	99.3	99.1	97.0	95.7	59.9	70.6	31.4
Fiona	87.5	94.8	97.7	85.5	91.7	60.6	71.7	31.5
Karect	99.4	99.9	99.7	98.5	98.2	64.4	76.7	35.5
Lighter	85.5	94.0	92.7	80.2	86.3	48.9	59.1	24.3
Musket	91.3	93.6	93.4	88.1	87.3	52.9	65.3	26.4
RACER	92.9	95.8	98.2	89.0	94.8	59.2	68.2	34.0
SGA-EC	55.3	67.2	45.5	53.1	65.3	50.4	61.3	23.2
Trowel	39.0	49.9	43.4	40.9	47.6	23.6	31.2	11.8
Number of errors introduced per Mbp								
ACE	44	23	40	151	194	1217	2375	1123
BFC	2	3	7	2	3	83	15	73
Blue	8	20	30	31	10	547	167	341
Fiona	2	7	14	6	9	347	183	218
Karect	0	1	3	1	1	80	36	157
Lighter	2	6	14	8	56	202	273	332
Musket	1	2	5	3	6	214	190	383
RACER	21	62	97	58	27	2603	1097	1524
SGA-EC	1	3	6	2	3	105	22	24
Trowel	21	26	376	41	25	647	930	172

For most datasets, BFC, SGA-EC and Trowel correct significantly fewer sequencing errors compared with other EC tools. BFC and SGA-EC appear to be conservative as they introduce only a small number of new errors. In contrast, ACE, Racer and Trowel often introduce a significant amount of new errors. Note that for dataset D7, the EC gain of ACE is negative, indicating a higher number of sequencing errors after error correction than in the uncorrected data: ACE successfully corrects about 10.8 million errors but introduces almost 11.3 million new errors.

For comparison, *artificial* data was generated for the eight genomes using the same read length and coverage as the corresponding real datasets. Data was corrected using identical settings as before. The confusion matrix and derived metrics can be unambiguously constructed for artificial data since the true, error-free read is known (see Additional file 1: Section 3.2.3). BFC now shows the highest gain for four datasets, while Karect and Fiona each have the highest gain for two datasets. The numbers indicate that EC tools perform much better on artificial data than on real data. This is due to the fact that simulated data are produced according to simplified models that may fail to capture the intricacies of real data.

### Ability of EC tools to improve genome assembly

To evaluate the effect of error correction on *de novo* genome assembly, both uncorrected and corrected reads were assembled using respectively DISCOVAR, IDBA, SPAdes and Velvet. The resulting assemblies were evaluated using QUAST [33] and detailed reports for all combinations of assemblers and EC tools are provided in Additional file 1: Section 4 for reference. We found that SPAdes and DISCOVAR consistently produced higher quality contigs than Velvet and IDBA. We were unable to produce assemblies with DISCOVAR using the reads that were corrected by Trowel and Fiona. Therefore, only SPAdes assemblies are discussed in detail in the remainder of this section.

Table 4 shows the contig and scaffold NGA50 values for all eight datasets and EC tools. For the EC tools that allow the *k*-mer size to be specified, the optimal value of *k* was used (see Additional file 1: Section 1). The NGA50 represents the characteristic length of the assembled contigs/scaffolds that can be contiguously aligned to the reference genome. These contigs/scaffolds thus contain no major structural assembly errors and a higher NGA50 hence implies a less fragmented assembly. For smaller genome sizes (datasets D1-D5), the prior application of EC tools often does not significantly influence the scaffold NGA50. For dataset D3, many tools are able to improve the contig NGA50, sometimes significantly. Remarkably, for dataset D5 (*P. aeruginosa*) most EC tools lead to a somewhat lower scaffold NGA50 compared to the assembly result obtained from uncorrected data. However, the NGAx plot of this dataset reveals no major differences in assembly quality between corrected and uncorrected reads (see Additional file 1: Section 4.3.5). For the larger genomes, the use of EC tools does occasionally improve assembly results, especially on dataset D6 (Human, chr. 21) where eight out of twelve EC tools lead to a higher scaffold NGA50. On the largest datasets D7 and D8 however, error correction may significantly deteriorate the assembly quality. In some cases, the NGA50 obtained is less than half of the corresponding value on uncorrected data.

**Table 4:** NGA50 of respectively contigs (top) and scaffolds (bottom) assembled by SPAdes before and after error correction

Tools	D1	D2	D3	D4	D5	D6	D7	D8
Contig NGA50								
Uncorrected	397 392	92 570	119 253	231 409	264 881	8 559	6 429	50 484
ACE	397 392 =	92 570 =	125 608 *↑*	231 409 =	264 881 =	8 771 *↑*	3 143 $\downdownarrows $	28 679 $\downdownarrows $
BayesHammer	397 392 =	92 344 *↓*	132 564 $\upuparrows $	231 409 =	264 881 =	9 075 *↑*	6 540 *↑*	53 534 *↑*
BFC	397 392 =	92 570 =	132 876 $\upuparrows $	231 409 =	264 881 =	9 375 *↑*	6 389 *↓*	49 185 *↓*
BLESS 2	397 392 =	92 570 =	119 265 *↑*	231 409 =	264 881 =	7 975 *↓*	3 047 $\downdownarrows $	23 814 $\downdownarrows $
Blue	397 392 =	92 708 *↑*	132 876 $\upuparrows $	231 409 =	289 353 *↑*	7 628 $\downdownarrows $	6 191 *↓*	50 486 *↑*
Fiona	397 392 =	92 611 *↑*	119 253 =	231 409 =	264 881 =	9 224 *↑*	5 346 $\downdownarrows $	45 472 *↓*
Karect	397 392 =	92 611 *↑*	132 876 $\upuparrows $	231 409 =	264 881 =	9 865 $\upuparrows $	6 392 *↓*	54 132 *↑*
Lighter	397 392 =	92 570 =	132 564 $\upuparrows $	231 409 =	289 353 *↑*	9 609 $\upuparrows $	6 423 *↓*	50 440 *↓*
Musket	397 392 =	92 566 *↓*	132 876 $\upuparrows $	231 409 =	264 881 =	9 293 *↑*	6 170 *↓*	46 377 *↓*
RACER	397 392 =	92 523 *↓*	112 393 *↓*	231 409 =	264 881 =	7 336 $\downdownarrows $	3 244 $\downdownarrows $	21 538 $\downdownarrows $
SGA-EC	397 392 =	92 344 *↓*	119 255 *↑*	231 409 =	264 881 =	9 296 *↑*	6 435 *↑*	52 105 *↑*
Trowel	397 392 =	92 344 *↓*	119 335 *↑*	231 409 =	264 881 =	7 808 *↓*	6 389 *↓*	48 357 *↓*
Scaffold NGA50								
Uncorrected	397 392	97 353	132 876	231 409	289 353	8 829	6 472	60 554
ACE	397 392 =	97 353 =	133 713 *↑*	231 409 =	264 881 *↓*	9 190 *↑*	3 158 $\downdownarrows $	35 392 $\downdownarrows $
BayesHammer	397 392 =	97 353 =	133 309 *↑*	231 409 =	264 881 *↓*	9 443 *↑*	6 576 *↑*	58 570 *↓*
BFC	397 392 =	97 353 =	133 088 *↑*	231 409 =	264 881 *↓*	9 664 *↑*	6 419 *↓*	59 613 *↓*
BLESS 2	397 392 =	97 353 =	132 876 =	231 409 =	264 881 *↓*	8 441 *↓*	3 073 $\downdownarrows $	35 638 $\downdownarrows $
Blue	397 392 =	97 288 *↓*	133 309 *↑*	231 409 =	289 353 =	7 841 $\downdownarrows $	6 183 *↓*	61 289 *↑*
Fiona	397 392 =	97 353 =	132 876 =	231 409 =	264 881 *↓*	9 491 *↑*	5 385 $\downdownarrows $	54 188 $\downdownarrows $
Karect	397 392 =	97 353 =	133 058 *↑*	231 409 =	264 881 *↓*	10 302 $\upuparrows $	6 446 *↓*	62 304 *↑*
Lighter	397 392 =	97 353 =	133 309 *↑*	231 409 =	289 353 =	9 955 $\upuparrows $	6 468 *↓*	59 697 *↓*
Musket	397 392 =	97 353 =	133 088 *↑*	231 409 =	264 881 *↓*	9 502 *↑*	6 219 *↓*	55 842 *↓*
RACER	397 392 =	97 353 =	132 876 =	231 409 =	264 881 *↓*	7 603 $\downdownarrows $	3 266 $\downdownarrows $	23 783 $\downdownarrows $
SGA-EC	397 392 =	97 353 =	132 876 =	231 409 =	264 881 *↓*	9 640 *↑*	6 483 *↑*	60 636 *↑*
Trowel	397 392 =	97 353 =	132 876 =	231 409 =	264 881 *↓*	8 107 *↓*	6 435 *↓*	57 078 *↓*

Arrows in the table are based on their value relative to the NGA50 value obtained from uncorrected data as follows: $\downdownarrows $ < -10% < *↓* < 0% < *↑* < +10% < $\upuparrows $

**Fig. 2:**
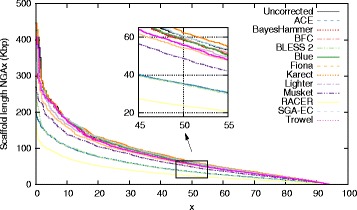
SPAdes assemblies. SPAdes assembly results for *D. melanogaster* for (un)corrected data. Scaffolds with length NGAx or larger contain x% of the genome

Especially for dataset D8 (*D. melanogaster*), the prior use of different EC tools results in a large variability in assembly quality (see Fig. 2). Only Blue, Karect and SGA-EC improve the NGA50 for this dataset. In contrast, error correction with ACE, BLESS 2, Fiona or RACER leads to significantly shorter scaffolds. Additionally, a lower percentage of the genome was found to be covered by scaffolds and a higher rate of insertions, deletions and mismatches was observed (see Additional file 1: Section 4).

At this point it should be stressed that error correction does consistently lead to substantially better assembly results for Velvet or IDBA. However, in our hands, the NGA50 values obtained with Velvet or IDBA were much lower than with SPAdes or DISCOVAR. Even after error correction, Velvet and IDBA yield significantly shorter contigs than SPAdes or DISCOVAR. From this we conclude that the built-in error correction procedures in Velvet and IDBA are less accurate than those in SPAdes and DISCOVAR.

### Error rate versus assembly quality

**Fig. 3:**
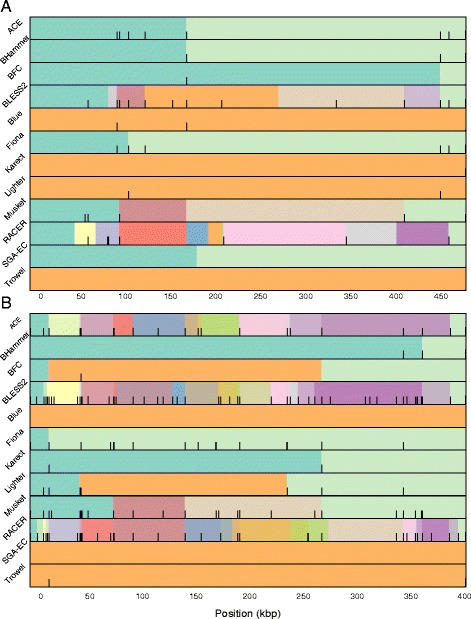
Fragmented assembly using corrected data. Contigs assembled from corrected data are aligned to the largest (*top*) and second largest (*bottom*) contig obtained from uncorrected data. Different colors denote different contigs. *Black bars* indicate the location of lost true *k*-mers in the contigs. This indicates a possible causal relationship between lost true *k*-mers and the breakpoints in the assemblies of corrected data

Even though EC tools almost always reduce the error rate in the input data, they do not necessarily lead to better assemblies. In order to better understand these contrasting observations, we investigated why the use of corrected data can lead to a more fragmented assembly. For the largest dataset (D8), the two largest contigs (> 400 kbp each) that were correctly assembled from uncorrected data were selected. The corresponding (shorter) contigs obtained from assemblies on corrected data were aligned to these contigs and visualized in Fig. 3. With the exception of Trowel, all error correction tools lead to a more fragmented assembly of at least one of these contigs. Breakpoints, i.e., endpoints of the shorter contigs, caused by error correction do not appear to occur at random positions. Rather, different EC tools often cause breakpoints at the same positions. For example, in Fig. 3, the breakpoints marked as ‘A’ and ‘B’ each occur in four cases.

In order to identify the mechanisms that cause breakpoints, the *k*-mer spectrum of both corrected and uncorrected data along the two contigs was examined. In this section, *k*=21 is used throughout, as it corresponds to the smallest *k*-mer size that is used to establish overlap between individual reads by the multi-*k* SPAdes assembler. In Fig. 3, black bars visualize the locations of ‘lost true 21-mers’, i.e., 21-mers that do exist in the reference sequence (hence ‘true’) and also do exist in the uncorrected data but that are no longer present in the corrected data (hence ‘lost’). Lost true *k*-mers hence refer to those *k*-mers that were systematically, but erroneously removed during error correction. In many cases, lost true 21-mers occur in the direct vicinity of breakpoints, indicating a possible causal relationship between lost true 21-mers and these breakpoints (see Fig. 3).

**Fig. 4:**
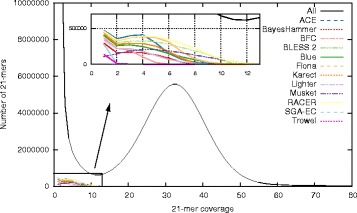
Lost true 21-mers spectrum. For dataset D8, this figure shows the 21-mer spectrum of the uncorrected data, along with the lost true 21-mer spectrum for all EC tools. EC tools erroneously remove low frequency true 21-mers during error correction

To varying degrees, all EC tools suffer from lost true *k*-mers. For dataset D8, Fig. 4 shows the 21-mer spectrum of the uncorrected data, along with the lost true 21-mer spectrum for the individual EC tools. Unsurprisingly, true *k*-mers are almost exclusively lost when their corresponding coverage in the uncorrected data is low. Indeed, a lower than expected coverage is an important feature for EC tools to select candidate errors. Trowel and SGA-EC appear most conservative in terms of lost true *k*-mers: almost no true 21-mers that occur > 2 times are removed. In contrast, ACE, BLESS 2, Musket and RACER remove a significant number of true 21-mers, some of which occur > 10 times in the initial data. These EC tools lead to a more fragmented assembly, which becomes especially evident for the second biggest contig (cfr. Fig. 3).

In principle, a lost true *k*-mer should not necessarily lead to a breakpoint. If all reads that initially contain the lost true *k*-mer(s) are modified in a consistent manner, the assembler will still be able to correctly identify the overlap between those reads and the lost true *k*-mers would appear as mismatches in the resulting assembly. In practice, the lost true *k*-mers will likely be replaced by *k*-mers that actually occur elsewhere in the genome and the genome assembler will be challenged by a spurious repeat that it may or may not be able to resolve. Vice versa, not all breakpoints due to error correction are directly related lost true *k*-mers. The ill-correction of reads could potentially only lead to a decrease in coverage without losing the true *k*-mer in all reads. This can still result in a breakpoint.

**Fig. 5:**
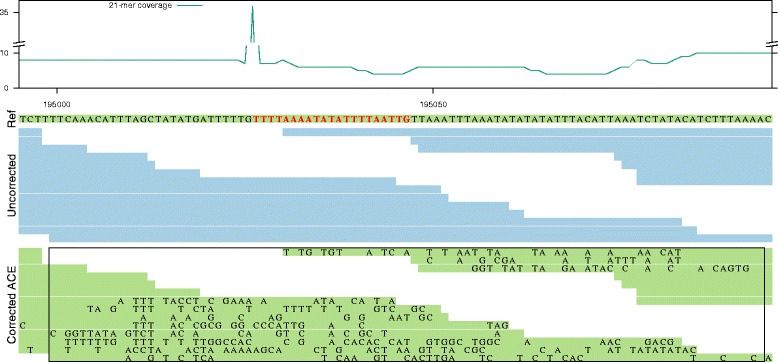
Alignment of uncorrected and ACE-corrected reads in the neighborhood of a contig breakpoint: The first track shows the 21-mer coverage of the uncorrected data. The second track (*Ref*) contains part of the reference genome, which is assembled into one contig from uncorrected data. A repeated 21-mer is indicated in *red*. The third track (*Uncorrected*) shows the alignment of the uncorrected, but error-free reads to the reference. The fourth track (*Corrected*) uses these same alignment positions, but with the sequence content of the corrected reads. Newly introduced errors are indicated by a character in the reads. The *rectangle* in the fourth track indicates 75 overlapping 21-mers that are lost as a result of erroneous error correction

In practice however, we find that breakpoints due to error correction are often related to lost true *k*-mers (cfr. Fig. 3). Further inspection revealed that true *k*-mers are typically lost in regions that suffer from poor coverage in the direct vicinity of a local coverage peak. Often, such sudden increase in coverage is caused by the presence of a short repeated element. For example, Fig. 5 shows a genomic region with low *k*-mer coverage (around 7 X) that contains a repeated *k*-mer with coverage 35. This repeated *k*-mer also occurs in other reads that originate from different genomic locations. We can therefore assume that the EC tool makes erroneous decisions based on the sequence content of these reads. In this example, ACE makes a large number of substitutions in originally error-free reads causing 75 consecutive lost true *k*-mers. Clearly, the error correction procedure is not performed in a consistent manner for all reads, rendering the assembler unable to detect overlap between these reads and ultimately leading to a breakpoint. For the same reasons, BLESS 2 and RACER also break at this specific location.

**Fig. 6:**
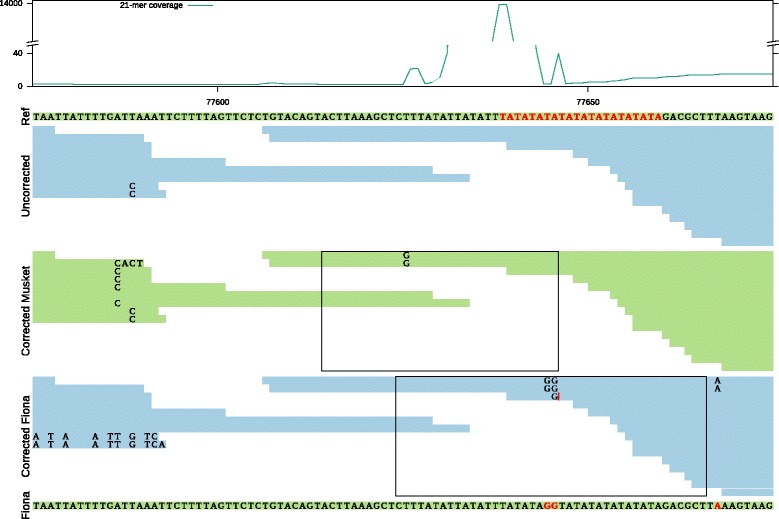
Alignment of uncorrected and corrected reads by Musket and Fiona in the neighborhood of a contig breakpoint: Lost true *k*-mer can result in two different scenarios. The first track shows the 21-mer coverage of the uncorrected data. The second track (*Ref*) shows a part of the reference genome, which is assembled into one contig from uncorrected data. A frequently occurring AT-repeat is indicated in *red*. The third track (*Uncorrected*) shows the alignment of the uncorrected reads to the reference. The fourth and the fifth tracks (*Corrected Musket* and *Corrected Fiona*) use these same alignment positions, but with the sequence content of corrected reads by Musket and Fiona. The sixth track is the assembled contig from corrected reads by Fiona. The *rectangles* indicate the regions in corrected reads by Musket and Fiona that no longer contain any true 21-mers. The coverage is low around an ‘AT’ repeat with coverage 13750x in the uncorrected data. Musket incorrectly changed two bases, breaking the connection between two groups of reads. In contrast, in the Fiona-corrected reads, the connection is not lost. Instead the lost true *k*-mers in Fiona appear as mismatches in the assembled contig

As a second example, Fig. 6 shows a short 22 bp long AT repeat with very high coverage (nearly 14 000 X), in a genomic region with otherwise low coverage. Musket introduces a new error in two out of four overlapping reads. Within this specific context, these substitutions cause a number of true *k*-mers to be lost. More importantly, because the error correction is not performed in an identical manner across all four reads overlapping this locus, the overlap is broken and a breakpoint is introduced. Similarly, due to the same AT repeat, Fiona introduces errors that result in a number of lost true *k*-mers. In this case however, the newly introduced errors result in mismatches in the assembled sequence rather than a breakpoint.

From these examples, the limitations of *k*-mer spectrum based error correction tools become evident. Due to their primary focus on individual *k*-mers, they do not take into account the surrounding context in which the *k*-mer occurs. Because these tools correct reads individually, different corrections may be applied to different reads even though the reads overlap the same genomic region. This may render de Bruijn graph assemblers unable to detect overlap between those reads. In that respect, error correction tools that rely on multiple sequence alignments (MSA) are in principle less susceptible to this kind of error. As overlapping reads are clustered and aligned, the error correction is systematic across those reads. MSA-based tools indeed yield higher NGA50 values on average.

These results demonstrate that evaluating error correction tools directly on their ability to reduce error rate has significant limitations as there is often no clear correlation between such metrics and the ability to improve assembly. For example, on datasets D8, ACE ranked fourth in terms of gain and showed the highest number of corrected reads that align error-free to the reference genome. Yet, ACE-corrected reads do not lead to good assembly results on this dataset.

We should emphasize that error correction is not always destructive: EC tools can improve the quality of assembly in certain cases. For example, even though Karect also suffers from a significant number of ‘lost true *k*-mers’ (see Fig. 4), the tool leads to the highest NGA50 values in many cases (see Table 4). Again for dataset D8, we selected the longest contig (> 500 kbp) that was correctly assembled from corrected data by Karect and aligned the corresponding (shorter) contigs obtained from assemblies on uncorrected data. A specific case where Karect removes errors that subsequently lead to the correct connection between two contigs is shown in Additional file 1: Section 5.

### Time and space requirements

**Fig. 7:**
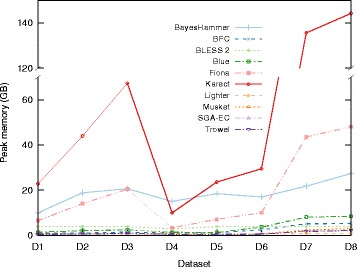
Peak memory usage of the EC tools

**Fig. 8:**
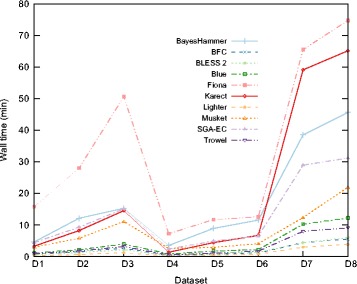
Runtime of the EC tools

Figures 7 and 8 show the memory usage and runtime of the EC tools (see Additional file 1: Section 6.1 for detailed tables). Since it is not possible to specify the number of threads for ACE and RACER, they were omitted. For all datasets, BayesHammer, Fiona and Karect use significantly more memory than other tools while BayesHammer, Fiona, Karect, Musket, and SGA-EC have a relatively high runtime. In general, we note that all tools that rely on multiple sequence alignments require more resources. The tools that rely on Bloom filters (BLESS 2, Lighter and BFC) are both memory efficient and fast.

Given the reduced error in the input data, we evaluate the potential of error correction tools to reduce the peak memory usage and/or runtime of the assembly process itself. Since error correction is computationally intensive, this may be an important aspect of error correction tools. Peak memory usage and runtime were measured for all assemblies with SPAdes and DISCOVAR (Additional file 1: Figures S3–S6). The runtime of DISCOVAR shows no decrease after error correction, while the peak memory usage decreases slightly. Conversely, the runtime of SPAdes does decrease after error correction, but the peak memory usage does not.

The peak memory usage and runtime tables for artificial data show that Lighter and SGA-EC are again among the most memory-efficient tools, while Karect and Fiona consume more memory than any other tools. Lighter is the fastest tool followed by BLESS 2 in all the cases (Additional file 1: Section 6.2).

## Conclusions

The performance of different EC tools was compared using two approaches: the ability of EC tools to correct sequencing errors in Illumina data, and the effects of those corrections on the resulting *de novo* genome assembly quality. We found that EC tools correct a significant fraction of sequencing errors. However, state-of-the-art Illumina assemblers do not always appear to benefit from this. The assembly results for eight different datasets with SPAdes and DISCOVAR show that the prior application of EC tools often does not lead to a significant increase in NGA50, and in fact may result in a lower NGA50. Many erroneous corrections occur in regions that have low read coverage and in the vicinity of highly frequent repeats. Due to the low coverage, error correction tools incorrectly assume the presence of sequencing errors. The repeated elements on the other hand cause erroneous substitutions to be applied. A too aggressive and/or inconsistent transformation of such reads in such region may lead to loss of information from which no recovery is possible during the assembly process. This inevitably leads to an increased assembly fragmentation. Additionally, the prior use of EC tools does not lead to a major decrease in overall runtime and/or memory requirements compared with the assembly from uncorrected data.

From a methodological point of view, multiple sequence alignment (MSA) based methods might have an advantage over methods that operate on isolated *k*-mers. MSA-based methods take multiple reads into account when applying substitutions and hence appear to make more consistent corrections across overlapping reads.

We recommend future EC tools to be primarily evaluated on their ability to improve assembly results using state-of-the-art assemblers and sufficiently large datasets. Only a relatively small fraction of sequencing errors are truly impacting the assembly process. It is the behavior of the error correction tool on precisely these cases that will ultimately determine its degree of success.

## Additional file

Additional file 1 Supplementary Data. Evaluation of the impact of Illumina error correction tools on de novo genome assembly. (PDF 628 kb)

## Abbreviations

bp: Base pair
D1…D8: Dataset 1…8
EC: Error correction
FN: False negative
FP: False positive
GB: Gigabyte
GHz: Gigahertz
indel: Insertion or deletion
kbp: Kilobase pair
Mbp: Megabase pair
MSA: Multiple sequence alignment
TN: True negative
TP: True positive
TPR: True positive rate
