# Supplementary material for: Evaluation of the impact of Illumina error correction tools on de novo genome assembly
Source: BMC Bioinformatics. 2017 Aug 18;18:374. doi: 10.1186/s12859-017-1784-8 (PMC5563063; doi:10.1186/s12859-017-1784-8)
Supplement: Additional file 1 — Supplementary Data. Evaluation of the impact of Illumina error correction tools on de novo genome assembly. (PDF 628 kb) [file 12859_2017_1784_MOESM1_ESM.pdf]

Supplementary Data: Evaluation of the Impact of Illumina Error Correction Tools on de novo Genome Assembly

Mahdi Heydari[1,4], Giles Miclotte[1,4], Piet Demeester[1,4],
Yves Van de Peer[2,3,4,5], and Jan Fostier[1,4]

[1]Department of Information Technology, Ghent University-imec, IDLab, Ghent, Belgium
[2]Center for Plant Systems Biology, VIB, Ghent, Belgium
[3]Department of Plant Biotechnology and Bioinformatics, Ghent University, Ghent, Belgium
[4]Bioinformatics Institute Ghent, Ghent, Belgium
[5]Department of Genetics, Genome Research Institute, University of Pretoria, Pretoria, South Africa

Contact: jan.fostier@ugent.be

Contents

1 Error Correction Tool Parameter Settings **4**
1.1 ACE . . . 4
1.2 BayesHammer v. 3.7.1 . . . 4
1.3 BFC v. r181 . . . 4
1.4 BLESS 2 v. 1.02 . . . 4
1.5 Blue v. 1.1.2 . . . 5
1.6 Fiona v. 0.2.5 . . . 5
1.7 Karect v. 1.0 . . . 5
1.8 Lighter v. 1.1.0 . . . 6
1.9 Musket v. 1.1 . . . 6
1.10 RACER v. 1.0.1 . . . 6
1.11 SGA-EC v. 0.10.14 . . . 6
1.12 Trowel v. 0.2.0.4 . . . 6

2 Data simulation **8**

3 Error Metrics **8**
3.1 Alignment ratio . . . 8
3.2 EC gain . . . 9
3.2.1 Accuracy comparison method . . . 9
3.2.2 Real Data . . . 10
3.2.3 Simulated Data . . . 11

4 Assembly Result for Real Data **13**
4.1 DISCOVAR . . . 14
4.1.1 *B. dentium* . . . 14
4.1.2 *E. coli str. K-12 substr. DH10B* . . . 15
4.1.3 *E. coli str. K-12 substr. MG1655* . . . 16
4.1.4 *S. enterica* . . . 17
4.1.5 *P. aeruginosa* . . . 18
4.1.6 *H. sapiens* Chr. 21 . . . 19
4.1.7 *C. elegans* . . . 20
4.1.8 *D. melanogaster* . . . 21
4.2 IDBA . . . 22
4.2.1 *B. dentium* . . . 22
4.2.2 *E. coli str. K-12 substr. DH10B* . . . 23
4.2.3 *E. coli str. K-12 substr. MG1655* . . . 24
4.2.4 *S. enterica* . . . 25
4.2.5 *P. aeruginosa* . . . 26
4.2.6 *H. sapiens* Chr. 21 . . . 27
4.2.7 *C. elegans* . . . 28
4.2.8 *D. melanogaster* . . . 29
4.3 SPAdes . . . 29
4.3.1 *B. dentium* . . . 29
4.3.2 *E. coli str. K-12 substr. DH10B* . . . 31
4.3.3 *E. coli str. K-12 substr. MG1655* . . . 32
4.3.4 *S. enterica* . . . 33
4.3.5 *P. aeruginosa* . . . 34
4.3.6 *H. sapiens* Chr. 21 . . . 36
4.3.7 *C. elegans* . . . 37
4.3.8 *D. melanogaster* . . . 38

4.4 Velvet . . . 39
4.4.1 *B. dentium* . . . 39
4.4.2 *E. coli str. K-12 substr. DH10B* . . . 40
4.4.3 *E. coli str. K-12 substr. MG1655* . . . 41
4.4.4 *S. enterica* . . . 42
4.4.5 *P. aeruginosa* . . . 43
4.4.6 *H. sapiens* Chr. 21 . . . 44
4.4.7 *C. elegans* . . . 45
4.4.8 *D. melanogaster* . . . 46

5 Ability of EC tools to improve genome assembly 47

6 Memory and Runtime 48
6.1 Real Data . . . 48
6.1.1 Memory . . . 48
6.1.2 Runtime . . . 48
6.2 Simulated Data . . . 51
6.2.1 Memory . . . 51
6.2.2 Runtime . . . 51

1 Error Correction Tool Parameter Settings

All error correction tools were executed with 32 threads. Some tools need to be provided with the approximate genome size. For those tools, the exact genome size was provided. Some tools that internally operate on k-mers allow the user to specify the value of k. For all tables and figures in the main paper and in supplementary data, except Table 4 in the main paper, the default or recommended value of k was taken for each tool, regardless of dataset or assembly tool that was used.

To generate the results of Table 4 in the main paper, the optimal value of k was selected for each EC tool/dataset combination by running the EC tool multiple times with different k-mer sizes. The optimal k-mer size then corresponds to the SPAdes assembly that yields the highest scaffold N50. This way of selecting the optimal value of k would be identical to an end-user who wants to optimally assemble a new genome in absence of a reference genome. Below, the actual parameters are specified for each tool individually:

1.1 ACE

The k-mer size for ACE is built-in and cannot be specified by the user.

```
$ size=$(stat -c%s genome.fasta)
$ ./ace $size $inputreads aceOut/aceCorrected
```

1.2 BayesHammer v. 3.7.1

The k-mer size for BayesHammer is built-in and cannot be specified by the user.

```
$ ./spades.py -t 32 --careful --12 $inputreads -o bayesHammerOut
--only-error-correction --disable-gzip-output
```

1.3 BFC v. r181

```
$ size=$(stat -c%s genome.fasta)
$ ./bfc -s $size -k 33 -t 32 $inputreads >bfcOut/bfcCorrected
```

Following table shows the scaffold N50 of the SPAdes assembly from BFC-corrected reads for different values of k used in BFC.

k-mer size	D1	D2	D3	D4	D5	D6	D7	D8
29	287 949	108 254	133 309	723 537	289 353	**16 529**	**7 879**	82 402
31	287 949	108 254	133 309	723 537	289 353	16 294	7 869	80 872
33	**287 949**	**108 254**	**133 309**	**723 537**	**289 353**	16 484	7 871	82 203
35	287 949	107 839	133 309	723 537	289 353	16 273	7 857	**83 364**
37	287 949	107 839	133 309	723 537	289 353	15 856	7 865	82 486

Therefore, in Table 4 of the main paper, $k = 33$ was used for datasets D1, D2, D3, D4 and D5; $k = 29$ was used for datasets D6 and D7; $k = 35$ was used for dataset D8. The default value of $k = 33$ was used for all other tables and figures.

1.4 BLESS 2 v. 1.02

```
$ ./bless -read $inputreads -prefix blessOut/blessCorrected
-kmerlength 31 -smpthread 32
```

Following table shows the scaffold N50 of the SPAdes assembly from BLESS-corrected reads for different values of k used in BLESS 2.

k-mer size	D1	D2	D3	D4	D5	D6	D7	D8
27	397 392	**108 357**	**133 309**	381 567	264 881	**14 672**	3 894	44 783
29	397 392	108 254	**133 309**	381 537	264 881	13 970	**3 996**	**45 633**
31	**397 392**	108 254	126 410	381 864	264 881	13 678	3 931	43 315
33	397 392	108 254	126 410	**412 097**	264 881	14 267	3 834	41 579
35	397 392	108 254	126 410	412 031	**289 353**	14 180	3 723	41 677

Therefore, in Table 4 of the main paper, $k = 27$ was used for datasets D2, D3 and D6; $k = 29$ was used for datasets D7 and D8; $k = 33$ was used for dataset D4; $k = 35$ was used for dataset D5. The default value of $k = 31$ was used for all other tables and figures.

1.5 Blue v. 1.1.2

```
$ size=$(stat -c%s genome.fasta)
$ mono Tessel.exe -k 25 -g $size -t 32 Cspor $inputreads
$ mono Blue.exe -r blueCorrected -t 32 -o blueOut Cspor_31.cbt reads
```

Following table shows the scaffold N50 of the SPAdes assembly from Blue-corrected reads for different values of k used in Blue.

k-mer size	D1	D2	D3	D4	D5	D6	D7	D8
21	**292 264**	107 776	133 088	723 550	**314 485**	13 572	7 454	77 807
25	287 948	107 774	**133 309**	**723 550**	289 314	13 214	**7 708**	83 277
27	287 948	**108 189**	133 309	723 366	289 314	**13 690**	7 686	84 876
29	287 948	**108 189**	133 309	723 537	289 314	13 397	7 685	85 463
31	287 948	**108 189**	133 309	723 537	289 314	13 300	7 682	**86 523**

Therefore, in Table 4 of the main paper, $k = 21$ was used for datasets D1 and D5; $k = 25$ was used for datasets D3, D4 and D7; $k = 27$ was used for dataset D6; $k = 31$ was used for datasets D2 and D8. The default value of $k = 25$ was used for all other tables and figures.

1.6 Fiona v. 0.2.5

The k-mer size for Fiona is built-in and cannot be specified by the user.

```
$ size=$(stat -c%s genome.fasta)
$ ./fiona -nt 32 -g $size $inputreads fionaOut/fionaCorrected
```

1.7 Karect v. 1.0

```
$ ./karect -correct -inputfile=$inputreads -matchtype=hamming
-celltype=diploid -resultdir=karectOut -kmer=9 -memory=32 -threads=32
```

Following table shows the scaffold N50 of the SPAdes assembly from Karect-corrected reads for different values of k used in Karect.

k-mer	D1	D2	D3	D4	D5	D6	D7	D8
9	**287 949**	**108 228**	**133 309**	**725 282**	**289 353**	**17 170**	7 923	**88 533**
11	287 949	108 228	133 309	725 282	289 353	17 170	7 923	88 533
13	287 949	108 228	133 309	723 537	289 353	16 805	**7 944**	88 135
14	287 949	108 228	133 309	723 537	289 353	16 866	7 917	87 347

Therefore, in Table 4 of the main paper, $k = 9$ was used for datasets D1, D2, D3, D4, D5, D6 and D8; $k = 13$ was used for dataset D7; The default value of $k = 9$ was used for all other tables and figures. It should be noted that Karect sometimes overrides the user-specified value for k to a value it considers to be more suitable.

1.8 Lighter v. 1.1.0

```
$ size=$(stat -c%s genome.fasta)
$ ./lighter -t 32 -K 17 $size -r $inputreads -od lighterOut/lighterCorrected
```

Following table shows the scaffold N50 of the SPAdes assembly from Lighter-corrected reads for different values of k used in Lighter.

k-mer	D1	D2	D3	D4	D5	D6	D7	D8
13	287 949	**108 254**	133 309	412 163	**289 353**	15 197	**7 946**	83 760
15	287 949	107 839	133 309	723 537	289 353	16 096	7 599	**84 674**
17	**287 949**	107 839	**133 309**	**723 537**	264 881	16 666	7 154	79 363
19	287 949	107 839	133 309	723 537	289 353	16 734	7 455	80 331
21	287 949	107 839	133 309	723 537	289 353	**16 826**	7 726	82 346

Therefore, in Table 4 of the main paper, $k = 13$ was used for datasets D2, D5 and D7; $k = 15$ was used for dataset D8; $k = 17$ was used for datasets D1, D3 and D4; $k = 21$ was used for dataset D6. The default value of $k = 17$ was used for all other tables and figures.

1.9 Musket v. 1.1

```
$ ./musket -inorder -p 32 $inputreads -o musketOut/musketCorrected
```

Following table shows the scaffold N50 of the SPAdes assembly from Musket-corrected reads for different values of k used in Musket.

k-mer	D1	D2	D3	D4	D5	D6	D7	D8
17	287 949	107 839	133 309	723 537	**289 353**	16 152	5 808	58 138
21	**287 949**	107 839	**133 309**	**723 537**	264 881	**16 419**	6 334	63 519
23	287 949	107 839	125 608	723 537	**289 353**	16 414	7 190	70 009
25	287 949	**108 254**	125 608	723 537	**289 353**	16 260	7 598	69 990
27	287 949	**108 254**	133 309	723 537	**289 353**	15 816	**7 704**	**75 521**

Therefore, in Table 4 of the main paper, $k = 21$ was used for datasets D1, D3, D4 and D6; $k = 27$ was used for dataset D2, D5, D7 and D8. The default value of $k = 21$ was used for all other tables and figures.

1.10 RACER v. 1.0.1

The k-mer size for RACER is built-in and cannot be specified by the user.

```
$ size=$(stat -c%s genome.fasta)
$ ./racer.exe $inputreads racerOut/Corrected $size
```

1.11 SGA-EC v. 0.10.14

No k-mer value has to be specified for SGA-EC.

```
$ ./sga preprocess --permute-ambiguous --no-primer-check -o sgaOut/temp
-p=2 -m 11 $inputreads
$ ./sga index -a ropebwt -t 32 --no-reverse sgaOut/temp
$ ./sga correct --learn -t 32 -o sgaOut/sgaCorrectede sgaOut/temp
```

1.12 Trowel v. 0.2.0.4

```
$echo $inputreads> trowelOut/trowelInput
$ ./trowel.0.2.0.4.linux.64 -k 11 -t 32 -f trowelOut/trowelCorrected
```

Following table shows the scaffold N50 of the SPAdes assembly from Trowel-corrected reads for different values of k used in Trowel.

k-mer	D1	D2	D3	D4	D5	D6	D7	D8
11	**287 948**	**108 254**	**133 309**	**723 537**	**289 353**	13 493	7 913	77 879
13	287 948	108 254	133 189	723 537	289 353	14 000	**7 938**	77 308
15	287 948	108 254	133 309	723 537	289 353	**14 042**	7 909	**79 217**

Therefore, in Table 4 of the main paper, $k = 11$ was used for datasets D1, D2, D3, D4 and D5; $k = 13$ was used for dataset D7; $k = 15$ was used for datasets D6 and D7. The default value of $k = 11$ was used for all other tables and figures.

2 Data simulation

To compare the performance of error correction tools (EC tools) on simulated data, we produced synthetic Illumina reads for the same set of organisms for which real data was used. The ART read simulator is used to generate reads, with the following command:

```
$ ./art_illumina -i genome.fasta -p -l [len] -f [cov] -m 300 -s 30 -o reads
```

Where `cov` and `len` correspond to the coverage and length and change according to the values in real datasets. The mean fragment size is 300 bp, and the fragment standard deviation is 30 bp.

3 Error Metrics

3.1 Alignment ratio

Reads are grouped based on the number of mismatches (m) after aligning them to the reference genome using BWA v. 0.7.12:

```
$ ./bwa mem -M -t 32 -p reference/genome.fasta reads.fastq
>alignment/samfileName.sam
```

Table 1 shows the percentage of reads that align to the reference genome without mismatches ($m = 0$). Table 2 shows the percentage of reads that do not align to the reference genome with <10 mismatches. The 'Uncorrected' row shows the results of the raw data.

Table 1: Percentage of reads that mapped with 0 mismatches (%).

Tool	*B. dentium*	*E. coli DH10B*	*E. coli MG1655*	*S. enterica*	*P. aeruginosa*	*H. sapiens* Chr. 21	*C. elegans*	*D. melanogaster*
Uncorrected	70.16	76.31	61.07	55.17	83.64	69.36	70.41	50.32
ACE	98.04	98.83	98.83	94.61	98.75	82.33	79.09	60.14
BayesHammer	93.40	95.77	89.37	90.15	95.95	81.46	78.01	57.75
BFC	97.41	98.86	96.65	92.35	98.48	83.09	78.57	59.82
BLESS 2	96.86	98.43	96.48	90.14	98.24	83.46	78.48	59.66
Blue	98.58	99.64	99.15	94.23	99.11	82.28	78.41	60.07
Fiona	96.26	97.93	96.61	89.49	97.64	82.16	78.11	59.23
Karect	98.04	99.29	98.84	93.94	98.51	83.17	79.07	59.97
Lighter	96.72	98.58	97.87	89.63	97.09	81.36	77.15	58.70
Musket	97.67	98.99	96.82	92.17	98.40	81.78	77.80	59.72
RACER	97.30	98.58	98.01	91.40	98.40	82.21	78.51	59.69
SGA-EC	90.14	93.76	78.88	86.35	94.97	81.51	78.06	58.06
Trowel	85.63	90.76	82.72	79.01	92.37	75.85	74.68	54.90

Table 2: Percentage of reads that do not align with <10 mismatches.

Tool	*B. dentium*	*E. coli DH10B*	*E. coli MG1655*	*S. enterica*	*P. aeruginosa*	*H. sapiens* Chr. 21	*C. elegans*	*D. melanogaster*
Uncorrected	2.76	0.95	0.39	9.99	0.90	0.62	18.11	6.80
ACE	0.33	0.01	0.11	3.20	0.28	0.33	17.12	6.34
BayesHammer	1.27	0.15	0.02	3.33	0.40	0.22	16.69	6.47
BFC	1.45	0.19	0.30	5.20	0.66	0.54	18.05	6.67
BLESS 2	1.32	0.19	0.49	5.07	0.66	0.61	18.07	6.84
Blue	1.09	0.03	0.20	4.31	0.49	0.44	17.88	6.60
Fiona	1.48	0.11	0.17	5.40	0.51	0.41	17.78	6.60
Karect	1.11	0.02	0.17	3.88	0.43	0.39	17.73	6.58
Lighter	1.47	0.10	0.19	5.45	0.59	0.47	17.84	6.63
Musket	1.34	0.15	0.26	4.96	0.62	0.47	17.89	6.60
RACER	1.31	0.14	0.23	4.88	0.51	0.45	17.70	6.65
SGA-EC	2.54	0.76	0.35	9.14	0.75	0.55	18.06	6.70
Trowel	1.95	0.56	0.23	7.03	0.69	0.53	17.69	6.70

3.2 EC gain

3.2.1 Accuracy comparison method

Accuracy comparison of EC tools in simulated data is straightforward since the perfect read is known. Let R represent an input read. For each read R, there is a corresponding read C which is corrected by any of the EC tools. In artificial data, a perfect read P is provided together with R. Therefore, for the evaluation of tools in simulated data, bases in these three reads (R, P and C) are compared and classified as follows:

$$\begin{aligned}
tp &: \mathrm{R}_c \neq \mathrm{P}_c \text{ and } \mathrm{C}_c = \mathrm{P}_c,\ \mathrm{TP} = \sum_{c \in R} tp; \\
tn &: \mathrm{R}_c = \mathrm{P}_c \text{ and } \mathrm{C}_c = \mathrm{P}_c,\ \mathrm{TN} = \sum_{c \in R} tn; \\
fp &: \mathrm{R}_c = \mathrm{P}_c \text{ and } \mathrm{C}_c \neq \mathrm{P}_c,\ \mathrm{FP} = \sum_{c \in R} fp; \\
fn &: \mathrm{R}_c \neq \mathrm{P}_c \text{ and } \mathrm{C}_c \neq \mathrm{P}_c,\ \mathrm{FN} = \sum_{c \in R} fn.
\end{aligned}$$

The error correction gain (EC gain) is then defined as

$$\text{EC gain} = \frac{\mathrm{TP} - \mathrm{FP}}{\mathrm{TP} + \mathrm{FN}}.$$

An EC gain of 100% means all errors were corrected and no new errors were introduced. Additionally, sensitivity and false positive rate (FP rate) are defined as follows:

$$\begin{aligned}
\text{Sensitivity} &= \frac{\mathrm{TP}}{\mathrm{TP} + \mathrm{FN}}, \\
\text{FP rate} &= \frac{\mathrm{FP}}{\mathrm{TN} + \mathrm{FP}}.
\end{aligned}$$

3.2.2 Real Data

Exact numbers of TP, TN, FP and FN for real data are shown in Table 3.

Table 3: Detailed confusion matrices for real data.

	**Dataset**							
Tool	*B. dentium*	*E. coli DH10B*	*E. coli MG1655*	*S. enterica*	*P. aeruginosa*	*H. sapiens* Chr. 21	*C. elegans*	*D. melanogaster*
**True Positives (TP) – corrected errors**								
ACE	3076751	8332547	22291105	3701334	3292214	5032509	10795486	25245221
BFC	2482403	7142877	18036534	3083801	2670043	4444675	9330314	18081922
Blue	3112185	8400946	22279091	3674979	3246700	4915665	10326983	22965194
Fiona	2757539	8018906	21957781	3239983	3111415	4978875	10486053	23052956
Karect	3133184	8450316	22414636	3732389	3331053	5287884	11220434	25992487
Lighter	2694538	7952212	20837800	3038404	2927161	4014919	8650557	17759054
Musket	2878390	7922398	21005281	3335539	2961995	4345534	9554662	19344147
RACER	2932759	8112897	22077431	3374675	3215041	4865921	9987137	24864167
SGA-EC	1742936	5690254	10231654	2010468	2215005	4137311	8965373	16986433
Trowel	1229337	4226181	9761215	1550337	1613647	1936362	4575638	8640337
**True Negatives (TN) – initially correct bases left untouched**								
ACE	963422263	1931235385	2746256917	440139607	1039050327	1319616601	4737356765	5606869096
BFC	964033360	1931274464	2746348400	441245208	1039248109	1321102045	4748425050	5612713036
Blue	964029362	1931244564	2746290398	441235585	1039242118	1320502326	4747747773	5611271694
Fiona	964035021	1931270828	2746332983	441244937	1039242743	1320774172	4747710215	5611944730
Karect	964034920	1931277673	2746357548	441245411	1039250137	1321107050	4748328371	5612244594
Lighter	964033340	1931268196	2746328586	441242591	1039192194	1320945136	4747204149	5611259898
Musket	964034290	1931275869	2746352663	441244510	1039244912	1320929786	4747597357	5610975777
RACER	964010310	1931159567	2746106691	441215449	1039223611	1317855370	4743412687	5604761363
SGA-EC	964034237	1931275385	2746351399	441245198	1039248037	1321074542	4748393243	5612994706
Trowel	964015541	1931230735	2745348701	441227802	1039224947	1320368303	4744149598	5612170398
**False Positives (FP) – newly introduced errors**								
ACE	42115	45236	110238	66473	201973	1607588	11276793	6306244
BFC	2017	5828	18069	721	2751	110312	73154	411719
Blue	7660	38812	81267	13731	9982	722472	795185	1916850
Fiona	1939	12563	37718	2501	9327	458681	867700	1224861
Karect	457	2624	8951	518	723	105756	171201	881629
Lighter	2038	12099	37887	3339	58686	267293	1294892	1865549
Musket	1087	4430	13816	1419	5951	282661	901130	2149247
RACER	20414	119807	266323	25624	27673	3439109	5209526	8553829
SGA-EC	1143	4912	15115	735	2890	138742	106266	134578
Trowel	19913	49616	1032119	18304	26030	854611	4415136	964727
**False Negatives (FN) – remaining errors**								
ACE	72832	129122	189116	76350	100688	3178135	3838512	47947216
BFC	669265	1318746	4443590	704083	722347	3763717	5294722	55097487
Blue	39497	60730	201157	112984	145698	3293805	4298919	50218318
Fiona	393729	442232	522381	547490	280918	3236482	4143215	50179596
Karect	18484	11307	65491	55495	61337	2920538	3404740	47187117
Lighter	457130	509413	1642324	749481	465237	4193495	5974735	55420668
Musket	273278	539225	1474842	452345	430394	3862860	5070408	53835292
RACER	223971	352560	403619	418534	177627	3351498	4654443	48340247
SGA-EC	1408740	2771371	12248650	1777441	1177881	4071424	5660045	56205417
Trowel	1922344	4235705	12724566	2237577	1778750	6274148	10071360	64540776

3.2.3 Simulated Data

Table 4 shows the EC gain, sensitivity, and specificity expressed as number of errors introduced per Mbp of read data. Table 5 shows the exact numbers of TP, TN, FP and FN for all tools on the simulated data. BFC has the highest gain on four datasets, Karect and Fiona both have the highest gain on two datasets.

Table 4: Accuracy comparison in terms of EC gain, percentage of corrected errors, and number of errors introduced per Mbp in simulated data.

	Dataset							
Tool	*B. dentium*	*E. coli DH10B*	*E. coli MG1655*	*S. enterica*	*P. aeruginosa*	*H. sapiens* Chr. 21	*C. elegans*	*D. melanogaster*
**Error correction gain (%)**								
ACE	99.8	99.7	99.8	99.9	99.2	81.2	74.3	95.8
BFC	99.9	99.8	98.7	99.9	99.8	96.2	99.3	99.7
Blue	99.7	99.0	99.1	99.7	99.5	85.1	91.0	97.2
Fiona	99.9	99.9	99.7	99.8	99.9	90.3	96.2	98.1
Karect	99.9	99.8	99.9	99.9	99.8	92.8	98.0	99.4
Lighter	99.3	99.6	99.5	99.2	98.2	84.8	83.6	90.0
Musket	99.8	99.7	99.8	99.8	99.4	88.5	93.3	97.9
RACER	99.2	98.9	98.8	99.2	98.6	58.9	69.1	87.6
SGA-EC	99.6	99.7	15.8	99.8	99.8	89.8	96.8	97.6
Trowel	84.1	83.5	74.4	85.7	81.6	67.9	76.1	75.8
**Sensitivity – percentage of corrected errors (%)**								
ACE	100.0	99.9	99.9	100.0	99.9	94.3	98.5	98.7
BFC	99.9	99.8	98.7	99.9	99.9	97.8	99.4	99.8
Blue	100.0	100.0	99.6	100.0	100.0	91.3	97.3	98.3
Fiona	99.9	99.9	99.8	99.9	99.9	95.6	98.8	98.6
Karect	100.0	100.0	100.0	100.0	100.0	93.1	99.3	99.5
Lighter	99.4	99.7	99.6	99.3	98.9	88.6	89.3	92.7
Musket	99.8	99.7	99.8	99.8	99.5	90.0	94.1	98.2
RACER	99.9	99.9	99.9	99.9	99.9	93.0	97.7	98.6
SGA-EC	99.6	99.7	15.9	99.8	99.9	92.1	97.8	97.8
Trowel	84.3	83.5	74.6	85.8	81.6	70.0	76.8	76.0
**Number of errors introduced per Mbp of read data**								
ACE	12	4	10	9	12	1271	389	280
BFC	1	0	1	1	0	156	2	8
Blue	25	19	43	28	8	603	102	105
Fiona	3	2	13	5	1	520	41	49
Karect	5	3	4	5	2	35	22	13
Lighter	5	1	11	7	13	368	91	267
Musket	1	0	2	1	1	147	13	28
RACER	71	20	101	60	24	3312	460	1063
SGA-EC	1	1	6	2	1	221	16	19
Trowel	18	1	13	6	0	203	11	16

Table 5: Detailed confusion matrices in simulated data

	Dataset							
Tool	*B. dentium*	*E. coli DH10B*	*E. coli MG1655*	*S. enterica*	*P. aeruginosa*	*H. sapiens* Chr. 21	*C. elegans*	*D. melanogaster*
				**True Positives (TP) – corrected errors**				
ACE	9667046	3814496	27260933	4359012	1847493	12266571	9180759	59347214
BFC	9659287	3808751	26926157	4357828	1846499	12728393	9263433	59962209
Blue	9666564	3815467	27161340	4359254	1848540	11881359	9070130	59069399
Fiona	9660940	3815149	27232134	4356236	1847967	12446652	9208110	59246494
Karect	9669223	3815493	27270690	4359797	1848298	12116343	9257419	59816346
Lighter	9608363	3806153	27164122	4328575	1828813	11529659	8318765	55726718
Musket	9650131	3805811	27216841	4352679	1839474	11713659	8768168	59020638
RACER	9676980	3815294	27244781	4362894	1847896	12114016	9116775	59266390
SGA-EC	9632731	3806414	4340616	4351854	1846414	11986144	9115211	58761640
Trowel	8151159	3188984	20336922	3740932	1508617	9109108	7155992	45677232
				**True Negatives (TN) – initially correct bases left untouched**				
ACE	973643656	1954926851	2812136997	469845584	1056817153	1339880180	5805025579	6198006667
BFC	973654353	1954933880	2812161687	469849372	1056829305	1341366583	5807245715	6199676406
Blue	973637810	1954901136	2812074791	469840224	1056822852	1340786550	5806685096	6199146502
Fiona	973652784	1954931510	2812129698	469847730	1056828691	1340902736	5807025162	6199437609
Karect	973650880	1954929616	2812153109	469847558	1056827837	1341529923	5807130529	6199647692
Lighter	973650027	1954932142	2812133532	469846600	1056816359	1341081880	5806729873	6198071892
Musket	973654027	1954933908	2812157389	469849138	1056829003	1341378585	5807181419	6199553951
RACER	973568663	1954896583	2811887873	469814560	1056805849	1337241860	5804649423	6193298979
SGA-EC	973653813	1954932504	2812146301	469848917	1056828854	1341281680	5807162255	6199608343
Trowel	973637311	1954932965	2812127117	469847097	1056829444	1341303372	5807194307	6199626029
				**False Positives (FP) – newly introduced errors**				
ACE	11677	8424	27799	4317	12971	1704723	2259541	1733689
BFC	530	784	2325	308	453	208911	9755	48551
Blue	23995	37162	120731	12952	8471	809236	590384	650265
Fiona	2641	3237	36199	2201	1114	697478	239447	301954
Karect	4853	5856	11865	2481	2273	46854	128678	80121
Lighter	4864	2539	30517	3084	13411	493732	525707	1653388
Musket	864	774	6650	547	765	196952	74116	171110
RACER	68975	39371	284440	27985	24868	4443967	2673149	6591309
SGA-EC	1169	2366	17850	811	1015	296150	95000	118356
Trowel	17589	1703	36902	2593	322	272190	61239	98964
				**False Negatives (FN) – remaining errors**				
ACE	3077	2683	14041	1593	1548	748107	139964	765300
BFC	10832	8426	348803	2775	2539	285163	55299	148821
Blue	3572	1715	113685	1364	503	1132600	248773	1041986
Fiona	9208	2036	42942	4406	1075	569220	112234	866386
Karect	898	1685	4283	806	740	897266	61366	294752
Lighter	61758	11026	110850	32029	20236	1483959	1000030	4384507
Musket	19988	11366	58119	7925	9564	1299912	550568	1090406
RACER	13078	2956	31690	5943	1316	916081	214034	863661
SGA-EC	37401	10771	22934352	8758	2629	1027556	203649	1349522
Trowel	1518960	628194	6938039	619671	340421	3904451	2162752	14433803

4 Assembly Result for Real Data

In order to see the impact of EC tools on assembly results, we used SPAdes, DISCOVAR, IDBA and Velvet to assemble both corrected and uncorrected data. Quast provides comprehensive information on the assembly quality. For the Quast analyses, scaffolds were used. The following commands were used to run the assemblers.

- SPAdes (v. 3.7.1)
  `spades.py -t 32 --only-assembler --12 reads.fastq -o outputDir`
- DISCOVAR
  `DiscovarDeNovo READS=reads.fastq OUT_DIR=outputDir MEMORY_CHECK=true NUM_THREADS=32`
- Velvet (v. 1.2.10)
  `./velveth asmDir 31 -fastq -shortPaired reads.fastq`
  `./velvetg asmDir -exp_cov auto -cov_cutoff auto`
- IDBA(v. 1.1.1)
  `./idba --no_correct -r reads.fa -o outputDir --num_threads 32`

Quast results for each dataset are shown in the following subsections. Assemblies were named after the EC tool that was used to preprocess the data. The 'Uncorrected' assembly was obtained from uncorrected data. Default parameter settings are used for Quast, i.e., all statistics are based on contigs of size $\geq$ 500 bp. The Quast (v. 4.4) command line was:

```
./quast.py asmDir/contigs.fa -R genome.fasta -o quastReport --plots-format ps -1 uncorrectedForwardRead.fq -2 uncorrectedReverseRead.fq --labels "toolName"
```

4.1 DISCOVAR

4.1.1 *B. dentium*

Table 6 contains the Quast report after assembling dataset *B. dentium* with DISCOVAR .

Table 6: Assembly quality metrics for *B. dentium*

Assembly	Uncorrected	ACE	BayesHammer	BFC	BLESS2	Blue	Karect	Lighter	Musket	RACER	SGA-EC
# contigs (≥ 0 bp)	41	40	34	34	28	40	31	34	37	31	34
# contigs (≥ 1000 bp)	19	22	18	18	17	23	18	18	20	18	18
# contigs (≥ 5000 bp)	17	18	16	16	15	18	16	16	18	15	16
# contigs (≥ 10000 bp)	15	16	14	14	13	16	14	14	16	13	14
# contigs (≥ 25000 bp)	15	15	14	14	13	14	14	14	15	13	14
# contigs (≥ 50000 bp)	11	10	10	10	10	10	10	10	11	10	10
Total length (≥ 0 bp)	2620312	2620550	2618818	2618819	2615877	2618828	2618035	2618817	2618219	**2907328**	2618819
Total length (≥ 1000 bp)	2613881	2614837	2613775	2613776	2612673	2613044	2613959	2613774	2612796	**2903513**	2613776
Total length (≥ 5000 bp)	2610478	2607949	2610372	2610373	2609270	2604556	2610556	2610371	2609393	**2896805**	2610373
Total length (≥ 10000 bp)	2599192	2596663	2599086	2599087	2597984	2593270	2599270	2599085	2598107	**2885519**	2599087
Total length (≥ 25000 bp)	2599192	2581604	2599086	2599087	2597984	2567611	2599270	2599085	2580596	**2885519**	2599087
Total length (≥ 50000 bp)	2440269	2386840	2440165	2440166	2480462	2408689	2440349	2440165	2421675	**2767997**	2440166
# contigs	20	23	19	19	18	25	19	19	21	19	19
Largest contig	610357	573238	610358	**610359**	608981	608981	610358	610358	534355	610358	**610359**
Total length	2614583	2615539	2614477	2614478	2613375	2614479	2614661	2614476	2613498	**2904215**	2614478
Reference length	2636367	2636367	2636367	2636367	2636367	2636367	2636367	2636367	2636367	2636367	2636367
GC (%)	58.51	58.51	58.51	58.51	58.50	58.51	58.51	58.51	58.51	58.63	58.51
Reference GC (%)	58.54	58.54	58.54	58.54	58.54	58.54	58.54	58.54	58.54	58.54	58.54
N50	288053	271664	288054	288054	288054	271663	288053	288054	288054	**397536**	288054
NG50	288053	271664	288054	288054	288054	271663	288053	288054	288054	**397536**	288054
N75	148905	158597	158597	158597	172541	158597	158597	158597	158597	**174159**	158597
NG75	148905	158597	158597	158597	172541	158597	158597	158597	158597	**190580**	158597
L50	4	4	4	4	4	4	4	4	4	**3**	4
LG50	4	4	4	4	4	4	4	4	4	**3**	4
L75	7	7	7	7	7	7	7	7	7	**6**	7
LG75	7	7	7	7	7	7	7	7	7	**5**	7
# misassemblies	**0**	**0**	**0**	**0**	1	1	**0**	**0**	1	1	**0**
# misassembled contigs	**0**	**0**	**0**	**0**	1	1	**0**	**0**	1	1	**0**
Misassembled contigs length	**0**	**0**	**0**	**0**	608981	608981	**0**	**0**	534355	578715	**0**
# local misassemblies	**1**	2	**1**	**1**	2	**1**	**1**	**1**	**1**	2	**1**
# unaligned mis. contigs	0	0	0	0	0	0	0	0	0	0	0
# unaligned contigs	0 + 0 part	0 + 0 part	0 + 0 part	0 + 0 part	0 + 0 part	0 + 0 part	0 + 0 part	0 + 0 part	0 + 0 part	0 + 0 part	0 + 0 part
Unaligned length	0	0	0	0	0	0	0	0	0	0	0
Genome fraction (%)	**99.116**	**99.116**	**99.116**	**99.116**	99.064	99.064	**99.116**	**99.116**	99.064	**99.116**	**99.116**
Duplication ratio	**1.001**	**1.001**	**1.001**	**1.001**	**1.001**	**1.001**	**1.001**	**1.001**	**1.001**	1.111	**1.001**
# N's per 100 kbp	**0.00**	3.82	**0.00**	**0.00**	**0.00**	**0.00**	**0.00**	**0.00**	**0.00**	3.44	**0.00**
# mismatches per 100 kbp	0.31	0.31	0.34	**0.23**	0.27	0.27	0.31	0.27	**0.23**	0.31	**0.23**
# indels per 100 kbp	0.31	0.38	0.31	0.34	0.31	**0.23**	0.27	0.38	0.31	0.27	0.34
Largest alignment	610357	572957	610358	**610359**	425802	425802	610358	610358	425802	610358	**610359**
Total aligned length	2614583	2615073	2614477	2614478	2613100	2614294	2614476	2614476	2613498	**2903840**	2614478
NA50	288053	271664	288054	288054	288054	271663	288053	288054	288054	**289208**	288054
NGA50	288053	271664	288054	288054	288054	271663	288053	288054	288054	**289208**	288054
NA75	148905	158597	158597	158597	172541	158597	158597	158597	148905	**174159**	158597
NGA75	148905	158597	158597	158597	172541	158597	158597	158597	148905	**190305**	158597
LA50	4	4	4	4	4	4	4	4	4	4	4
LGA50	4	4	4	4	4	4	4	4	4	4	4
LA75	**7**	**7**	**7**	**7**	8	8	**7**	**7**	8	**7**	**7**
LGA75	7	7	7	7	8	8	7	7	8	**6**	7

4.1.2 *E. coli str. K-12 substr. DH10B*

Table 7 contains the Quast report after assembling dataset *E. coli str. K-12 substr. DH10B* with DISCOVAR .

Table 7: Assembly quality metrics for *E. coli str. K-12 substr. DH10B*

Assembly	Uncorrected	ACE	BayesHammer	BFC	BLESS2	Blue	Karect	Lighter	Musket	RACER	SGA-EC
# contigs ($\geq$ 0 bp)	274	323	291	238	264	297	207	269	308	295	186
# contigs ($\geq$ 1000 bp)	79	73	74	74	71	75	73	71	74	72	75
# contigs ($\geq$ 5000 bp)	61	58	60	60	57	60	58	56	59	57	58
# contigs ($\geq$ 10000 bp)	56	53	55	54	52	53	53	51	54	51	53
# contigs ($\geq$ 25000 bp)	49	47	49	48	47	48	48	46	48	46	46
# contigs ($\geq$ 50000 bp)	31	31	31	30	31	32	30	28	30	28	29
Total length ($\geq$ 0 bp)	4592120	4872509	4554459	5034288	5148745	5301868	5248314	5234472	4721486	**5364848**	4399093
Total length ($\geq$ 1000 bp)	4531525	4797173	4487930	4982888	5091870	5233985	5207238	5173957	4651894	**5297842**	4362699
Total length ($\geq$ 5000 bp)	4487709	4759362	4451693	4945793	5055238	5196661	5166445	5135383	4613533	**5259841**	4314781
Total length ($\geq$ 10000 bp)	4449112	4716994	4409556	4896653	5012927	5139964	5124088	5093028	4571240	**5209142**	4272644
Total length ($\geq$ 25000 bp)	4337336	4624344	4317759	4804003	4935021	5062002	5046126	5015066	4478624	**5131267**	4151701
Total length ($\geq$ 50000 bp)	3690981	4050737	3670597	4157698	4350700	**4461391**	4393097	4369294	3833349	4455347	3534867
# contigs	94	88	89	89	82	88	82	84	87	88	85
Largest contig	343551	652557	411914	1074043	421583	652365	**1172452**	652461	652557	837099	326329
Total length	4542042	4807144	4497803	4993249	5099259	5242952	5213422	5183003	4660793	**5308813**	4369755
Reference length	4686137	4686137	4686137	4686137	4686137	4686137	4686137	4686137	4686137	4686137	4686137
GC (%)	50.72	50.68	50.72	50.74	50.85	50.71	50.76	50.74	50.72	50.76	50.75
Reference GC (%)	50.78	50.78	50.78	50.78	50.78	50.78	50.78	50.78	50.78	50.78	50.78
N50	97356	133298	107848	132162	158602	163361	133298	165875	122330	**171773**	117698
NG50	95058	158602	97317	156704	163311	165947	163410	173935	122330	**177622**	107823
N75	59899	62449	59899	67455	**74835**	70471	67455	**74835**	59899	**74835**	61504
NG75	57141	70655	55107	76722	88864	87147	85684	88864	59899	**112586**	54707
L50	12	11	13	10	10	10	9	**8**	11	9	12
LG50	13	10	14	8	9	9	**7**	**7**	11	**7**	14
L75	26	24	26	23	23	24	22	**20**	25	**20**	25
LG75	28	22	29	20	19	18	17	16	25	**15**	29
# misassemblies	3	5	4	5	8	8	6	7	**2**	11	3
# misassembled contigs	3	5	4	3	8	8	4	7	**2**	8	3
Misassembled contigs length	634863	1149960	755823	1310461	1749325	1874510	1759738	2375577	774887	2222845	**401102**
# local misassemblies	14	3	6	**2**	3	8	5	**2**	3	9	9
# structural variations	0	0	0	0	0	0	0	0	0	0	0
# unaligned mis. contigs	0	0	0	0	0	0	0	0	0	0	0
# unaligned contigs	0 + 0 part	0 + 0 part	0 + 0 part	0 + 0 part	0 + 0 part	0 + 0 part	0 + 0 part	0 + 0 part	0 + 0 part	0 + 0 part	0 + 0 part
Unaligned length	0	0	0	0	0	0	0	0	0	0	0
Genome fraction (%)	92.370	92.439	**93.254**	92.496	92.422	92.584	92.419	92.452	92.424	92.655	92.391
Duplication ratio	1.049	1.110	1.029	1.152	1.177	1.208	1.204	1.196	1.076	1.223	**1.009**
# N's per 100 kbp	41.83	14.56	22.23	12.02	25.49	24.80	21.10	19.29	**10.73**	22.60	27.46
# mismatches per 100 kbp	1.85	1.02	1.21	0.62	**0.42**	1.01	1.34	0.65	1.06	0.92	1.62
# indels per 100 kbp	0.14	0.05	0.05	**0.02**	0.07	0.05	0.12	0.07	0.05	**0.02**	0.16
Largest alignment	**326329**	326328	326328	326129	326319	326232	326129	326328	326328	326322	**326329**
Total aligned length	4540136	4806444	4496802	4992649	5097903	5241579	5212322	5182003	4660277	**5307613**	4368499
NA50	95058	112586	89397	117698	117685	117698	**132162**	131963	117698	131963	107823
NGA50	92505	117698	88680	132162	133298	132162	**156703**	**156703**	117698	133298	97317
NA75	57141	59800	56402	62449	62250	62449	**67455**	**67455**	59799	62449	56567
NGA75	54607	67455	54261	76722	82808	83023	**85684**	82987	59799	82958	45709
LA50	14	13	14	13	14	14	13	13	**12**	14	13
LGA50	15	12	15	12	12	12	**11**	**11**	12	12	14
LA75	29	27	29	27	29	29	27	27	**26**	29	27
LGA75	31	25	32	24	24	23	**22**	**22**	26	23	32

4.1.3 *E. coli str. K-12 substr. MG1655*

Table 8 contains the Quast report after assembling dataset *E. coli str. K-12 substr. MG1655* with DISCOVAR .

Table 8: Assembly quality metrics for *E. coli str. K-12 substr. MG1655*

Assembly	Uncorrected	ACE	BayesHammer	BFC	BLESS2	Blue	Karect	Lighter	Musket	RACER	SGA-EC
# contigs (≥ 0 bp)	136	205	1168	207	215	304	243	215	311	208	271
# contigs (≥ 1000 bp)	124	77	608	105	90	92	82	95	87	88	207
# contigs (≥ 5000 bp)	3	61	247	74	67	67	61	71	64	69	3
# contigs (≥ 10000 bp)	0	54	133	69	57	59	55	63	54	62	0
# contigs (≥ 25000 bp)	0	46	30	54	47	45	46	52	44	46	0
# contigs (≥ 50000 bp)	0	30	4	35	31	31	32	37	30	31	0
Total length (≥ 0 bp)	236904	4583890	4673678	4581495	4582720	4681774	4816991	4583866	**5001900**	4587380	398754
Total length (≥ 1000 bp)	233516	4547305	4511593	4549381	4542301	4625105	4767154	4548075	**4935390**	4554309	380114
Total length (≥ 5000 bp)	21752	4503937	3550363	4463067	4482054	4556845	4716578	4490654	**4877839**	4502287	24634
Total length (≥ 10000 bp)	0	4450193	2720691	4428272	4401991	4499387	4668891	4427303	**4798997**	4448767	0
Total length (≥ 25000 bp)	0	4316532	1134734	4194714	4221717	4276337	4525945	4245050	**4628383**	4182324	0
Total length (≥ 50000 bp)	0	3749345	256307	3471755	3672133	3780324	4057531	3703361	**4138053**	3644641	0
# contigs	124	82	624	111	102	99	96	103	99	94	210
Largest contig	9147	365228	73863	221402	238045	429293	471577	211687	418937	**480612**	9536
Total length	233516	4551290	4522598	4554385	4551550	4629907	4777246	4553997	**4944315**	4558950	382165
Reference length	4639675	4639675	4639675	4639675	4639675	4639675	4639675	4639675	4639675	4639675	4639675
GC (%)	39.56	50.73	50.70	50.74	50.74	50.69	50.72	50.72	50.67	50.73	41.64
Reference GC (%)	50.79	50.79	50.79	50.79	50.79	50.79	50.79	50.79	50.79	50.79	50.79
N50	1725	130621	13296	88143	112270	117964	112492	95324	**134676**	105110	1743
NG50	-	125346	12916	87072	106364	117964	117885	92024	164309	105110	-
N75	1423	59654	5929	57478	58977	59666	**67528**	58857	66071	**67528**	1396
NG75	-	58850	5266	49405	58105	59666	78892	57312	87166	61545	-
L50	44	12	94	18	13	12	12	17	**11**	15	74
LG50	-	13	99	19	14	12	11	18	10	15	-
L75	80	25	218	35	27	26	25	32	**23**	27	135
LG75	-	26	234	36	28	26	23	33	20	28	-
# misassemblies	**0**	9	1	9	6	13	7	5	10	16	**0**
# misassembled contigs	**0**	7	1	7	6	6	7	5	9	12	**0**
Misassembled contigs length	**0**	1135500	62521	494731	942233	1089006	1516331	510573	1791746	1387624	**0**
# local misassemblies	**0**	13	24	17	18	16	6	11	9	5	**0**
# unaligned mis. contigs	0	0	0	0	0	0	0	0	0	0	0
# unaligned contigs	0 + 0 part	0 + 0 part	0 + 0 part	0 + 0 part	0 + 0 part	0 + 0 part	0 + 0 part	0 + 0 part	0 + 0 part	0 + 0 part	0 + 0 part
Unaligned length	0	0	0	0	0	0	0	0	0	0	0
Genome fraction (%)	5.015	97.960	95.944	97.913	97.916	97.741	**98.317**	97.985	98.207	98.097	8.205
Duplication ratio	1.004	**1.001**	1.016	1.003	1.002	1.021	1.047	1.002	1.085	1.002	1.004
# N's per 100 kbp	**0.00**	68.11	159.20	103.20	105.46	88.55	31.40	59.29	50.56	48.26	**0.00**
# mismatches per 100 kbp	3.01	0.95	1.98	0.64	**0.18**	0.31	0.31	0.46	0.50	1.74	3.68
# indels per 100 kbp	**0.00**	0.35	1.10	0.59	0.57	0.37	0.13	0.31	0.15	0.24	**0.00**
Largest alignment	9147	268205	73663	221199	237845	209037	**268448**	209311	209518	209273	9536
Total aligned length	233516	4547442	4514693	4548941	4546078	4625272	4775037	4550674	**4941146**	4555709	382165
NA50	1725	110240	13241	79327	95454	95121	**112492**	91824	112450	86536	1743
NGA50	-	105571	12901	78999	95454	95121	112492	87309	132756	86536	-
NA75	1423	58208	5855	45590	57827	56387	**59588**	57312	58666	48231	1396
NGA75	-	58094	5259	43901	57311	56387	63364	56944	63620	44021	-
LA50	44	**14**	96	19	16	17	**14**	18	15	19	74
LGA50	-	15	100	20	16	17	14	19	14	19	-
LA75	80	**29**	220	38	31	34	**29**	34	30	36	135
LGA75	-	30	236	39	32	34	27	35	27	38	-

4.1.4 *S. enterica*

Table 9 contains the Quast report after assembling dataset *S. enterica* with DISCOVAR .

Table 9: Assembly quality metrics for *S. enterica*

Assembly	Uncorrected	ACE	BayesHammer	BFC	BLESS2	Blue	Karect	Lighter	Musket	RACER	SGA-EC
# contigs (≥ 0 bp)	57	58	60	53	53	96	49	60	64	57	60
# contigs (≥ 1000 bp)	26	26	25	24	29	26	24	26	26	25	27
# contigs (≥ 5000 bp)	21	21	20	20	26	21	20	21	21	20	23
# contigs (≥ 10000 bp)	20	20	19	18	24	20	18	20	20	20	21
# contigs (≥ 25000 bp)	19	19	18	17	22	19	17	19	19	19	20
# contigs (≥ 50000 bp)	17	17	17	16	21	18	16	17	18	18	18
Total length (≥ 0 bp)	8163172	6749101	5393710	7876026	8740562	6725492	7983640	5267128	7609062	**8827553**	7139332
Total length (≥ 1000 bp)	8152999	6737889	5382222	7866252	8732154	6704366	7974783	5255839	7596472	**8815758**	7127974
Total length (≥ 5000 bp)	8138322	6723525	5367858	7854968	8723916	6689707	7963123	5241077	7581909	**8801195**	7116576
Total length (≥ 10000 bp)	8132122	6717325	5361658	7842409	8711266	6683507	7950564	5234877	7575709	**8801195**	7104017
Total length (≥ 25000 bp)	8121235	6706944	5351283	7832038	8669276	6673137	7939669	5224509	7565341	**8780558**	7093636
Total length (≥ 50000 bp)	8037149	6623285	5313751	7794506	8631738	6635805	7902137	5140850	7527809	**8741247**	7009977
# contigs	27	29	27	25	30	28	26	28	28	29	30
Largest contig	**2045651**	**2045651**	1022875	**2045651**	1059914	1319742	**2045651**	1022875	**2045651**	2045552	1227131
Total length	8153995	6740191	5383886	7867248	8733150	6705863	7976367	5257503	7598186	**8818658**	7130310
Reference length	4888768	4888768	4888768	4888768	4888768	4888768	4888768	4888768	4888768	4888768	4888768
GC (%)	52.02	51.95	52.18	52.08	52.10	52.18	52.06	52.08	52.00	52.12	51.99
Reference GC (%)	52.09	52.09	52.09	52.09	52.09	52.09	52.09	52.09	52.09	52.09	52.09
N50	**1128667**	564383	471517	879542	556744	597403	879542	439239	879542	890143	492948
NG50	1227131	614042	471517	1128568	899660	879165	1128568	439239	1128568	**1319841**	1060013
N75	407470	321307	298751	439351	407470	323519	439318	231546	323519	**597104**	321307
NG75	**1128667**	564383	321307	879542	763376	597403	879542	298751	879542	900602	492948
L50	**3**	4	4	**3**	6	4	**3**	4	**3**	4	4
LG50	**2**	**2**	4	**2**	3	3	**2**	4	**2**	**2**	3
L75	7	8	8	7	10	7	7	9	7	**6**	8
LG75	**3**	4	7	**3**	4	4	**3**	7	**3**	**3**	4
# misassemblies	32	26	**18**	27	46	26	31	19	29	36	27
# misassembled contigs	11	10	10	11	20	11	11	**9**	11	12	11
Misassembled contigs length	7380321	5730649	4495114	7369278	8485367	5978777	7476855	**4086405**	6776877	8144235	6117395
# local misassemblies	6	9	**3**	6	7	6	7	4	6	8	6
# unaligned mis. contigs	0	0	0	0	0	0	0	0	0	0	0
# unaligned contigs	3 + 4 part	3 + 4 part	3 + 4 part	3 + 4 part	3 + 4 part	3 + 4 part	3 + 4 part	3 + 4 part	3 + 4 part	3 + 4 part	3 + 4 part
Unaligned length	85006	84608	**60978**	66482	91260	82781	88086	82781	84807	85007	88086
Genome fraction (%)	96.588	96.583	96.589	96.590	90.498	96.560	**96.605**	96.584	96.588	95.827	96.580
Duplication ratio	1.709	1.410	1.127	1.652	1.953	1.403	1.670	**1.096**	1.591	1.864	1.492
# N's per 100 kbp	13.49	14.84	**7.43**	13.98	38.93	13.42	16.30	9.51	13.16	19.28	14.02
# mismatches per 100 kbp	7.71	7.94	8.03	7.69	**6.83**	8.09	8.91	7.98	7.54	8.03	7.48
# indels per 100 kbp	0.47	**0.36**	0.38	0.40	0.38	0.38	1.06	**0.36**	**0.36**	0.38	0.49
Largest alignment	**453219**	**453219**	**453219**	**453219**	383351	**453219**	**453219**	**453219**	**453219**	**453219**	385791
Total aligned length	8068080	6654783	5322508	7799766	8638780	6622282	7887181	5174322	7512575	**8732343**	7041424
NA50	**231546**	216238	216238	231347	175570	215208	231347	215202	**231546**	216238	215202
NGA50	**349181**	339455	231546	348982	297351	271281	339455	216238	339455	339455	339455
NA75	140342	134245	106253	**161809**	109398	117146	161610	106253	161610	140241	133818
NGA75	**235808**	215202	161809	231546	195759	180274	231546	134245	231546	235198	215202
LA50	12	10	**9**	12	17	11	12	**9**	11	14	12
LGA50	**7**	**7**	8	**7**	8	**7**	**7**	8	**7**	**7**	**7**
LA75	23	20	**17**	22	32	21	23	**17**	21	26	22
LGA75	**10**	12	14	11	13	13	11	15	11	11	12

4.1.5 *P. aeruginosa*

Table 10 contains the Quast report after assembling dataset *P. aeruginosa* with DISCOVAR .

Table 10: Assembly quality metrics for *P. aeruginosa*

Assembly	Uncorrected	ACE	BayesHammer	BFC	BLESS2	Blue	Karect	Lighter	Musket	RACER	SGA-EC
# contigs ($\geq$ 0 bp)	235	257	214	237	285	155	248	175	208	235	198
# contigs ($\geq$ 1000 bp)	100	58	63	51	98	46	59	70	56	66	78
# contigs ($\geq$ 5000 bp)	86	43	49	39	79	36	46	53	46	50	64
# contigs ($\geq$ 10000 bp)	86	43	47	39	79	35	46	53	46	50	63
# contigs ($\geq$ 25000 bp)	75	40	46	36	69	33	43	48	42	44	58
# contigs ($\geq$ 50000 bp)	60	39	44	34	58	32	42	45	41	42	51
Total length ($\geq$ 0 bp)	11625729	11634936	10503702	10420741	**12042989**	11140745	11989806	11767482	11524362	11873579	10961929
Total length ($\geq$ 1000 bp)	11582521	11575379	10456654	10365266	**11986568**	11108207	11932686	11734935	11477897	11822085	10924088
Total length ($\geq$ 5000 bp)	11551657	11542556	10428297	10338168	**11935032**	11084356	11903187	11700878	11453202	11787070	10891866
Total length ($\geq$ 10000 bp)	11551657	11542556	10417440	10338168	**11935032**	11077115	11903187	11700878	11453202	11787070	10886855
Total length ($\geq$ 25000 bp)	11383796	11509234	10406408	10304846	11770514	11054584	**11869865**	11644056	11394722	11704610	10800207
Total length ($\geq$ 50000 bp)	10849911	11477737	10343168	10230546	11371155	11022244	**11838368**	11541634	11363225	11636822	10559422
# contigs	112	74	77	66	113	53	74	81	71	79	89
Largest contig	555743	950726	758235	1712382	1099014	**1712467**	1101178	1099225	1101178	1101178	727357
Total length	11590443	11585582	10465902	10374758	**11996711**	11113273	11942534	11742174	11487346	11830623	10931407
Reference length	6264404	6264404	6264404	6264404	6264404	6264404	6264404	6264404	6264404	6264404	6264404
GC (%)	66.55	66.58	66.53	66.58	66.58	66.57	66.55	66.59	66.61	66.55	66.56
Reference GC (%)	66.56	66.56	66.56	66.56	66.56	66.56	66.56	66.56	66.56	66.56	66.56
N50	226123	475847	336413	509581	233095	436693	457755	354411	365189	**578979**	253019
NG50	353311	651197	488647	760385	655399	**776261**	580931	580931	697483	727189	457723
N75	127449	231991	188258	232707	133440	**238833**	230657	232839	233095	221660	137433
NG75	257909	582785	364869	574308	356909	651944	526453	376883	595827	**656305**	329577
L50	18	9	11	**7**	13	**7**	10	11	9	8	13
LG50	8	4	5	**3**	4	**3**	4	4	4	4	6
L75	34	18	21	**15**	29	**15**	19	21	18	18	27
LG75	13	7	9	6	8	**5**	7	8	6	6	10
# misassemblies	88	45	51	44	83	**43**	51	54	50	54	62
# misassembled contigs	85	43	47	39	79	**35**	46	52	45	50	62
Misassembled contigs length	11373850	11542556	10417440	**10338168**	11935032	11077115	11903187	11583348	11333515	11787070	10864735
# local misassemblies	9	7	**2**	**2**	11	16	4	4	3	**2**	6
# unaligned mis. contigs	0	0	0	0	0	0	0	0	0	0	0
# unaligned contigs	0 + 1 part	0 + 1 part	0 + 1 part	0 + 1 part	0 + 1 part	0 + 1 part	0 + 1 part	0 + 1 part	0 + 1 part	0 + 1 part	**0 + 0 part**
Unaligned length	20158	20258	20258	20258	20258	20358	20258	20258	20258	20258	**0**
Genome fraction (%)	94.090	92.618	83.725	82.911	**96.082**	88.807	95.430	94.785	92.714	94.584	87.741
Duplication ratio	**1.963**	1.993	1.992	1.994	1.990	1.994	1.994	1.974	1.974	1.993	1.989
# N's per 100 kbp	74.20	39.70	45.86	38.56	78.35	**35.09**	42.70	44.28	40.91	43.95	55.80
# mismatches per 100 kbp	0.78	**0.59**	0.93	0.83	0.88	1.73	0.85	0.74	1.31	0.71	0.73
# indels per 100 kbp	0.64	0.72	0.78	**0.62**	0.83	0.88	0.84	0.66	0.77	0.74	0.80
Largest alignment	277921	475263	379167	856091	549407	**856283**	550788	549662	550788	550788	363728
Total aligned length	11561645	11560824	10440921	10350599	**11967149**	11089016	11917271	11716808	11461927	11805252	10925274
NA50	112912	237774	168256	188488	115225	188311	228927	177255	182644	**289340**	126360
NGA50	177807	325449	244174	**380043**	277543	349328	290316	290316	348592	363445	228712
NA75	61958	112823	91289	111071	63883	**115483**	110730	100361	109533	100505	68567
NGA75	130009	291243	182283	254840	178305	218396	263077	188491	297764	**328003**	164639
LA50	35	18	21	**13**	26	16	19	21	17	16	26
LGA50	15	8	10	**6**	9	**6**	8	8	8	8	12
LA75	68	36	42	**30**	61	34	39	42	36	36	54
LGA75	25	14	18	**11**	16	12	14	15	12	12	20

4.1.6 *H. sapiens* Chr. 21

Table 11 contains the Quast report after assembling dataset *H. sapiens* Chr. 21 with DISCOVAR .

Table 11: Assembly quality metrics for *H. sapiens* Chr. 21

Assembly	Uncorrected	ACE	BayesHammer	BFC	BLESS2	Blue	Karect	Lighter	Musket	RACER	SGA-EC
# contigs (≥ 0 bp)	27142	28951	27677	30461	27602	31994	29444	28813	28902	29266	28622
# contigs (≥ 1000 bp)	8724	8872	8615	8922	8659	8846	8956	8854	8903	8966	8830
# contigs (≥ 5000 bp)	291	300	300	358	295	385	313	308	311	313	301
# contigs (≥ 10000 bp)	45	51	45	72	50	85	52	54	50	55	55
# contigs (≥ 25000 bp)	2	2	3	5	4	4	3	3	3	2	3
# contigs (≥ 50000 bp)	0	0	0	0	0	0	0	0	0	0	0
Total length (≥ 0 bp)	23408496	24205535	23398741	25197489	23563324	**25978783**	24713094	24238862	24388697	24638523	24099519
Total length (≥ 1000 bp)	18534837	18873907	18350432	19502701	18544529	**19856763**	19251190	18939574	19071438	19245204	18855664
Total length (≥ 5000 bp)	2236239	2390389	2345949	2943213	2358076	**3238607**	2509559	2460569	2447363	2507608	2409899
Total length (≥ 10000 bp)	657387	772745	689726	1107992	789211	**1277133**	814402	824225	769572	846768	824665
Total length (≥ 25000 bp)	58348	59509	84445	**154711**	119213	121978	85547	85543	84420	59716	85544
Total length (≥ 50000 bp)	0	0	0	0	0	0	0	0	0	0	0
# contigs	8925	9108	8816	9142	8844	9092	9209	9079	9132	9197	9037
Largest contig	30142	31299	30202	**36962**	32650	33532	31299	31300	30195	31488	31299
Total length	18682551	19045980	18496737	19660516	18683473	**20033594**	19441662	19105744	19242695	19415777	19007561
Reference length	46709983	46709983	46709983	46709983	46709983	46709983	46709983	46709983	46709983	46709983	46709983
GC (%)	39.74	39.97	39.70	40.18	39.65	40.44	40.00	39.95	40.00	40.00	39.91
Reference GC (%)	42.22	42.22	42.22	42.22	42.22	42.22	42.22	42.22	42.22	42.22	42.22
N50	2151	2158	2166	2227	2184	**2314**	2187	2173	2179	2182	2171
N75	1533	1522	1529	1548	1537	**1576**	1538	1529	1536	1537	1532
L50	2674	2686	2610	2611	2615	**2517**	2701	2669	2689	2695	2665
L75	5266	5335	5178	5282	5186	**5171**	5374	5312	5343	5370	5299
# misassemblies	7	**5**	**5**	6	**5**	**5**	**5**	7	**5**	10	8
# misassembled contigs	7	**5**	**5**	6	**5**	**5**	**5**	7	**5**	10	8
Misassembled contigs length	11595	**7184**	7866	21301	7237	32262	16937	22039	19282	36377	35449
# local misassemblies	39	40	**33**	60	47	56	42	35	40	52	36
# structural variations	0	0	0	0	0	0	0	0	0	0	0
# unaligned mis. contigs	**0**	**0**	**0**	**0**	**0**	**0**	1	**0**	**0**	**0**	**0**
# unaligned contigs	11 + 1 part	9 + 2 part	5 + 1 part	8 + 3 part	5 + 4 part	**4 + 2 part**	9 + 4 part	11 + 3 part	7 + 2 part	7 + 4 part	7 + 2 part
Unaligned length	13200	12644	6862	12574	10467	**5235**	14528	14858	12580	12369	11621
Genome fraction (%)	39.758	40.515	39.397	41.844	39.795	**42.658**	41.365	40.658	40.951	41.310	40.459
Duplication ratio	**1.005**	1.006	**1.005**	**1.005**	**1.005**	**1.005**	**1.005**	**1.005**	**1.005**	1.006	**1.005**
# N's per 100 kbp	**11.24**	13.13	14.06	21.36	15.52	22.46	13.89	13.61	12.99	15.97	13.68
# mismatches per 100 kbp	86.72	86.79	86.70	88.20	86.67	88.13	87.61	87.28	87.45	89.43	**86.12**
# indels per 100 kbp	18.62	18.87	**18.26**	19.58	18.36	18.56	19.36	18.86	18.86	19.26	18.82
Largest alignment	29939	31199	30002	**36761**	32348	33332	31199	31200	30095	31388	31199
Total aligned length	18662994	19026207	18481892	19637168	18665168	**20017474**	19419142	19083052	19222660	19393898	18988684
NA50	2149	2156	2165	2225	2183	**2313**	2186	2172	2178	2180	2169
NGA50	-	-	-	-	-	-	-	-	-	-	-
NA75	1531	1520	1528	1544	1533	**1574**	1536	1526	1533	1533	1529
LA50	2676	2688	2612	2615	2617	**2522**	2704	2672	2692	2700	2669
LA75	5270	5339	5182	5290	5190	**5179**	5379	5319	5349	5378	5305

4.1.7 *C. elegans*

Table 12 contains the Quast report after assembling dataset *C. elegans* with DISCOVAR .

Table 12: Assembly quality metrics for *C. elegans*

Assembly	Uncorrected	ACE	BayesHammer	BFC	BLESS2	Blue	Karect	Lighter	Musket	RACER	SGA-EC
# contigs (≥ 0 bp)	33879	35629	34072	34338	34851	36053	35285	34300	34770	37198	34010
# contigs (≥ 1000 bp)	27381	27222	27337	26996	27904	26458	27217	27363	27294	27887	27244
# contigs (≥ 5000 bp)	3874	3685	3893	4101	3041	4098	4012	3840	3723	3391	3989
# contigs (≥ 10000 bp)	584	588	616	689	411	768	672	595	574	497	648
# contigs (≥ 25000 bp)	23	28	25	33	17	61	31	24	24	22	28
# contigs (≥ 50000 bp)	1	1	1	3	3	6	2	1	1	1	1
Total length (≥ 0 bp)	88711959	88080769	89019134	90477027	83674565	**90945967**	90382710	88648128	87898196	87242922	89720536
Total length (≥ 1000 bp)	86109765	84805863	86321966	**87577530**	80876971	87415714	87218441	85875614	84947836	83429141	87003150
Total length (≥ 5000 bp)	30047522	28818579	30349677	32512747	23082132	**33990281**	31726003	29867219	28965455	26124208	31301586
Total length (≥ 10000 bp)	8115815	8324546	8544789	9742241	5873440	**11724956**	9576884	8347025	8049310	7028244	9090758
Total length (≥ 25000 bp)	716932	885165	797767	1060134	668624	**2171500**	1089069	780103	766392	699825	898189
Total length (≥ 50000 bp)	54399	57601	54393	164608	248925	**471404**	174936	54399	54399	57601	54399
# contigs	28936	29090	28958	28685	29587	28212	29020	29012	29008	30336	28871
Largest contig	54399	57601	54393	57890	108677	**115103**	**115103**	54399	54399	57601	54399
Total length	87209529	86131218	87468860	**88775004**	82072189	88662798	88498583	87045920	86166399	85171657	88159881
Reference length	100286070	100286070	100286070	100286070	100286070	100286070	100286070	100286070	100286070	100286070	100286070
GC (%)	38.03	38.41	38.06	38.27	38.35	38.35	38.13	38.18	38.27	38.34	38.12
Reference GC (%)	35.44	35.44	35.44	35.44	35.44	35.44	35.44	35.44	35.44	35.44	35.44
N50	3687	3628	3707	3860	3283	**3935**	3777	3668	3635	3430	3775
NG50	3192	3086	3213	3388	2669	**3429**	3310	3165	3097	2904	3289
N75	2238	2202	2241	2296	2047	**2311**	2265	2225	2204	2094	2265
NG75	1707	1641	1719	**1809**	1416	1808	1775	1695	1642	1536	1765
L50	7038	7047	7009	6811	7521	**6433**	6900	7038	7050	7400	6936
LG50	8944	9165	8866	8405	10603	**8017**	8566	8981	9159	9797	8658
L75	14674	14720	14642	14312	15484	**13839**	14510	14704	14716	15387	14523
LG75	19700	20305	19539	18540	23504	**18096**	18916	19820	20284	21714	19071
# misassemblies	**113**	141	114	130	121	141	149	117	120	915	116
# misassembled contigs	**105**	137	108	124	119	138	145	112	116	894	111
Misassembled contigs length	965372	1074547	889920	1049107	989929	1155666	1240877	912972	**882752**	2312963	958991
# local misassemblies	90	92	96	89	93	**79**	87	86	85	118	91
# unaligned mis. contigs	3	**2**	**2**	5	3	**2**	3	**2**	**2**	**2**	3
# unaligned contigs	3929 + 37 part	3966 + 36 part	3895 + 34 part	3636 + 38 part	4009 + 33 part	**3130 + 34 part**	3936 + 38 part	3935 + 37 part	3948 + 35 part	3988 + 43 part	3879 + 36 part
Unaligned length	10018419	10738295	10217534	11068366	**9903052**	11424350	10691877	10453916	10459102	10525274	10518396
Genome fraction (%)	76.482	74.634	76.543	76.947	71.452	76.381	**76.965**	75.919	75.037	73.737	76.931
Duplication ratio	**1.006**	1.007	**1.006**	1.007	1.007	1.008	1.008	**1.006**	**1.006**	1.009	**1.006**
# N's per 100 kbp	8.14	9.98	8.35	10.03	10.23	13.65	11.30	8.16	**7.89**	8.34	9.53
# mismatches per 100 kbp	**4.25**	8.39	4.38	4.60	4.87	5.48	5.24	13.21	15.35	14.20	**4.25**
# indels per 100 kbp	2.87	3.10	**2.61**	2.83	2.63	3.07	3.01	3.09	3.19	4.08	2.91
Largest alignment	**27606**	25320	25354	**27606**	25315	**27606**	**27606**	**27606**	25347	25347	**27606**
Total aligned length	77165313	75351336	77225216	77677096	72141274	77206533	**77775255**	76561502	75676690	74548243	77615090
NA50	3269	3124	3257	3254	2886	3208	3278	3203	3158	2955	**3284**
NGA50	2786	2613	2785	2830	2303	2777	**2837**	2715	2643	2433	2834
NA75	**1818**	1742	1809	1788	1681	1753	1800	1787	1766	1656	1810
NGA75	1274	633	1276	**1301**	-	1266	**1301**	1236	994	-	1298
LA50	**7909**	8188	7952	8052	8538	8097	7970	8039	8094	8586	7941
LGA50	10073	10664	10077	9946	12076	10042	**9903**	10282	10535	11408	9928
LA75	**16849**	17423	16957	17253	17870	17439	17081	17152	17226	18240	16977
LGA75	23261	24993	23256	22895	-	23267	**22838**	23799	24516	-	22887

4.1.8 *D. melanogaster*

Table 13 contains the Quast report after assembling dataset *D. melanogaster* with DISCOVAR .

Table 13: Assembly quality metrics for *D. melanogaster*

Assembly	Uncorrected	ACE	BayesHammer	BFC	BLESS2	Blue	Karect	Lighter	Musket	RACER	SGA-EC
# contigs (≥ 0 bp)	27260	30244	29569	27393	28469	21402	25185	28576	24938	26720	28174
# contigs (≥ 1000 bp)	12634	14793	14325	12449	21034	10019	13299	13371	13790	15317	13061
# contigs (≥ 5000 bp)	4940	5340	5242	4864	5765	4093	4965	5093	5172	5268	5046
# contigs (≥ 10000 bp)	2986	3026	2979	2946	2574	2690	2968	3023	3022	2934	2970
# contigs (≥ 25000 bp)	1136	1030	1065	1165	515	1256	1166	1125	1099	1024	1150
# contigs (≥ 50000 bp)	364	242	286	378	54	504	323	318	273	243	328
Total length (≥ 0 bp)	122045333	122366187	122006358	**122808874**	114602943	121557134	121827634	122473174	120004005	120942507	122466576
Total length (≥ 1000 bp)	117164542	117232929	116938531	117847785	112114552	117816277	**117894949**	117394289	116463039	117195927	117387138
Total length (≥ 5000 bp)	97048788	92303485	92940657	97807043	71943394	**102159157**	95701726	95560675	93443597	90424200	96364085
Total length (≥ 10000 bp)	83408784	76258640	77232839	84501065	50210781	**92385558**	81803352	81166940	78545771	74170057	81868999
Total length (≥ 25000 bp)	53624596	44485080	46772910	55830709	18559399	**68976793**	52576273	50764105	47763708	43698411	52402012
Total length (≥ 50000 bp)	26851850	17448614	19919080	28437865	3330371	**42383546**	23590856	22668748	19399922	16853382	23901384
# contigs	13973	16228	15743	13841	21908	11072	14427	14800	14729	16459	14508
Largest contig	212522	159060	187339	225726	91562	**368590**	198380	198532	171899	198165	181461
Total length	118112708	118247885	117934833	**118830170**	112727088	118562819	118699015	118403026	117124660	118001344	118409815
Reference length	120381546	120381546	120381546	120381546	120381546	120381546	120381546	120381546	120381546	120381546	120381546
GC (%)	42.44	42.49	42.44	42.45	42.65	42.48	42.44	42.45	42.53	42.50	42.45
Reference GC (%)	42.42	42.42	42.42	42.42	42.42	42.42	42.42	42.42	42.42	42.42	42.42
N50	22091	17377	18249	22788	8075	**33082**	21345	20330	19153	16735	21287
NG50	21451	16988	17561	22303	7119	**32109**	20764	19863	18358	16199	20838
N75	7864	5805	6121	7934	3744	**12284**	7079	6997	6524	5360	7320
NG75	7184	5389	5567	7502	3214	**11308**	6643	6479	5776	5016	6759
L50	1368	1739	1642	1316	3263	**919**	1460	1500	1593	1775	1448
LG50	1420	1801	1710	1350	3769	**947**	1500	1550	1680	1847	1495
L75	3573	4664	4421	3464	8671	**2377**	3827	3935	4182	4894	3779
LG75	3800	4949	4736	3614	10327	**2493**	4011	4156	4580	5241	3989
# misassemblies	991	1017	999	1021	1092	**983**	1016	993	1011	1250	988
# misassembled contigs	903	932	914	923	1033	**867**	927	901	927	1132	900
Misassembled contigs length	20252411	15883432	15918142	19834439	**9381705**	27249040	18786258	17767341	16750517	19442661	18461236
# local misassemblies	1907	1879	1896	1840	**1763**	1835	1811	1838	1883	1821	1852
# unaligned mis. contigs	109	111	105	122	**65**	82	100	110	84	100	109
# unaligned contigs	1269 + 844 part	1303 + 859 part	1310 + 840 part	1283 + 861 part	1308 + 624 part	1290 + 691 part	1257 + 879 part	1272 + 848 part	1314 + 756 part	1393 + 869 part	**1250 + 867 part**
Unaligned length	5979319	5979218	6019433	6131439	**4567032**	5380727	6035967	6027963	5434932	5734625	6056066
Genome fraction (%)	92.002	91.859	91.758	92.304	88.732	**92.553**	92.363	92.076	91.677	91.737	92.023
Duplication ratio	**1.012**	1.015	1.013	1.014	1.013	1.016	1.013	1.014	**1.012**	1.017	1.014
# N's per 100 kbp	205.23	212.86	213.51	215.52	**170.23**	200.74	241.03	210.13	224.80	224.40	201.84
# mismatches per 100 kbp	543.91	546.01	543.28	545.31	**542.17**	545.48	547.20	545.75	551.17	548.97	544.36
# indels per 100 kbp	129.04	128.27	128.22	129.21	**123.93**	129.62	130.06	129.19	128.88	127.95	129.12
Largest alignment	175992	158169	152405	152426	84393	**278506**	181494	178460	171298	162199	154126
Total aligned length	111608713	111717063	111375299	112144165	107696193	**112649626**	112079362	111833973	111127562	111640564	111830509
NA50	19250	15446	16182	19817	7324	**27349**	18525	17833	16821	14528	18818
NGA50	18832	15025	15582	19469	6537	**26744**	18121	17358	16161	14081	18357
NA75	6427	4927	5068	6460	3427	**9819**	5724	5697	5462	4737	6010
NGA75	5852	4679	4766	6051	2881	**9058**	5285	5223	4915	4461	5485
LA50	1570	1953	1842	1510	3582	**1113**	1669	1703	1803	2064	1638
LGA50	1629	2023	1919	1550	4135	**1146**	1715	1759	1902	2147	1691
LA75	4143	5365	5096	4043	9439	**2862**	4465	4559	4816	5716	4378
LGA75	4420	5698	5469	4229	11261	**3007**	4694	4831	5290	6104	4635

4.2 IDBA

4.2.1 *B. dentium*

Table 14 contains the Quast report after assembling dataset *B. dentium* with IDBA .

Table 14: Assembly quality metrics for *B. dentium*

Assembly	Uncorrected	ACE	BayesHammer	BFC	BLESS2	Blue	Fiona	Karect	Lighter	Musket	RACER	SGA-EC	Trowel
# contigs (≥ 0 bp)	5027	2032	4055	3632	2220	3338	3103	3945	3423	3001	2523	4845	4177
# contigs (≥ 1000 bp)	154	99	109	82	57	70	83	74	97	88	80	162	140
# contigs (≥ 5000 bp)	112	76	81	58	42	51	63	55	71	66	60	115	101
# contigs (≥ 10000 bp)	85	64	69	53	39	48	53	49	58	58	55	91	79
# contigs (≥ 25000 bp)	31	34	37	38	31	32	36	31	38	36	35	36	40
# contigs (≥ 50000 bp)	9	17	16	21	18	19	18	19	16	15	18	5	11
Total length (≥ 0 bp)	**3587351**	2819442	3496247	3480758	3117137	3444880	3414588	3476847	3453335	3417411	3367147	3568629	3518137
Total length (≥ 1000 bp)	2609222	2604126	2611819	2614744	2607722	2615696	2610144	2613192	2614549	2614679	**2615893**	2606824	2612925
Total length (≥ 5000 bp)	2514677	2553828	2553978	2567890	2580180	**2582139**	2571400	2581371	2560121	2572371	2576281	2499243	2533087
Total length (≥ 10000 bp)	2315068	2469354	2466567	2532364	2556678	**2560520**	2504335	2533329	2459554	2509701	2541682	2317366	2380329
Total length (≥ 25000 bp)	1438392	1953661	1957421	2283949	**2433293**	2273090	2233421	2217746	2110486	2125279	2211135	1418973	1758731
Total length (≥ 50000 bp)	589275	1303207	1203707	1649327	**1965790**	1810343	1562706	1780649	1310233	1354916	1577255	363499	748566
# contigs	352	117	296	268	125	252	259	260	284	276	262	357	327
Largest contig	99811	157035	119824	174678	197580	197580	210973	**211639**	147538	197572	174086	103372	149572
Total length	**2731975**	2615596	2727231	2729908	2647585	2726849	2717295	2728488	2729152	2730793	2727323	2727106	2728098
Reference length	2636367	2636367	2636367	2636367	2636367	2636367	2636367	2636367	2636367	2636367	2636367	2636367	2636367
GC (%)	58.64	58.49	58.63	58.63	58.51	58.64	58.61	58.64	58.63	58.64	58.63	58.64	58.63
Reference GC (%)	58.54	58.54	58.54	58.54	58.54	58.54	58.54	58.54	58.54	58.54	58.54	58.54	58.54
N50	30669	47845	44252	58654	**98980**	71013	55343	64662	47671	48847	62439	25330	31841
NG50	31451	47845	45395	60136	**98980**	73724	56050	64662	47845	58654	62741	25469	32380
N75	14834	24492	21701	35593	**47845**	35678	34935	34935	29210	27070	32388	15784	16984
NG75	15684	24492	23506	37464	**47845**	39437	35678	42204	29913	29824	36267	16326	19214
L50	29	18	20	16	**10**	12	15	12	18	16	15	34	27
LG50	27	18	19	15	**10**	11	14	12	17	15	14	33	26
L75	64	35	41	31	**19**	25	30	26	36	34	30	70	55
LG75	59	35	38	29	**19**	23	28	24	34	31	28	66	51
# misassemblies	0	0	0	0	0	0	0	0	0	0	0	0	0
# misassembled contigs	0	0	0	0	0	0	0	0	0	0	0	0	0
Misassembled contigs length	0	0	0	0	0	0	0	0	0	0	0	0	0
# local misassemblies	2	2	2	2	2	2	2	2	2	2	2	2	2
# unaligned mis. contigs	0	0	0	0	0	0	0	0	0	0	0	0	0
# unaligned contigs	194 + 0 part	**12 + 0 part**	189 + 0 part	188 + 0 part	66 + 0 part	186 + 0 part	174 + 0 part	186 + 0 part	187 + 0 part	190 + 0 part	185 + 0 part	192 + 0 part	188 + 0 part
Unaligned length	124776	**6960**	122785	121410	40083	118938	109568	120541	119583	122203	118530	123616	121155
Genome fraction (%)	98.738	98.815	98.701	98.844	98.847	98.848	98.812	98.845	98.852	98.834	**98.863**	98.649	98.746
Duplication ratio	1.002	**1.001**	**1.001**	**1.001**	**1.001**	**1.001**	**1.001**	**1.001**	**1.001**	**1.001**	**1.001**	**1.001**	**1.001**
# N's per 100 kbp	0.00	0.00	0.00	0.00	0.00	0.00	0.00	0.00	0.00	0.00	0.00	0.00	0.00
# mismatches per 100 kbp	**0.23**	**0.23**	**0.23**	**0.23**	**0.23**	0.35	0.27	**0.23**	0.27	0.31	**0.23**	**0.23**	**0.23**
# indels per 100 kbp	0.27	**0.23**	0.27	0.31	0.58	**0.23**	0.27	0.31	0.31	0.27	**0.23**	0.27	0.27
Largest alignment	99811	156760	119824	174678	197580	197580	210973	**211639**	147538	197572	174086	103372	149572
Total aligned length	2606924	2608361	2604171	2608223	2607227	2607636	2607452	2607672	**2609294**	2608315	2608518	2603215	2606668
NA50	30669	47845	44252	58654	**98980**	71013	55343	64662	47671	48847	62439	25330	31841
NGA50	31451	47845	45395	60136	**98980**	73724	56050	64662	47845	58654	62741	25469	32380
NA75	14834	24492	21701	35593	**47845**	35678	34935	34935	29210	27070	32388	15784	16984
NGA75	15684	24492	23506	37189	**47845**	39437	35678	42204	29913	29824	36267	16326	18972
LA50	29	18	20	16	**10**	12	15	12	18	16	15	34	27
LGA50	27	18	19	15	**10**	11	14	12	17	15	14	33	26
LA75	64	35	41	31	**19**	25	30	26	36	34	30	70	55
LGA75	59	35	38	29	**19**	23	28	24	34	31	28	66	51

4.2.2 *E. coli str. K-12 substr. DH10B*

Table 15 contains the Quast report after assembling dataset *E. coli str. K-12 substr. DH10B* with IDBA .

Table 15: Assembly quality metrics for *E. coli str. K-12 substr. DH10B*

Assembly	Uncorrected	ACE	BayesHammer	BFC	BLESS2	Blue	Fiona	Karect	Lighter	Musket	RACER	SGA-EC	Trowel
# contigs (≥ 0 bp)	2360	1011	974	641	614	752	704	845	681	674	667	1735	1154
# contigs (≥ 1000 bp)	125	121	122	110	107	115	112	110	112	112	114	167	127
# contigs (≥ 5000 bp)	97	95	97	86	85	90	89	85	88	88	89	130	101
# contigs (≥ 10000 bp)	85	79	82	76	73	77	77	74	75	74	76	106	89
# contigs (≥ 25000 bp)	63	60	61	57	56	59	58	55	57	58	56	63	64
# contigs (≥ 50000 bp)	29	32	33	33	32	31	31	32	33	31	32	25	30
Total length (≥ 0 bp)	**4515653**	4411601	4409433	4386875	4383754	4393892	4391199	4400623	4390421	4389610	4387773	4462636	4424457
Total length (≥ 1000 bp)	4329090	4327143	4328658	**4329413**	4327908	4328587	4327815	4328681	4328305	4328052	4328697	4326431	4329230
Total length (≥ 5000 bp)	4265809	4275841	4276153	4281299	**4284862**	4280221	4280926	4278797	4280020	4279741	4279445	4240734	4270897
Total length (≥ 10000 bp)	4173490	4150608	4159568	**4201688**	4190257	4176438	4185157	4189340	4176318	4166508	4175910	4060828	4178528
Total length (≥ 25000 bp)	3825722	3839346	3813633	3902949	3912933	3894188	3887474	3876118	3891890	**3917393**	3859576	3335440	3762545
Total length (≥ 50000 bp)	2633615	2854610	2791981	3072002	**3082420**	2893257	2935976	3070569	3044346	2971324	3026965	1953025	2503554
# contigs	133	129	131	119	115	124	121	118	122	121	122	176	135
Largest contig	239163	269573	206204	**269702**	**269702**	177084	269660	269637	**269702**	**269702**	269699	174732	178824
Total length	4334631	4333487	4335683	**4336423**	4334181	4335568	4334707	4335004	4336308	4335381	4334729	4332925	4335159
Reference length	4686137	4686137	4686137	4686137	4686137	4686137	4686137	4686137	4686137	4686137	4686137	4686137	4686137
GC (%)	50.75	50.74	50.75	50.75	50.75	50.74	50.75	50.75	50.75	50.74	50.75	50.75	50.75
Reference GC (%)	50.78	50.78	50.78	50.78	50.78	50.78	50.78	50.78	50.78	50.78	50.78	50.78	50.78
N50	60090	61037	62810	**77601**	**77601**	76358	76364	77573	73020	**77601**	76339	45611	58765
NG50	58765	58765	59786	66088	67274	63502	66087	**67318**	63521	66088	67228	43033	55566
N75	34689	40132	41267	**43033**	43015	41478	40710	43011	42648	41772	41772	28506	35180
NG75	30831	31552	31676	**34059**	34044	34058	32622	34058	34058	32648	31675	21579	30973
L50	21	20	23	**18**	**18**	19	19	**18**	19	**18**	19	30	24
LG50	24	23	26	21	**20**	22	21	**20**	22	21	21	34	28
L75	44	41	44	37	**36**	40	39	**36**	38	38	37	60	48
LG75	53	49	51	44	**43**	46	46	**43**	45	45	45	71	56
# misassemblies	**0**	**0**	1	1	2	1	1	2	1	**0**	1	1	**0**
# misassembled contigs	**0**	**0**	1	1	1	1	1	1	1	**0**	1	1	**0**
Misassembled contigs length	**0**	**0**	73043	50204	160773	115259	115095	160695	73020	**0**	73061	57259	**0**
# local misassemblies	**0**	1	**0**	2	2	**0**	1	1	**0**	**0**	**0**	**0**	**0**
# structural variations	0	0	0	0	0	0	0	0	0	0	0	0	0
# unaligned mis. contigs	0	0	0	0	0	0	0	0	0	0	0	0	0
# unaligned contigs	**0 + 0 part**	**0 + 0 part**	**0 + 0 part**	**0 + 0 part**	**0 + 0 part**	**0 + 0 part**	1 + 0 part	**0 + 0 part**	**0 + 0 part**	**0 + 0 part**	**0 + 0 part**	**0 + 0 part**	**0 + 0 part**
Unaligned length	**0**	**0**	**0**	**0**	**0**	**0**	777	**0**	**0**	**0**	**0**	**0**	**0**
Genome fraction (%)	92.524	92.538	92.551	92.557	92.510	92.549	92.514	92.541	**92.558**	92.536	92.527	92.493	92.539
Duplication ratio	1.000	**0.999**	1.000	1.000	1.000	1.000	1.000	1.000	1.000	1.000	1.000	1.000	1.000
# N's per 100 kbp	**0.00**	**0.00**	**0.00**	**0.00**	1.32	**0.00**	**0.00**	**0.00**	**0.00**	**0.00**	**0.00**	**0.00**	**0.00**
# mismatches per 100 kbp	**0.16**	0.88	0.67	0.92	0.95	0.95	1.01	0.85	1.01	1.18	2.01	0.83	0.48
# indels per 100 kbp	1.75	4.34	2.56	1.78	**1.41**	1.94	2.05	2.35	2.01	1.68	2.01	2.24	2.31
Largest alignment	239163	269573	206204	**269702**	**269702**	177084	269660	269637	**269702**	**269702**	269699	174732	178824
Total aligned length	4334630	4333450	4335683	**4336421**	4334122	4335559	4333913	4335004	4336306	4335379	4334723	4332925	4334971
NA50	60090	61037	61004	**77601**	**77601**	76358	76364	77573	67318	**77601**	76339	45611	58765
NGA50	58765	58765	59634	**66088**	66087	63502	66087	66063	62810	**66088**	66087	43033	55566
NA75	34689	40132	40148	**43033**	43015	41478	40710	41772	41731	41772	41267	27454	35180
NGA75	30831	31552	31552	**34059**	34044	32647	32622	34058	32818	32648	31675	21281	30973
LA50	21	20	23	**18**	**18**	19	19	**18**	19	**18**	19	30	24
LGA50	24	23	26	**21**	**21**	22	**21**	**21**	22	**21**	**21**	34	28
LA75	44	41	45	**37**	**37**	40	39	38	39	38	38	61	48
LGA75	53	49	52	**44**	**44**	47	46	**44**	46	45	45	72	56

4.2.3 *E. coli str. K-12 substr. MG1655*

Table 16 contains the Quast report after assembling dataset *E. coli str. K-12 substr. MG1655* with IDBA .

Table 16: Assembly quality metrics for *E. coli str. K-12 substr. MG1655*

Assembly	Uncorrected	ACE	BayesHammer	BFC	BLESS2	Blue	Fiona	Karect	Lighter	Musket	RACER	SGA-EC	Trowel
# contigs ($\geq$ 0 bp)	2764	2174	803	1082	810	1210	1352	936	1220	1176	1332	2229	1314
# contigs ($\geq$ 1000 bp)	116	129	99	115	110	118	117	110	114	121	120	116	116
# contigs ($\geq$ 5000 bp)	93	100	75	88	81	90	87	84	88	92	93	91	91
# contigs ($\geq$ 10000 bp)	80	88	70	78	73	77	77	74	77	81	85	79	78
# contigs ($\geq$ 25000 bp)	58	61	54	56	54	57	57	54	56	59	58	58	57
# contigs ($\geq$ 50000 bp)	33	36	33	33	33	34	33	34	34	34	34	33	34
Total length ($\geq$ 0 bp)	**4747542**	4690218	4605109	4619712	4599723	4625294	4633230	4609966	4630594	4624123	4635398	4706232	4641206
Total length ($\geq$ 1000 bp)	4525935	4527292	**4532359**	4531014	4531064	4529382	4530010	4529620	4530595	4530717	4529205	4526427	4531535
Total length ($\geq$ 5000 bp)	4472536	4458330	4472750	4470195	4462677	4464111	4460408	4469372	4468887	4461488	4464995	4465235	**4477643**
Total length ($\geq$ 10000 bp)	4373336	4373951	**4435795**	4394143	4400650	4363962	4386938	4392757	4386462	4379290	4406336	4373714	4379980
Total length ($\geq$ 25000 bp)	4037922	3955590	**4213354**	4077735	4113882	4052339	4087233	4100329	4072451	4058027	4017375	4050236	4056754
Total length ($\geq$ 50000 bp)	3136081	3037009	**3436733**	3234735	3355993	3217140	3218457	3382460	3272272	3148687	3147332	3159213	3235091
# contigs	129	138	110	126	119	129	128	123	124	132	132	129	127
Largest contig	209341	173958	**221477**	209350	221408	209346	209346	209344	173964	173963	173962	209302	209349
Total length	4535920	4534370	**4540813**	4539316	4537947	4537626	4538026	4539311	4538316	4538868	4538199	4535889	4540024
Reference length	4639675	4639675	4639675	4639675	4639675	4639675	4639675	4639675	4639675	4639675	4639675	4639675	4639675
GC (%)	50.74	50.73	50.74	50.74	50.74	50.73	50.73	50.74	50.73	50.74	50.74	50.73	50.74
Reference GC (%)	50.79	50.79	50.79	50.79	50.79	50.79	50.79	50.79	50.79	50.79	50.79	50.79	50.79
N50	82762	66115	**95664**	86971	95437	80468	82764	86968	82761	80468	82031	82766	82762
NG50	80467	65113	**95664**	82764	86972	78592	82764	86968	82550	78592	80378	80469	82552
N75	42704	41736	**50620**	44004	48769	43791	43792	48769	45417	43177	42696	41954	43792
NG75	41267	41031	**48769**	42697	45437	42509	42700	45437	43217	41718	41267	41019	41780
L50	19	23	**17**	18	**17**	19	19	18	19	20	20	19	19
LG50	20	24	**17**	19	18	20	19	18	20	21	21	20	20
L75	39	44	**33**	37	34	39	38	35	37	40	40	39	38
LG75	41	46	**34**	39	36	40	39	37	39	42	42	41	40
# misassemblies	**0**	**0**	1	1	**0**	**0**	**0**	1	**0**	**0**	2	**0**	**0**
# misassembled contigs	**0**	**0**	1	1	**0**	**0**	**0**	1	**0**	**0**	2	**0**	**0**
Misassembled contigs length	**0**	**0**	10212	10203	**0**	**0**	**0**	10153	**0**	**0**	104717	**0**	**0**
# local misassemblies	**1**	2	12	**1**	7	**1**	2	2	2	**1**	2	**1**	**1**
# unaligned mis. contigs	0	0	0	0	0	0	0	0	0	0	0	0	0
# unaligned contigs	3 + 0 part	2 + 0 part	4 + 0 part	3 + 0 part	**0 + 1 part**	3 + 0 part	3 + 0 part	3 + 0 part	3 + 0 part	3 + 0 part	3 + 0 part	3 + 0 part	3 + 0 part
Unaligned length	2325	1336	3165	2325	**1232**	2325	2275	2328	2321	2325	2326	2325	2325
Genome fraction (%)	97.695	97.691	97.771	97.767	97.756	97.735	97.743	97.770	97.748	97.751	97.741	97.692	**97.777**
Duplication ratio	1.000	1.000	1.000	1.000	1.000	1.000	1.000	1.000	1.000	1.000	1.000	1.000	1.000
# N's per 100 kbp	0.44	0.55	14.12	0.29	5.02	0.46	0.37	0.24	0.64	0.11	**0.09**	0.42	0.22
# mismatches per 100 kbp	0.22	1.08	0.73	0.73	0.97	0.53	1.68	1.41	0.60	1.01	1.17	**0.20**	0.46
# indels per 100 kbp	1.76	4.70	1.76	1.59	**1.50**	1.98	1.81	1.85	1.74	1.92	2.40	1.77	1.90
Largest alignment	209341	173958	**221392**	209350	221360	209346	209346	209344	173964	173963	173962	209302	209349
Total aligned length	4533238	4532587	4536848	4536542	4536266	4535022	4535432	4536704	4535629	4536019	4535447	4533187	**4537044**
NA50	82762	66115	**95658**	86971	95394	80468	82764	86968	82761	80468	77721	82766	82762
NGA50	80467	65113	**95658**	82764	86972	78592	82764	86968	82550	78592	77721	80469	82552
NA75	42704	41727	**50620**	44004	48769	43791	43792	48769	45342	43175	41869	41954	43792
NGA75	41267	41031	**48769**	42697	45437	42509	42700	45437	43217	41718	41267	41019	41780
LA50	19	23	**17**	18	**17**	19	19	18	19	20	21	19	19
LGA50	20	24	**17**	19	18	20	19	18	20	21	21	20	20
LA75	39	44	**33**	37	34	39	38	35	37	40	41	39	38
LGA75	41	46	**34**	39	36	40	39	37	39	42	43	41	40

4.2.4 *S. enterica*

Table 17 contains the Quast report after assembling dataset *S. enterica* with IDBA .

Table 17: Assembly quality metrics for *S. enterica*

Assembly	Uncorrected	ACE	BayesHammer	BFC	BLESS2	Blue	Fiona	Karect	Lighter	Musket	RACER	SGA-EC	Trowel
# contigs (≥ 0 bp)	812	823	869	491	443	558	506	479	600	686	572	565	563
# contigs (≥ 1000 bp)	79	77	70	71	67	71	73	69	75	73	71	78	79
# contigs (≥ 5000 bp)	66	65	62	63	58	62	62	60	66	63	60	66	66
# contigs (≥ 10000 bp)	61	58	59	57	54	55	57	55	60	59	55	61	61
# contigs (≥ 25000 bp)	51	50	49	48	45	48	48	47	50	50	46	50	49
# contigs (≥ 50000 bp)	40	37	39	39	35	37	38	37	38	40	36	39	40
Total length (≥ 0 bp)	**4821474**	4804536	4806987	4790253	4783105	4792819	4790169	4786252	4798745	4801080	4793989	4798672	4799077
Total length (≥ 1000 bp)	4725334	4722054	4725977	4725529	**4729204**	4724999	4725076	4726267	4723966	4723639	4726072	4726664	4727836
Total length (≥ 5000 bp)	4700100	4695972	4706517	4701537	**4708332**	4705864	4701692	4705593	4703476	4701522	4700545	4702472	4698906
Total length (≥ 10000 bp)	4661202	4644548	**4685098**	4656215	4674900	4653437	4662492	4669155	4658992	4672133	4663193	4663624	4663395
Total length (≥ 25000 bp)	4513568	4509580	4521938	4515340	4539096	**4550383**	4527714	4546144	4512460	4537287	4510828	4495372	4461605
Total length (≥ 50000 bp)	4076548	4009703	4141396	4164954	4156507	4129418	4146808	**4165139**	4040894	4157010	4129115	4085924	4115384
# contigs	89	92	83	87	79	84	87	87	85	88	87	91	89
Largest contig	238647	250669	251312	233440	**459069**	270864	271383	271408	251213	270980	251397	300827	251469
Total length	4731590	4731287	4733912	4734797	4736663	4732933	4733552	**4737085**	4730461	4733123	4735739	4734531	4734203
Reference length	4888768	4888768	4888768	4888768	4888768	4888768	4888768	4888768	4888768	4888768	4888768	4888768	4888768
GC (%)	52.12	52.12	52.12	52.12	52.12	52.12	52.12	52.12	52.12	52.12	52.12	52.12	52.12
Reference GC (%)	52.09	52.09	52.09	52.09	52.09	52.09	52.09	52.09	52.09	52.09	52.09	52.09	52.09
N50	95967	103744	103707	104432	105442	105471	104212	105364	103730	103041	**113432**	96275	96157
NG50	95967	96604	103707	103449	104432	104432	103730	104151	103730	96499	**105573**	96162	95970
N75	60821	73757	73753	76273	77767	76975	76712	**77948**	59513	73751	77944	60821	60821
NG75	58498	72340	59289	60614	**76975**	73711	60821	**76975**	58498	60821	73752	58498	58498
L50	17	16	16	15	**13**	15	15	15	16	16	14	16	16
LG50	17	17	16	16	**14**	16	16	16	16	17	15	17	17
L75	31	30	29	29	**26**	28	28	28	30	30	27	30	30
LG75	33	31	31	31	**27**	30	30	29	32	32	29	32	32
# misassemblies	12	12	12	**11**	12	12	12	12	12	12	12	12	12
# misassembled contigs	11	11	11	**10**	11	11	11	11	11	11	11	11	11
Misassembled contigs length	1033227	948436	1034172	**943010**	1263534	1032332	1031396	1032285	991683	1031666	1032946	1014811	1033117
# local misassemblies	67	57	53	63	**43**	53	62	51	68	63	53	61	56
# unaligned mis. contigs	0	0	0	0	0	0	0	0	0	0	0	0	0
# unaligned contigs	5 + 5 part	4 + 5 part	**3 + 5 part**	6 + 6 part	**3 + 5 part**	5 + 5 part	5 + 5 part	6 + 5 part	4 + 5 part	5 + 5 part	5 + 5 part	5 + 5 part	5 + 5 part
Unaligned length	61435	61416	**60729**	62794	61137	62242	61957	62807	61625	62237	62200	61715	61855
Genome fraction (%)	95.501	95.466	95.571	95.548	95.592	95.521	95.502	**95.595**	95.481	95.525	95.579	95.552	95.546
Duplication ratio	**1.000**	1.001	**1.000**	**1.000**	**1.000**	**1.000**	1.001	**1.000**	**1.000**	**1.000**	**1.000**	**1.000**	**1.000**
# N's per 100 kbp	0.00	0.00	0.00	0.00	0.00	0.00	0.00	0.00	0.00	0.00	0.00	0.00	0.00
# mismatches per 100 kbp	7.52	6.49	7.34	6.34	8.56	6.51	8.57	6.33	7.50	6.55	6.53	7.43	**6.29**
# indels per 100 kbp	0.47	0.28	0.49	0.36	0.75	0.30	0.69	0.28	0.54	**0.26**	0.30	0.49	0.28
Largest alignment	238647	250669	251312	233440	283927	270864	271383	271408	251213	270980	251397	**300827**	251469
Total aligned length	4669316	4667770	4672698	4671545	4673500	4670212	4669307	**4673820**	4668360	4670394	4673052	4671906	4671511
NA50	82209	93265	90110	90108	**96263**	93583	89837	95926	89730	89868	93347	86748	86993
NGA50	79980	93265	86993	86993	**93583**	89628	89837	**93583**	86993	86852	93347	82085	82103
NA75	55119	56400	56890	56890	**58734**	56707	56601	58724	55119	56662	56890	55119	55119
NGA75	52699	53069	53855	55119	**56890**	56354	55119	**56890**	53069	55119	55119	53069	53069
LA50	19	18	17	17	**15**	16	17	16	17	18	16	18	18
LGA50	20	18	18	18	**16**	17	17	17	18	19	**16**	19	19
LA75	36	34	34	34	**30**	33	33	32	35	35	32	35	36
LGA75	39	36	37	36	**32**	35	35	34	37	37	34	37	38

4.2.5 *P. aeruginosa*

Table 18 contains the Quast report after assembling dataset *P. aeruginosa* with IDBA .

Table 18: Assembly quality metrics for *P. aeruginosa*

Assembly	Uncorrected	ACE	BayesHammer	BFC	BLESS2	Blue	Fiona	Karect	Lighter	Musket	RACER	SGA-EC	Trowel
# contigs (≥ 0 bp)	640	1130	634	443	445	575	501	633	494	600	842	558	576
# contigs (≥ 1000 bp)	99	130	108	106	109	115	110	115	109	108	110	102	108
# contigs (≥ 5000 bp)	74	103	81	78	77	84	80	86	81	81	80	75	81
# contigs (≥ 10000 bp)	68	94	76	73	73	79	74	79	76	76	74	71	77
# contigs (≥ 25000 bp)	59	76	64	63	63	67	64	66	64	66	62	60	65
# contigs (≥ 50000 bp)	38	41	39	40	39	40	40	40	39	40	39	38	39
Total length (≥ 0 bp)	6239779	**6253244**	6234266	6224805	6223542	6231832	6227762	6231337	6227904	6233154	6245761	6232323	6233959
Total length (≥ 1000 bp)	6174563	6152014	6169579	**6175263**	6174246	6170609	6171734	6166898	6173390	6170887	6169227	6170833	6170970
Total length (≥ 5000 bp)	**6113582**	6088350	6105032	6108520	6101292	6102324	6102432	6099653	6107461	6105881	6100101	6107264	6106618
Total length (≥ 10000 bp)	6076163	6027150	6072784	6076383	6074339	6070208	6064233	6051239	6075891	6073767	6062610	**6084914**	6084289
Total length (≥ 25000 bp)	**5923485**	5709368	5869363	5909336	5908572	5858988	5898372	5805141	5870750	5907954	5857882	5902501	5878244
Total length (≥ 50000 bp)	**5112838**	4369494	4949439	5058315	5020886	4874531	5007305	4838181	4945028	4965029	5027585	5087636	4912645
# contigs	114	148	124	123	126	132	126	132	125	126	125	119	125
Largest contig	326181	250136	275768	326189	326267	326202	325991	325840	326196	**326346**	326048	326183	326193
Total length	6184663	6163897	6180295	**6186722**	6185705	6181504	6182450	6178149	6184137	6183023	6179265	6182323	6182463
Reference length	6264404	6264404	6264404	6264404	6264404	6264404	6264404	6264404	6264404	6264404	6264404	6264404	6264404
GC (%)	66.59	66.59	66.59	66.59	66.59	66.59	66.59	66.59	66.59	66.59	66.59	66.59	66.59
Reference GC (%)	66.56	66.56	66.56	66.56	66.56	66.56	66.56	66.56	66.56	66.56	66.56	66.56	66.56
N50	**146539**	94524	128842	116297	112489	112485	112476	109839	112487	112487	116297	146417	112480
NG50	**146539**	94524	128842	112478	112489	112485	111578	109839	112487	112487	116297	146417	112480
N75	**71679**	47785	63533	67237	63639	58292	63360	58397	63700	63532	63700	68180	59169
NG75	**67253**	47710	59080	63700	63533	55195	59240	55255	63318	59079	63533	67245	58397
L50	**15**	23	16	16	16	17	16	18	17	17	16	**15**	17
LG50	**15**	23	16	17	16	17	17	18	17	17	16	**15**	17
L75	**30**	47	34	33	33	36	34	37	34	35	33	31	35
LG75	**31**	48	35	34	34	37	35	38	35	36	34	32	36
# misassemblies	2	**1**	**1**	**1**	**1**	**1**	**1**	**1**	2	**1**	**1**	2	2
# misassembled contigs	2	**1**	**1**	**1**	**1**	**1**	**1**	**1**	2	**1**	**1**	2	2
Misassembled contigs length	160470	**115647**	116061	116297	116297	116297	116154	116186	160337	116297	116297	160337	160337
# local misassemblies	53	171	78	47	**44**	52	65	100	55	62	75	59	60
# unaligned mis. contigs	0	0	0	0	0	0	0	0	0	0	0	0	0
# unaligned contigs	0 + 1 part	0 + 1 part	0 + 1 part	0 + 1 part	0 + 1 part	0 + 1 part	0 + 1 part	0 + 1 part	0 + 1 part	0 + 1 part	0 + 1 part	0 + 1 part	0 + 1 part
Unaligned length	10079	10079	10079	10079	10079	10079	10079	10079	10079	10079	10079	10079	10079
Genome fraction (%)	98.548	98.209	98.477	**98.580**	98.570	98.494	98.512	98.441	98.539	98.517	98.459	98.507	98.509
Duplication ratio	1.000	1.000	1.000	1.000	1.000	1.000	1.000	1.000	1.000	1.000	1.000	1.000	1.000
# N's per 100 kbp	0.00	0.00	0.00	0.00	0.00	0.00	0.00	0.00	0.00	0.00	0.00	0.00	0.00
# mismatches per 100 kbp	1.34	1.56	1.64	**1.26**	1.39	1.56	1.39	1.30	1.43	1.51	1.39	1.33	1.59
# indels per 100 kbp	0.73	0.75	0.71	0.71	1.51	**0.70**	0.76	0.79	0.78	**0.70**	0.75	0.71	0.71
Largest alignment	326126	250136	275767	326188	326267	326157	325973	325821	326054	**326346**	326048	326128	326138
Total aligned length	6174529	6153680	6170206	**6176642**	6175542	6171363	6172352	6168051	6173914	6172792	6169085	6172189	6172328
NA50	**146539**	93281	128842	112478	111603	110973	111578	103345	111389	111395	112464	146417	110996
NGA50	**146539**	93281	128842	111591	111603	110973	109872	101701	111389	111395	112464	146417	110996
NA75	**66126**	47674	59080	63700	63533	55195	59240	55255	63318	59079	63700	**66126**	59169
NGA75	**65205**	47653	58397	63533	59240	54953	58397	54953	59240	58256	63533	63700	58397
LA50	**15**	23	16	16	16	17	16	18	17	17	16	**15**	17
LGA50	**15**	23	16	17	16	17	17	19	17	17	16	**15**	17
LA75	**31**	48	35	34	34	37	35	38	35	36	33	32	35
LGA75	**32**	49	36	35	35	38	36	39	36	37	34	33	36

4.2.6 *H. sapiens* Chr. 21

Table 19 contains the Quast report after assembling dataset *H. sapiens* Chr. 21 with IDBA .

Table 19: Assembly quality metrics for *H. sapiens* Chr. 21

Assembly	Uncorrected	ACE	BayesHammer	BFC	BLESS2	Blue	Fiona	Karect	Lighter	Musket	RACER	SGA-EC	Trowel
# contigs ($\geq$ 0 bp)	102309	138880	94922	105611	91455	134746	122964	108526	107836	112054	123551	105099	110982
# contigs ($\geq$ 1000 bp)	4098	4449	3534	4123	4049	4664	4251	3925	4119	4178	4693	4100	4343
# contigs ($\geq$ 5000 bp)	2034	2045	1951	2040	2005	2065	2057	2019	2034	2047	2098	2026	2054
# contigs ($\geq$ 10000 bp)	1016	990	1056	1025	1020	945	1001	1040	1034	1026	967	1036	985
# contigs ($\geq$ 25000 bp)	188	157	243	181	187	143	178	194	186	177	132	187	165
# contigs ($\geq$ 50000 bp)	13	4	22	11	14	6	8	15	12	8	2	10	10
Total length ($\geq$ 0 bp)	39562976	**41770782**	39121526	39740631	38469011	41232601	40747821	39951470	39932723	40122382	40677687	39778296	39988114
Total length ($\geq$ 1000 bp)	31826082	31561819	**31942713**	31768142	31597457	31429606	31687664	31831542	31799582	31755892	31449067	31821983	31669381
Total length ($\geq$ 5000 bp)	26458220	25306302	**27837942**	26343661	26237295	24743806	25962836	26857358	26371881	26231497	24731102	26427931	25757309
Total length ($\geq$ 10000 bp)	19170911	17745297	**21343647**	19066792	19172151	16764356	18430354	19863425	19237728	18935031	16662665	19340614	18113654
Total length ($\geq$ 25000 bp)	6517940	5250017	**8675262**	6214022	6368441	4743003	6056407	6908248	6445975	6072489	4278541	6430367	5568310
Total length ($\geq$ 50000 bp)	761378	256413	**1300609**	633021	817430	356391	456768	891449	707687	469569	129147	604189	583509
# contigs	4726	5146	3993	4755	4593	5367	4883	4526	4742	4801	5458	4722	5043
Largest contig	69952	78911	**83462**	66569	83445	69936	66569	80638	68396	69938	69524	69950	66579
Total length	**32278872**	32061659	32275141	32224901	31991512	31940973	32141287	32264069	32248737	32202968	32001416	32272288	32172503
Reference length	46709983	46709983	46709983	46709983	46709983	46709983	46709983	46709983	46709983	46709983	46709983	46709983	46709983
GC (%)	40.61	40.57	40.60	40.59	40.52	40.53	40.58	40.60	40.60	40.60	40.55	40.60	40.57
Reference GC (%)	42.22	42.22	42.22	42.22	42.22	42.22	42.22	42.22	42.22	42.22	42.22	42.22	42.22
N50	12581	11303	**14993**	12387	12711	10627	11882	13052	12534	12237	10400	12646	11664
NG50	7039	6210	**8395**	7017	6960	5817	6564	7422	6984	6885	5803	7103	6470
N75	6478	5751	**7869**	6377	6538	5444	6124	6785	6423	6331	5409	6481	6021
L50	746	829	**632**	759	737	868	785	715	756	769	903	750	798
LG50	1513	1695	**1274**	1534	1518	1808	1604	1445	1525	1556	1843	1510	1634
L75	1640	1810	**1379**	1656	1612	1914	1722	1564	1649	1677	1959	1635	1758
# misassemblies	51	60	52	77	76	**44**	49	57	68	56	106	66	81
# misassembled contigs	48	58	49	74	73	**42**	47	55	65	54	100	63	79
Misassembled contigs length	659650	594475	800208	1074952	899972	**472393**	669011	815815	936482	707009	1290025	1022192	963237
# local misassemblies	76	67	109	73	91	68	77	78	76	72	69	76	**66**
# structural variations	0	1	0	0	0	1	0	0	1	0	0	0	0
# unaligned mis. contigs	2	3	**0**	**0**	1	**0**	**0**	2	1	**0**	**0**	2	**0**
# unaligned contigs	106 + 22 part	123 + 17 part	102 + 25 part	116 + 19 part	**84 + 28 part**	116 + 27 part	115 + 20 part	108 + 23 part	109 + 19 part	118 + 17 part	118 + 20 part	124 + 24 part	104 + 24 part
Unaligned length	143652	162260	142194	156455	**125693**	181182	145244	150318	138623	156759	154912	166104	138168
Genome fraction (%)	**68.680**	68.153	68.565	68.527	68.004	67.876	68.382	68.637	68.622	68.492	68.027	68.615	68.448
Duplication ratio	**1.002**	**1.002**	1.003	**1.002**	1.003	**1.002**	**1.002**	**1.002**	**1.002**	**1.002**	**1.002**	**1.002**	**1.002**
# N's per 100 kbp	384.89	408.14	605.02	396.11	568.92	434.75	410.71	411.02	390.94	394.41	**370.62**	397.72	395.88
# mismatches per 100 kbp	136.06	139.06	135.48	137.33	143.15	136.86	136.10	**133.75**	137.70	138.98	147.99	135.92	137.26
# indels per 100 kbp	54.77	55.70	56.20	55.33	55.66	56.15	55.37	55.33	55.54	54.97	56.07	55.09	**54.62**
Largest alignment	69952	78850	**83373**	66487	83356	69936	66486	80577	68230	69938	63517	69950	66496
Total aligned length	**32088467**	31842867	32029480	32016988	31768444	31709911	31947406	32064852	32062277	31999522	31788360	32057890	31980829
NA50	12444	11114	**14723**	12151	12322	10516	11679	12915	12315	12013	10157	12388	11431
NGA50	6948	6106	**8199**	6804	6788	5755	6478	7278	6843	6720	5635	6949	6360
NA75	6384	5651	**7677**	6250	6390	5384	6029	6662	6336	6238	5273	6370	5890
LA50	751	836	**641**	771	752	875	793	722	764	777	923	761	810
LGA50	1529	1717	**1298**	1563	1553	1823	1623	1463	1546	1577	1887	1536	1660
LA75	1657	1835	**1406**	1687	1650	1931	1743	1584	1673	1700	2005	1664	1786

4.2.7 *C. elegans*

Table 20 contains the Quast report after assembling dataset *C. elegans* with IDBA .

Table 20: Assembly quality metrics for *C. elegans*

Assembly	Uncorrected	ACE	BayesHammer	BFC	BLESS2	Blue	Fiona	Karect	Lighter	Musket	RACER	SGA-EC	Trowel
# contigs ($\geq$ 0 bp)	203415	241287	191757	198118	205987	222398	219816	199468	212134	220202	242493	204134	202600
# contigs ($\geq$ 1000 bp)	23820	27679	23229	23760	28064	23956	25503	23696	25433	26169	28621	23808	23779
# contigs ($\geq$ 5000 bp)	5265	3822	5305	5311	3471	5287	5014	5378	5078	4789	3898	5288	5254
# contigs ($\geq$ 10000 bp)	1637	741	1712	1656	647	1613	1296	1684	1329	1170	789	1651	1644
# contigs ($\geq$ 25000 bp)	198	69	209	191	59	175	125	184	118	117	90	191	195
# contigs ($\geq$ 50000 bp)	45	9	46	39	5	34	24	37	25	27	16	42	45
Total length ($\geq$ 0 bp)	118799992	114170074	117537858	118214042	109961204	**119444609**	118285707	118206096	118387916	118072643	118233003	118714248	118615565
Total length ($\geq$ 1000 bp)	**96127803**	85118518	95937053	96044197	82706061	95032072	93009114	96009464	93691897	92334252	87446335	96076605	95935042
Total length ($\geq$ 5000 bp)	53843135	32228125	**55090132**	53980078	28994075	52574207	46636697	54131678	47273617	44165284	33627639	53830727	53633067
Total length ($\geq$ 10000 bp)	28768855	11599807	**30181631**	28686276	10153864	27119766	21103125	28524948	21543242	19282752	12875429	28667755	28646245
Total length ($\geq$ 25000 bp)	8569575	2562224	**8944599**	8082901	2063112	6905329	5001497	7424989	4707794	4885306	3436135	8148320	8344784
Total length ($\geq$ 50000 bp)	3442593	559485	**3548962**	3017440	283191	2283060	1670084	2574568	1568823	1916567	1002804	3236938	3446748
# contigs	33262	41130	32320	33118	42334	33807	36584	32950	36368	37821	43721	33173	33241
Largest contig	158036	81042	**161446**	159367	64717	106190	139294	139293	80140	139290	82845	148754	158036
Total length	**102988100**	94836365	102540189	102842496	93026396	102180262	101021330	102718424	101620228	100768560	98311793	102877857	102814415
Reference length	100286070	100286070	100286070	100286070	100286070	100286070	100286070	100286070	100286070	100286070	100286070	100286070	100286070
GC (%)	38.56	38.56	38.52	38.55	38.52	38.54	38.49	38.52	38.38	38.57	38.75	38.56	38.57
Reference GC (%)	35.44	35.44	35.44	35.44	35.44	35.44	35.44	35.44	35.44	35.44	35.44	35.44	35.44
N50	5329	3414	**5560**	5365	3178	5208	4532	5400	4594	4285	3390	5329	5302
NG50	5534	3187	**5756**	5547	2898	5345	4581	5575	4657	4306	3304	5534	5509
N75	2461	1766	**2531**	2474	1665	2399	2169	2491	2201	2074	1685	2469	2461
NG75	2639	1534	**2690**	2650	1376	2527	2210	2662	2278	2099	1603	2640	2629
L50	4810	7512	**4580**	4817	7916	4997	5828	4844	5818	6134	7711	4825	4822
LG50	4561	8338	**4381**	4583	9111	4817	5747	4623	5674	6078	8006	4587	4588
L75	11969	17217	**11458**	11921	18097	12253	13928	11886	13827	14651	18076	11956	11958
LG75	11174	19698	**10810**	11171	21694	11675	13676	11177	13379	14477	18977	11194	11212
# misassemblies	**598**	1860	721	657	1678	757	990	675	830	1435	2774	665	628
# misassembled contigs	**594**	1787	708	654	1635	740	975	669	818	1380	2642	658	620
Misassembled contigs length	**3122101**	6343367	4041907	3343362	5160442	3652768	4258832	3495943	3923723	5540661	8112551	3344928	3242140
# local misassemblies	169	175	176	169	193	159	179	166	174	185	**120**	167	165
# unaligned mis. contigs	1	1	**0**	**0**	**0**	**0**	**0**	**0**	**0**	**0**	**0**	1	1
# unaligned contigs	4673 + 21 part	4271 + 42 part	4609 + 31 part	4709 + 22 part	**3856 + 42 part**	4949 + 28 part	5128 + 40 part	4783 + 29 part	5175 + 33 part	5273 + 32 part	5215 + 36 part	4690 + 23 part	4699 + 21 part
Unaligned length	15457774	13185257	15271613	15438170	**12771696**	15253161	14748271	15321756	14650849	14909845	14753767	15464643	15423697
Genome fraction (%)	**87.069**	81.060	86.857	86.948	79.860	86.459	85.771	86.946	86.467	85.306	82.599	86.948	86.934
Duplication ratio	**1.002**	1.004	**1.002**	**1.002**	**1.002**	1.003	1.003	**1.002**	1.003	1.004	1.009	**1.002**	**1.002**
# N's per 100 kbp	**0.00**	**0.00**	0.82	**0.00**	8.93	**0.00**	**0.00**	**0.00**	**0.00**	**0.00**	**0.00**	**0.00**	**0.00**
# mismatches per 100 kbp	**15.40**	44.37	15.81	17.49	22.64	17.76	47.72	17.15	45.84	110.51	101.37	16.20	15.46
# indels per 100 kbp	**8.60**	12.72	9.59	8.80	11.48	9.26	16.91	8.68	9.11	11.10	25.60	8.63	8.65
Largest alignment	51074	23778	**51077**	51069	22624	40495	40508	51069	36434	36039	33712	40497	51069
Total aligned length	**87429792**	81366347	87191165	87306376	80143767	86810073	86135010	87297553	86834040	85673728	83041928	87307082	87290550
NA50	3971	2519	**4112**	3992	2352	3889	3414	4039	3495	3181	2413	3975	3977
NGA50	4132	2329	**4241**	4140	2123	4008	3451	4181	3567	3202	2348	4131	4124
NA75	1382	1060	**1422**	1388	1024	1354	1258	1403	1309	1188	937	1382	1381
NGA75	1561	833	**1582**	1557	750	1474	1299	1569	1382	1212	864	1555	1545
LA50	6644	10118	**6360**	6600	10701	6699	7679	6505	7608	8167	10642	6629	6637
LGA50	6310	11243	**6090**	6285	12326	6459	7572	6210	7419	8092	11056	6309	6325
LA75	17451	24444	**16788**	17359	25515	17681	19681	17121	19304	20936	26838	17429	17429
LGA75	16071	28787	**15663**	16054	31704	16676	19249	15890	18560	20635	28482	16103	16131

4.2.8 *D. melanogaster*

Table 21 contains the Quast report after assembling dataset *D. melanogaster* with IDBA .

Table 21: Assembly quality metrics for *D. melanogaster*

Assembly	Uncorrected	ACE	BayesHammer	BFC	BLESS2	Blue	Fiona	Karect	Lighter	Musket	RACER	SGA-EC	Trowel
# contigs (≥ 0 bp)	223581	248508	208606	219200	159809	232478	225631	204944	227250	213768	210008	227076	216608
# contigs (≥ 1000 bp)	10040	11133	9437	9629	9261	9643	8912	8550	9507	8784	10450	9914	9747
# contigs (≥ 5000 bp)	4821	5486	4549	4709	5244	4582	4630	4330	4750	4656	5536	4800	4796
# contigs (≥ 10000 bp)	2952	3223	2830	2936	3387	2851	3031	2851	2985	3149	3487	2913	2935
# contigs (≥ 25000 bp)	1174	1065	1180	1183	1222	1169	1253	1238	1193	1305	1179	1181	1177
# contigs (≥ 50000 bp)	386	292	435	412	338	413	429	482	403	431	292	395	405
Total length (≥ 0 bp)	136848302	136462329	135724785	136392768	127932504	136892327	136300390	135379354	136339660	135162866	134486672	**137098799**	136237946
Total length (≥ 1000 bp)	114247383	112818345	114082986	114339445	113270501	113759034	114780121	**115028366**	114022280	114743642	114636046	114134174	114341269
Total length (≥ 5000 bp)	101715090	98832982	102305243	102547056	103248114	101718710	104544426	**105023741**	102447259	104854744	102687782	101822912	102455498
Total length (≥ 10000 bp)	88402381	82657559	90155500	89922855	89823586	89456079	93072964	**94439411**	89850423	93985779	88004113	88377312	89177890
Total length (≥ 25000 bp)	60421211	48860642	64093407	62278840	55230759	63079258	64942162	**68852849**	61545757	64708831	51593221	61036017	61243879
Total length (≥ 50000 bp)	32991908	22560206	38365354	35361693	24899345	36643703	36021083	**42374171**	34077796	34198243	20727594	33514313	34119167
# contigs	13390	13999	12526	12905	11272	12987	11912	11595	12438	11844	13716	13298	13078
Largest contig	444298	390345	420926	357542	390331	444337	408728	**444753**	444255	418612	197844	444372	390304
Total length	116592521	114830494	116237827	116630464	114687356	116107506	116882147	**117162385**	116068379	116889766	116922349	116504780	116670858
Reference length	120381546	120381546	120381546	120381546	120381546	120381546	120381546	120381546	120381546	120381546	120381546	120381546	120381546
GC (%)	42.60	42.60	42.59	42.59	42.59	42.61	42.60	42.59	42.56	42.61	42.60	42.59	42.60
Reference GC (%)	42.42	42.42	42.42	42.42	42.42	42.42	42.42	42.42	42.42	42.42	42.42	42.42	42.42
N50	26455	20337	29434	27934	24097	28588	29541	**33231**	27319	29410	21563	26890	27164
NG50	25129	18885	27801	26470	22588	27150	28304	**31616**	25898	28045	20684	25447	25730
N75	10391	8873	11384	11002	11346	11024	12504	**13413**	11183	12753	10116	10445	10768
NG75	9201	7545	9923	9797	9844	9590	11241	**12008**	9794	11724	9183	9215	9506
L50	1092	1444	960	1033	1309	982	1015	**880**	1059	1074	1475	1074	1066
LG50	1165	1586	1032	1102	1431	1058	1075	**930**	1140	1135	1557	1148	1136
L75	2858	3592	2552	2703	3031	2626	2548	**2282**	2721	2592	3456	2816	2774
LG75	3149	4101	2844	2973	3434	2936	2769	**2472**	3030	2807	3726	3113	3049
# misassemblies	**626**	698	628	664	748	670	658	647	640	672	967	639	670
# misassembled contigs	557	642	**553**	590	686	593	581	568	574	609	868	563	600
Misassembled contigs length	21033568	**17912825**	22874463	22837694	18568193	24054399	22543860	25176362	21502728	21660038	21288114	21894291	22474232
# local misassemblies	3655	3730	3808	3708	**1998**	3652	3435	3277	3582	2808	2638	3836	3560
# unaligned mis. contigs	31	21	25	28	**5**	30	20	16	26	16	9	38	29
# unaligned contigs	4185 + 473 part	3304 + 583 part	3948 + 540 part	4139 + 549 part	**2271 + 264 part**	4081 + 571 part	3946 + 429 part	4065 + 412 part	3633 + 499 part	3884 + 353 part	4014 + 348 part	4191 + 527 part	4139 + 470 part
Unaligned length	7951307	7080437	8027856	8020296	**4491455**	7866518	7404636	7604236	7344462	6897357	6814426	8129395	7896669
Genome fraction (%)	90.330	89.613	90.020	90.317	**91.527**	89.995	91.010	91.072	90.402	91.406	91.440	90.111	90.431
Duplication ratio	**0.999**	**0.999**	**0.999**	**0.999**	1.000	**0.999**	**0.999**	**0.999**	**0.999**	1.000	1.000	**0.999**	**0.999**
# N's per 100 kbp	8.65	7.31	18.24	7.22	49.90	7.18	6.37	5.91	7.00	5.00	**3.87**	7.67	7.76
# mismatches per 100 kbp	516.84	520.94	**513.77**	519.00	545.03	517.06	529.10	524.86	521.12	551.98	550.98	516.38	519.40
# indels per 100 kbp	130.93	131.77	**130.35**	132.28	141.13	130.74	134.60	131.52	131.03	134.06	141.09	130.55	131.89
Largest alignment	258232	202757	**259408**	258275	181773	258234	258107	258280	227789	258274	186904	258194	205327
Total aligned length	108390962	107468245	107979664	108367980	**109936320**	107999203	109234193	109329923	108475905	109736421	109757147	108127804	108527668
NA50	23503	18425	25924	24533	21970	25239	26026	**28465**	24293	25797	19085	23965	23998
NGA50	22381	17101	24534	23383	20512	23825	24990	**27415**	23152	24610	18420	22825	22928
NA75	8961	7631	9768	9426	10121	9434	10704	**11407**	9649	11103	8537	8938	9195
NGA75	7823	6313	8263	8230	8624	8044	9575	**10244**	8239	10032	7664	7801	8075
LA50	1253	1617	1124	1202	1453	1154	1170	**1037**	1220	1225	1661	1241	1233
LGA50	1335	1774	1206	1280	1587	1242	1239	**1094**	1311	1294	1753	1324	1312
LA75	3254	4034	2938	3107	3367	3035	2911	**2639**	3101	2943	3928	3224	3181
LGA75	3595	4634	3283	3426	3824	3403	3170	**2862**	3463	3192	4249	3573	3504

4.3 SPAdes

4.3.1 *B. dentium*

Table 22 contains the Quast report after assembling dataset *B. dentium* with SPAdes .

Table 22: Assembly quality metrics for *B. dentium*

Assembly	Uncorrected	ACE	BayesHammer	BFC	BLESS2	Blue	Fiona	Karect	Lighter	Musket	RACER	SGA-EC	Trowel
# contigs (≥ 0 bp)	1688	278	1653	1684	887	1664	1637	1680	1666	1682	1658	1678	1680
# contigs (≥ 1000 bp)	56	15	54	62	37	68	49	53	56	54	57	58	61
# contigs (≥ 5000 bp)	12	12	12	12	13	12	12	12	12	12	12	12	12
# contigs (≥ 10000 bp)	11	11	11	11	11	11	11	11	11	11	11	11	11
# contigs (≥ 25000 bp)	11	11	11	11	11	11	11	11	11	11	11	11	11
# contigs (≥ 50000 bp)	9	9	9	9	9	9	9	9	9	9	9	9	9
Total length (≥ 0 bp)	3518219	2733900	3503386	**3522389**	3046753	3518493	3487142	3514203	3507178	3516367	3503223	3516842	3522183
Total length (≥ 1000 bp)	2675554	2614800	2673009	2681531	2652461	**2693555**	2659416	2669659	2673430	2671485	2672971	2679544	2684632
Total length (≥ 5000 bp)	2610109	2610110	2610110	2610111	**2615377**	2610110	2610111	2610110	2610112	2610110	2610110	2610109	2610109
Total length (≥ 10000 bp)	2604505	2604506	2604506	2604507	2604505	2604506	2604507	2604506	**2604508**	2604506	2604506	2604505	2604505
Total length (≥ 25000 bp)	2604505	2604506	2604506	2604507	2604505	2604506	2604507	2604506	**2604508**	2604506	2604506	2604505	2604505
Total length (≥ 50000 bp)	2521362	2521363	2521363	2521364	2521362	2521363	2521364	2521363	**2521365**	2521363	2521363	2521362	2521362
# contigs	821	66	830	839	204	832	825	839	821	841	832	826	829
Largest contig	610215	610215	610215	610215	610215	610215	610215	610215	610215	610215	610215	610215	610215
Total length	3139081	2643783	3142820	3152040	2755449	**3153640**	3128460	3145410	3136958	3147715	3142543	3143479	3149960
Reference length	2636367	2636367	2636367	2636367	2636367	2636367	2636367	2636367	2636367	2636367	2636367	2636367	2636367
GC (%)	58.90	58.47	58.90	58.89	58.45	58.88	58.90	58.90	58.89	58.89	58.89	58.90	58.86
Reference GC (%)	58.54	58.54	58.54	58.54	58.54	58.54	58.54	58.54	58.54	58.54	58.54	58.54	58.54
N50	287948	**397392**	287949	287949	**397392**	287948	287949	287949	287949	287949	287948	287948	287948
NG50	397392	397392	397392	397392	397392	397392	397392	397392	397392	397392	397392	397392	397392
N75	158502	**177423**	158502	158502	**177423**	158502	158502	158502	158502	158502	158502	158502	158502
NG75	177423	177423	177423	177423	177423	177423	177423	177423	177423	177423	177423	177423	177423
L50	4	**3**	4	4	**3**	4	4	4	4	4	4	4	4
LG50	3	3	3	3	3	3	3	3	3	3	3	3	3
L75	8	**6**	8	8	**6**	8	8	8	8	8	8	8	8
LG75	6	6	6	6	6	6	6	6	6	6	6	6	6
# misassemblies	0	0	0	0	0	0	0	0	0	0	0	0	0
# misassembled contigs	0	0	0	0	0	0	0	0	0	0	0	0	0
Misassembled contigs length	0	0	0	0	0	0	0	0	0	0	0	0	0
# local misassemblies	3	3	3	3	3	3	3	3	3	3	3	3	3
# unaligned mis. contigs	**0**	7	1	**0**	**0**	**0**	**0**	**0**	**0**	**0**	**0**	**0**	**0**
# unaligned contigs	805 + 2 part	**34 + 2 part**	815 + 1 part	824 + 1 part	190 + 0 part	816 + 2 part	811 + 0 part	823 + 1 part	805 + 2 part	824 + 2 part	815 + 3 part	811 + 1 part	814 + 1 part
Unaligned length	525292	**21151**	529055	538427	141937	539856	514946	531251	523118	533413	528712	529836	536304
Genome fraction (%)	99.094	99.094	99.094	99.094	99.094	99.094	99.094	99.094	99.094	99.094	99.094	99.094	99.094
Duplication ratio	1.001	1.004	**1.000**	**1.000**	**1.000**	1.001	**1.000**	1.001	1.001	1.001	1.001	**1.000**	**1.000**
# N's per 100 kbp	0.00	0.00	0.00	0.00	0.00	0.00	0.00	0.00	0.00	0.00	0.00	0.00	0.00
# mismatches per 100 kbp	**0.23**	**0.23**	0.27	**0.23**	**0.23**	0.31	0.27	**0.23**	0.27	0.27	0.27	**0.23**	0.27
# indels per 100 kbp	0.27	0.31	0.31	0.27	0.27	**0.23**	0.27	0.31	0.31	0.31	**0.23**	0.27	0.27
Largest alignment	610215	610215	610215	610215	610215	610215	610215	610215	610215	610215	610215	610215	610215
Total aligned length	2613514	**2615318**	2613490	2613338	2613237	2613509	2613239	2613445	2613565	2613554	2613556	2613368	2613381
NA50	287948	**397392**	287949	287949	**397392**	287948	287949	287949	287949	287949	287948	287948	287948
NGA50	397392	397392	397392	397392	397392	397392	397392	397392	397392	397392	397392	397392	397392
NA75	158502	**177423**	158502	158502	**177423**	158502	158502	158502	158502	158502	158502	158502	158502
NGA75	177423	177423	177423	177423	177423	177423	177423	177423	177423	177423	177423	177423	177423
LA50	4	**3**	4	4	**3**	4	4	4	4	4	4	4	4
LGA50	3	3	3	3	3	3	3	3	3	3	3	3	3
LA75	8	**6**	8	8	**6**	8	8	8	8	8	8	8	8
LGA75	6	6	6	6	6	6	6	6	6	6	6	6	6

4.3.2 *E. coli str. K-12 substr. DH10B*

Table 23 contains the Quast report after assembling dataset *E. coli str. K-12 substr. DH10B* with SPAdes .

Table 23: Assembly quality metrics for *E. coli str. K-12 substr. DH10B*

Assembly	Uncorrected	ACE	BayesHammer	BFC	BLESS2	Blue	Fiona	Karect	Lighter	Musket	RACER	SGA-EC	Trowel
# contigs (≥ 0 bp)	186	117	109	125	114	122	116	114	112	121	121	134	134
# contigs (≥ 1000 bp)	79	79	78	79	78	79	79	81	78	79	79	79	78
# contigs (≥ 5000 bp)	61	62	61	62	62	61	61	61	61	62	62	61	61
# contigs (≥ 10000 bp)	54	55	54	55	55	54	54	54	54	55	55	54	54
# contigs (≥ 25000 bp)	49	49	49	49	49	49	49	49	49	49	49	49	49
# contigs (≥ 50000 bp)	29	28	29	28	28	29	29	29	29	28	28	29	29
Total length (≥ 0 bp)	**4390611**	4373352	4372108	4375195	4372035	4372602	4372343	4372889	4372776	4373454	4373398	4376447	4377075
Total length (≥ 1000 bp)	4365674	**4366114**	4365888	4366067	4364378	4363735	4365099	4365206	4365467	4365245	4365002	4365146	4365776
Total length (≥ 5000 bp)	4325946	4328231	4327992	**4328283**	4327750	4323821	4325928	4319347	4327639	4327413	4327169	4325543	4327967
Total length (≥ 10000 bp)	4269540	4271604	4271516	**4271867**	4271647	4267470	4269394	4262831	4271107	4270997	4270753	4269127	4271551
Total length (≥ 25000 bp)	4192073	4178567	**4194081**	4178971	4178836	4190037	4191951	4185397	4193674	4178118	4177918	4191660	4194019
Total length (≥ 50000 bp)	3456316	3395882	3455789	3396435	3396228	3455430	3453075	3453758	**3459882**	3395638	3395515	3453555	3455950
# contigs	83	82	81	82	81	82	81	84	81	82	82	82	81
Largest contig	326373	326271	326372	326373	**326386**	326277	326373	326372	326372	326372	326369	326373	326373
Total length	**4368476**	4368165	4367939	4368118	4366425	4365851	4366547	4367646	4367518	4367296	4367053	4367197	4367827
Reference length	4686137	4686137	4686137	4686137	4686137	4686137	4686137	4686137	4686137	4686137	4686137	4686137	4686137
GC (%)	50.75	50.75	50.75	50.75	50.75	50.75	50.75	50.75	50.75	50.75	50.75	50.75	50.75
Reference GC (%)	50.78	50.78	50.78	50.78	50.78	50.78	50.78	50.78	50.78	50.78	50.78	50.78	50.78
N50	108254	108227	**108384**	108254	108254	107774	107813	108228	107839	107839	107839	108254	108254
NG50	**97353**	**97353**	**97353**	**97353**	**97353**	97288	**97353**	**97353**	**97353**	**97353**	**97353**	**97353**	**97353**
N75	**57964**	57170	**57964**	57170	57147	**57964**	**57964**	**57964**	**57964**	57170	57147	**57964**	**57964**
NG75	**46124**	45930	**46124**	45889	45849	45891	**46124**	45941	**46124**	45889	45889	**46124**	**46124**
L50	13	13	13	13	13	13	13	13	13	13	13	13	13
LG50	14	14	14	14	14	14	14	14	14	14	14	14	14
L75	26	26	26	26	26	26	26	26	26	26	26	26	26
LG75	31	31	31	31	31	31	31	31	31	31	31	31	31
# misassemblies	**0**	**0**	**0**	**0**	**0**	1	**0**	**0**	1	**0**	**0**	**0**	**0**
# misassembled contigs	**0**	**0**	**0**	**0**	**0**	1	**0**	**0**	1	**0**	**0**	**0**	**0**
Misassembled contigs length	**0**	**0**	**0**	**0**	**0**	188639	**0**	**0**	91840	**0**	**0**	**0**	**0**
# local misassemblies	7	8	10	8	8	**6**	7	8	7	7	7	10	10
# structural variations	0	0	0	0	0	0	0	0	0	0	0	0	0
# unaligned mis. contigs	0	0	0	0	0	0	0	0	0	0	0	0	0
# unaligned contigs	0 + 0 part	0 + 0 part	0 + 0 part	0 + 0 part	0 + 0 part	0 + 0 part	0 + 0 part	0 + 0 part	0 + 0 part	0 + 0 part	0 + 0 part	0 + 0 part	0 + 0 part
Unaligned length	0	0	0	0	0	0	0	0	0	0	0	0	0
Genome fraction (%)	**93.179**	93.161	93.172	93.174	93.139	93.136	93.149	93.163	93.168	93.161	93.157	93.157	93.167
Duplication ratio	**1.000**	1.001	**1.000**	**1.000**	**1.000**	**1.000**	**1.000**	**1.000**	**1.000**	**1.000**	**1.000**	**1.000**	**1.000**
# N's per 100 kbp	5.08	4.85	**0.23**	4.85	4.86	5.08	5.08	5.08	5.08	4.85	4.85	**0.23**	**0.23**
# mismatches per 100 kbp	**3.57**	4.44	5.02	4.21	4.63	6.74	5.93	4.86	7.86	5.75	5.82	5.22	4.42
# indels per 100 kbp	0.46	0.50	0.48	0.48	0.48	0.62	0.57	**0.39**	0.57	0.50	0.64	0.44	0.48
Largest alignment	326373	326195	326372	326373	**326386**	326277	326373	326372	326372	326372	326369	326373	326373
Total aligned length	**4368147**	4367074	4367658	4367700	4365891	4365522	4366098	4367324	4367094	4366665	4366460	4366978	4367451
NA50	108254	108227	**108381**	108254	108254	107774	107813	108228	107839	107839	107839	108254	108254
NGA50	**97353**	**97353**	**97353**	**97353**	**97353**	97288	**97353**	**97353**	**97353**	**97353**	**97353**	**97353**	**97353**
NA75	**57964**	57170	**57964**	57170	57147	57170	**57964**	**57964**	**57964**	57170	57147	**57964**	**57964**
NGA75	**46124**	45854	**46124**	45889	45849	45889	**46124**	45940	**46124**	45889	45889	**46124**	**46124**
LA50	13	13	13	13	13	13	13	13	13	13	13	13	13
LGA50	14	14	14	14	14	14	14	14	14	14	14	14	14
LA75	**26**	**26**	**26**	**26**	**26**	27	**26**	**26**	**26**	**26**	**26**	**26**	**26**
LGA75	31	31	31	31	31	31	31	31	31	31	31	31	31

4.3.3 *E. coli str. K-12 substr. MG1655*

Table 24 contains the Quast report after assembling dataset *E. coli str. K-12 substr. MG1655* with SPAdes .

Table 24: Assembly quality metrics for *E. coli str. K-12 substr. MG1655*

Assembly	Uncorrected	ACE	BayesHammer	BFC	BLESS2	Blue	Fiona	Karect	Lighter	Musket	RACER	SGA-EC	Trowel
# contigs ($\geq$ 0 bp)	164	158	150	159	153	159	161	155	167	160	190	155	164
# contigs ($\geq$ 1000 bp)	81	78	79	81	83	78	82	80	79	81	82	82	82
# contigs ($\geq$ 5000 bp)	57	55	55	56	59	55	58	56	56	56	59	57	58
# contigs ($\geq$ 10000 bp)	53	51	51	52	55	51	54	52	52	51	55	53	54
# contigs ($\geq$ 25000 bp)	46	44	44	45	47	44	46	45	45	45	46	46	46
# contigs ($\geq$ 50000 bp)	32	30	30	31	33	30	32	31	31	31	30	32	32
Total length ($\geq$ 0 bp)	4574902	4572069	4571284	4573705	4571333	4571540	4573075	4573804	4574012	4573003	**4576108**	4572122	4573804
Total length ($\geq$ 1000 bp)	4552276	4550704	4550707	4552733	**4553419**	4549150	4551715	4552703	4550476	4551502	4550150	4552196	4551826
Total length ($\geq$ 5000 bp)	4498097	**4498467**	4496562	4497074	4497995	4497320	4498289	4497588	4498037	4494003	4498297	4496914	4497625
Total length ($\geq$ 10000 bp)	4468403	**4468853**	4466868	4467460	4468381	4467706	4468675	4467894	4468343	4457829	4468683	4467220	4467931
Total length ($\geq$ 25000 bp)	4365479	**4365929**	4363941	4364536	4350852	4364932	4353033	4364945	4365419	4365575	4326060	4364293	4352292
Total length ($\geq$ 50000 bp)	3878734	**3880534**	3877098	3878218	3878960	3879694	3867798	3878598	3880218	3880177	3756292	3877450	3865520
# contigs	93	88	92	93	91	91	95	92	91	93	94	93	94
Largest contig	264571	268093	285227	285527	264572	**285529**	264571	285527	264571	285527	264571	264571	264571
Total length	4560939	4557864	4560016	4561050	4559088	4558416	4560905	**4561354**	4559497	4559819	4558813	4560187	4560489
Reference length	4639675	4639675	4639675	4639675	4639675	4639675	4639675	4639675	4639675	4639675	4639675	4639675	4639675
GC (%)	50.74	50.75	50.74	50.74	50.75	50.75	50.74	50.75	50.74	50.74	50.74	50.74	50.74
Reference GC (%)	50.79	50.79	50.79	50.79	50.79	50.79	50.79	50.79	50.79	50.79	50.79	50.79	50.79
N50	133309	**133713**	133309	133309	126410	133309	133309	133309	133309	133309	133309	133189	133309
NG50	132876	**133713**	133309	133088	126410	133309	132876	133058	133309	133088	132876	132876	132876
N75	64785	67335	**67340**	64785	64785	67335	64692	64785	67335	64785	62691	64399	64585
NG75	60768	**64785**	64399	60768	60768	**64785**	62691	60768	**64785**	62691	59669	60768	60768
L50	13	**12**	**12**	**12**	14	**12**	13	**12**	13	**12**	13	13	13
LG50	14	**12**	**12**	13	14	**12**	14	13	13	13	14	14	14
L75	25	**23**	**23**	24	25	**23**	25	24	24	24	25	25	25
LG75	26	**24**	**24**	25	26	**24**	26	25	25	25	26	26	26
# misassemblies	0	0	0	0	0	0	0	0	0	0	0	0	0
# misassembled contigs	0	0	0	0	0	0	0	0	0	0	0	0	0
Misassembled contigs length	0	0	0	0	0	0	0	0	0	0	0	0	0
# local misassemblies	**5**	**5**	13	6	**5**	6	6	**5**	8	6	7	11	8
# unaligned mis. contigs	0	0	0	0	0	0	0	0	0	0	0	0	0
# unaligned contigs	4 + 0 part	2 + 0 part	4 + 0 part	4 + 0 part	**0 + 0 part**	4 + 0 part	5 + 0 part	4 + 0 part	4 + 0 part	4 + 0 part	4 + 0 part	4 + 0 part	4 + 0 part
Unaligned length	2946	1443	2947	2946	**0**	2946	3819	2946	2946	2946	2946	2946	2946
Genome fraction (%)	98.203	98.170	98.188	98.207	**98.219**	98.159	98.144	98.210	98.171	98.180	98.153	98.187	98.154
Duplication ratio	**1.000**	**1.000**	**1.000**	**1.000**	**1.000**	**1.000**	1.001	**1.000**	**1.000**	**1.000**	**1.000**	**1.000**	1.001
# N's per 100 kbp	0.00	0.00	0.00	0.00	0.00	0.00	0.00	0.00	0.00	0.00	0.00	0.00	0.00
# mismatches per 100 kbp	**2.28**	3.10	3.60	3.34	3.25	3.71	3.91	3.07	3.23	3.84	3.40	2.68	2.68
# indels per 100 kbp	0.50	0.44	0.55	0.46	**0.42**	0.53	0.51	0.50	0.50	0.50	0.44	0.55	0.53
Largest alignment	264571	268093	285227	285527	264572	**285529**	264571	285527	264571	285527	264571	264571	264571
Total aligned length	4557359	4555713	4556641	4557578	**4558342**	4555134	4554498	4557803	4555851	4556239	4555066	4556637	4555113
NA50	133309	**133713**	133309	133309	126372	133309	133309	133309	133309	133309	133309	133189	133309
NGA50	132876	**133713**	133309	133088	126372	133309	132876	133058	133309	133088	132876	132876	132876
NA75	64785	67335	**67340**	64785	64785	67335	64692	64785	67335	64785	62691	64399	64585
NGA75	60768	**64785**	64399	60768	60768	**64785**	62691	60768	**64785**	62691	59669	60768	60768
LA50	13	**12**	**12**	**12**	14	**12**	13	**12**	13	**12**	13	13	13
LGA50	14	**12**	**12**	13	14	**12**	14	13	13	13	14	14	14
LA75	25	**23**	**23**	24	25	**23**	25	24	24	24	25	25	25
LGA75	26	**24**	**24**	25	26	**24**	26	25	25	25	26	26	26

4.3.4 *S. enterica*

Table 25 contains the Quast report after assembling dataset *S. enterica* with SPAdes .

Table 25: Assembly quality metrics for *S. enterica*

Assembly	Uncorrected	ACE	BayesHammer	BFC	BLESS2	Blue	Fiona	Karect	Lighter	Musket	RACER	SGA-EC	Trowel
# contigs (≥ 0 bp)	164	68	74	115	85	104	88	89	91	96	120	136	124
# contigs (≥ 1000 bp)	26	25	26	26	28	28	24	24	25	25	27	26	26
# contigs (≥ 5000 bp)	20	17	19	19	21	19	19	18	19	19	21	19	19
# contigs (≥ 10000 bp)	19	16	18	18	20	18	18	17	18	18	20	18	18
# contigs (≥ 25000 bp)	18	15	17	17	19	17	17	16	17	17	19	17	17
# contigs (≥ 50000 bp)	15	13	14	14	15	14	14	14	14	14	15	14	14
Total length (≥ 0 bp)	**4832029**	4793717	4796241	4814811	4799092	4806157	4800907	4802582	4804245	4805547	4811040	4823822	4818253
Total length (≥ 1000 bp)	4777575	**4781314**	4779647	4780180	4779682	4780281	4777060	4778776	4778844	4778551	4777452	4780362	4779898
Total length (≥ 5000 bp)	4760565	**4762438**	4761734	4761892	4761770	4760166	4761628	4762066	4761834	4761767	4760713	4762074	4761833
Total length (≥ 10000 bp)	4754365	**4756238**	4755534	4755692	4755570	4753966	4755428	4755866	4755634	4755567	4754513	4755874	4755633
Total length (≥ 25000 bp)	4744007	**4745880**	4745176	4745334	4745212	4743653	4745070	4745508	4745276	4745209	4744155	4745516	4745275
Total length (≥ 50000 bp)	4639979	**4687841**	4641148	4641306	4610505	4639921	4641042	4687469	4641248	4641181	4609483	4641488	4641247
# contigs	39	30	33	38	38	36	36	34	37	35	40	36	37
Largest contig	**1022731**	**1022731**	**1022731**	**1022731**	**1022731**	1022686	**1022731**	**1022731**	**1022731**	**1022731**	**1022731**	**1022731**	**1022731**
Total length	4787310	4785068	4785034	**4788472**	4786552	4785774	4786046	4786049	4787682	4785715	4787246	4787493	4787748
Reference length	4888768	4888768	4888768	4888768	4888768	4888768	4888768	4888768	4888768	4888768	4888768	4888768	4888768
GC (%)	52.14	52.14	52.14	52.14	52.14	52.14	52.14	52.14	52.14	52.14	52.14	52.14	52.14
Reference GC (%)	52.09	52.09	52.09	52.09	52.09	52.09	52.09	52.09	52.09	52.09	52.09	52.09	52.09
N50	412097	**725282**	723537	723537	381864	723550	723537	**725282**	723537	723537	381603	723537	723537
NG50	412097	**725282**	723537	723537	381864	723550	723537	**725282**	723537	723537	381603	723537	723537
N75	235664	**298653**	235664	235664	231409	235230	235664	235664	235664	235664	231409	235664	235664
NG75	231409	**298653**	235664	235664	231409	235230	235664	235664	235664	235664	231409	235664	235664
L50	4	**3**	**3**	**3**	4	**3**	**3**	**3**	**3**	**3**	4	**3**	**3**
LG50	4	**3**	**3**	**3**	4	**3**	**3**	**3**	**3**	**3**	4	**3**	**3**
L75	7	**6**	7	7	8	7	7	7	7	7	8	7	7
LG75	8	**6**	7	7	8	7	7	7	7	7	8	7	7
# misassemblies	**12**	14	14	14	13	14	14	14	14	14	**12**	14	14
# misassembled contigs	8	**7**	**7**	**7**	8	**7**	**7**	**7**	**7**	**7**	8	**7**	**7**
Misassembled contigs length	3676571	3960170	3677740	3677898	3647205	3677455	3677707	3724134	3677839	3677773	**3646075**	3678080	3677839
# local misassemblies	**5**	**5**	6	**5**	6	6	**5**	**5**	**5**	**5**	**5**	**5**	**5**
# unaligned mis. contigs	0	0	0	0	0	0	0	0	0	0	0	0	0
# unaligned contigs	8 + 4 part	5 + 5 part	**5 + 4 part**	10 + 4 part	8 + 4 part	8 + 4 part	8 + 4 part	8 + 4 part	9 + 4 part	7 + 4 part	9 + 4 part	8 + 4 part	10 + 4 part
Unaligned length	65378	63645	**63216**	66496	64619	65150	64560	64446	66064	63864	65748	65335	66733
Genome fraction (%)	96.545	96.552	96.557	96.557	96.551	96.535	96.551	96.557	96.553	96.558	96.545	**96.560**	96.540
Duplication ratio	1.000	1.000	1.000	1.000	1.000	1.000	1.000	1.000	1.000	1.000	1.000	1.000	1.000
# N's per 100 kbp	0.00	0.00	0.00	0.00	0.00	0.00	0.00	0.00	0.00	0.00	0.00	0.00	0.00
# mismatches per 100 kbp	7.10	7.61	7.10	**6.91**	7.08	8.14	7.80	7.73	7.46	7.01	7.08	7.33	6.99
# indels per 100 kbp	0.64	0.64	0.61	**0.38**	0.61	0.68	0.74	0.64	0.66	0.64	0.61	0.42	0.61
Largest alignment	453219	453219	453219	453219	453219	453219	453219	453219	453219	453219	453219	453219	453219
Total aligned length	4721647	4721417	4721818	4721929	4721928	4720624	4721473	4721590	4721605	4721851	4721249	**4722158**	4721015
NA50	231409	**269985**	231409	231409	231409	231409	231409	231409	231409	231409	231409	231409	231409
NGA50	231409	231409	231409	231409	231409	231409	231409	231409	231409	231409	231409	231409	231409
NA75	140342	**161737**	140342	140342	140342	140342	140342	161730	140342	140342	140342	140342	140342
NGA75	133741	**161737**	133741	133741	133741	133930	133741	161730	133741	133741	133741	133741	133741
LA50	8	**7**	8	8	8	8	8	8	8	8	8	8	8
LGA50	8	8	8	8	8	8	8	8	8	8	8	8	8
LA75	14	14	14	14	14	14	14	14	14	14	14	14	14
LGA75	15	**14**	15	15	15	15	15	**14**	15	15	15	15	15

4.3.5 *P. aeruginosa*

Table 26 contains the Quast report after assembling dataset *P. aeruginosa* with SPAdes .

Table 26: Assembly quality metrics for *P. aeruginosa*

Assembly	Uncorrected	ACE	BayesHammer	BFC	BLESS2	Blue	Fiona	Karect	Lighter	Musket	RACER	SGA-EC	Trowel
# contigs (≥ 0 bp)	106	124	110	122	115	137	117	119	113	122	134	107	113
# contigs (≥ 1000 bp)	51	54	51	53	55	52	52	52	53	53	54	51	51
# contigs (≥ 5000 bp)	40	45	42	42	44	44	43	43	44	44	45	41	41
# contigs (≥ 10000 bp)	34	38	35	36	38	37	36	36	37	37	38	35	35
# contigs (≥ 25000 bp)	33	37	34	35	37	35	35	35	36	36	35	34	34
# contigs (≥ 50000 bp)	26	29	27	27	29	27	27	27	28	28	27	27	27
Total length (≥ 0 bp)	6230021	6230354	6230652	6230542	6228474	**6232837**	6230803	6230974	6230430	6231373	6231836	6229898	6230416
Total length (≥ 1000 bp)	6216016	6215931	**6217165**	6214679	6214823	6214235	6215260	6215595	6215793	6215930	6215641	6215415	6215526
Total length (≥ 5000 bp)	6197160	6202121	**6203355**	6195823	6195967	6201464	6201450	6201785	6201983	6202120	6201831	6197595	6197706
Total length (≥ 10000 bp)	6160776	6160178	**6161412**	6159439	6159583	6159614	6159507	6159896	6160040	6160177	6159888	6161211	6161322
Total length (≥ 25000 bp)	6136845	6136247	**6137481**	6135508	6135652	6122306	6135576	6135965	6136109	6136246	6096890	6137280	6137391
Total length (≥ 50000 bp)	**5871704**	5831636	5862721	5831381	5831525	5818247	5831278	5831642	5831786	5831635	5796958	5862520	5862631
# contigs	59	60	58	61	62	61	59	60	61	60	62	59	59
Largest contig	582545	582545	582545	582545	582545	582232	582232	582747	582545	582545	**742310**	582545	582545
Total length	6221236	6220118	**6221612**	6219899	6219517	6220757	6220286	6220815	6221013	6220643	6220861	6220851	6220962
Reference length	6264404	6264404	6264404	6264404	6264404	6264404	6264404	6264404	6264404	6264404	6264404	6264404	6264404
GC (%)	66.58	66.58	66.58	66.58	66.58	66.58	66.58	66.58	66.58	66.58	66.58	66.58	66.58
Reference GC (%)	66.56	66.56	66.56	66.56	66.56	66.56	66.56	66.56	66.56	66.56	66.56	66.56	66.56
N50	**289353**	264881	**289353**	**289353**	264881	289314	**289353**	**289353**	264881	264881	264881	**289353**	**289353**
NG50	**289353**	264881	**289353**	**289353**	264881	289314	**289353**	**289353**	264881	264881	264881	**289353**	**289353**
N75	**210881**	165623	165623	165623	119649	165623	165623	165623	165623	165623	165623	165623	165623
NG75	**210881**	165623	165623	165623	119649	165623	165623	165623	165623	165623	165623	165623	165623
L50	**7**	8	**7**	**7**	8	**7**	**7**	**7**	8	8	**7**	**7**	**7**
LG50	**7**	8	**7**	**7**	8	**7**	**7**	**7**	8	8	**7**	**7**	**7**
L75	**14**	15	**14**	**14**	15	**14**	**14**	**14**	15	15	**14**	**14**	**14**
LG75	**14**	15	**14**	**14**	15	**14**	**14**	**14**	15	15	**14**	**14**	**14**
# misassemblies	**3**	**3**	**3**	**3**	**3**	**3**	**3**	**3**	**3**	**3**	4	**3**	**3**
# misassembled contigs	3	3	3	3	3	3	3	3	3	3	3	3	3
Misassembled contigs length	565341	565352	565341	565341	565459	**565086**	565352	565341	565341	565352	920163	565341	565341
# local misassemblies	5	7	6	**4**	7	6	**4**	5	**4**	**4**	6	7	9
# unaligned mis. contigs	0	0	0	0	0	0	0	0	0	0	0	0	0
# unaligned contigs	1 + 2 part	**0 + 2 part**	1 + 3 part	1 + 2 part	**0 + 2 part**	1 + 2 part	1 + 2 part	1 + 2 part	1 + 2 part	1 + 2 part	1 + 2 part	1 + 3 part	1 + 3 part
Unaligned length	11429	**10903**	12478	11429	**10903**	11429	11429	11429	11429	11429	11429	12478	12478
Genome fraction (%)	**99.088**	99.074	99.070	99.072	99.068	99.075	99.078	99.080	99.083	99.079	99.079	99.061	99.057
Duplication ratio	**1.000**	**1.000**	**1.000**	**1.000**	**1.000**	**1.000**	**1.000**	**1.000**	**1.000**	**1.000**	**1.000**	**1.000**	1.001
# N's per 100 kbp	18.39	18.55	17.95	17.80	18.28	19.74	17.80	18.71	18.55	18.55	17.79	**17.49**	17.96
# mismatches per 100 kbp	**2.84**	3.35	4.03	3.06	3.56	3.93	3.16	3.35	3.09	3.16	3.58	3.72	4.03
# indels per 100 kbp	**0.84**	0.85	0.93	0.87	0.87	1.02	0.85	**0.84**	0.87	0.85	0.90	0.92	0.98
Largest alignment	582545	582545	582545	582545	582545	582232	582232	**582618**	582545	582545	582545	582545	582545
Total aligned length	**6209300**	6208656	6208592	6208168	6208014	6209026	6208555	6208955	6209077	6208733	6208835	6207866	6208060
NA50	**289353**	264881	264881	264881	264881	264881	264881	264881	264881	264881	264881	264881	264881
NGA50	**289353**	264881	264881	264881	264881	264881	264881	264881	264881	264881	264881	264881	264881
NA75	**210881**	165623	165623	165623	119649	165623	165623	165623	165623	165623	165623	165623	165623
NGA75	**210881**	119649	165623	165623	119649	165623	165623	165623	165623	165623	165623	165623	165623
LA50	**7**	8	8	8	8	8	8	8	8	8	8	8	8
LGA50	**7**	8	8	8	8	8	8	8	8	8	8	8	8
LA75	**14**	15	**14**	**14**	15	**14**	**14**	**14**	15	15	15	**14**	**14**
LGA75	**14**	16	**14**	**14**	15	**14**	**14**	**14**	15	15	15	**14**	**14**

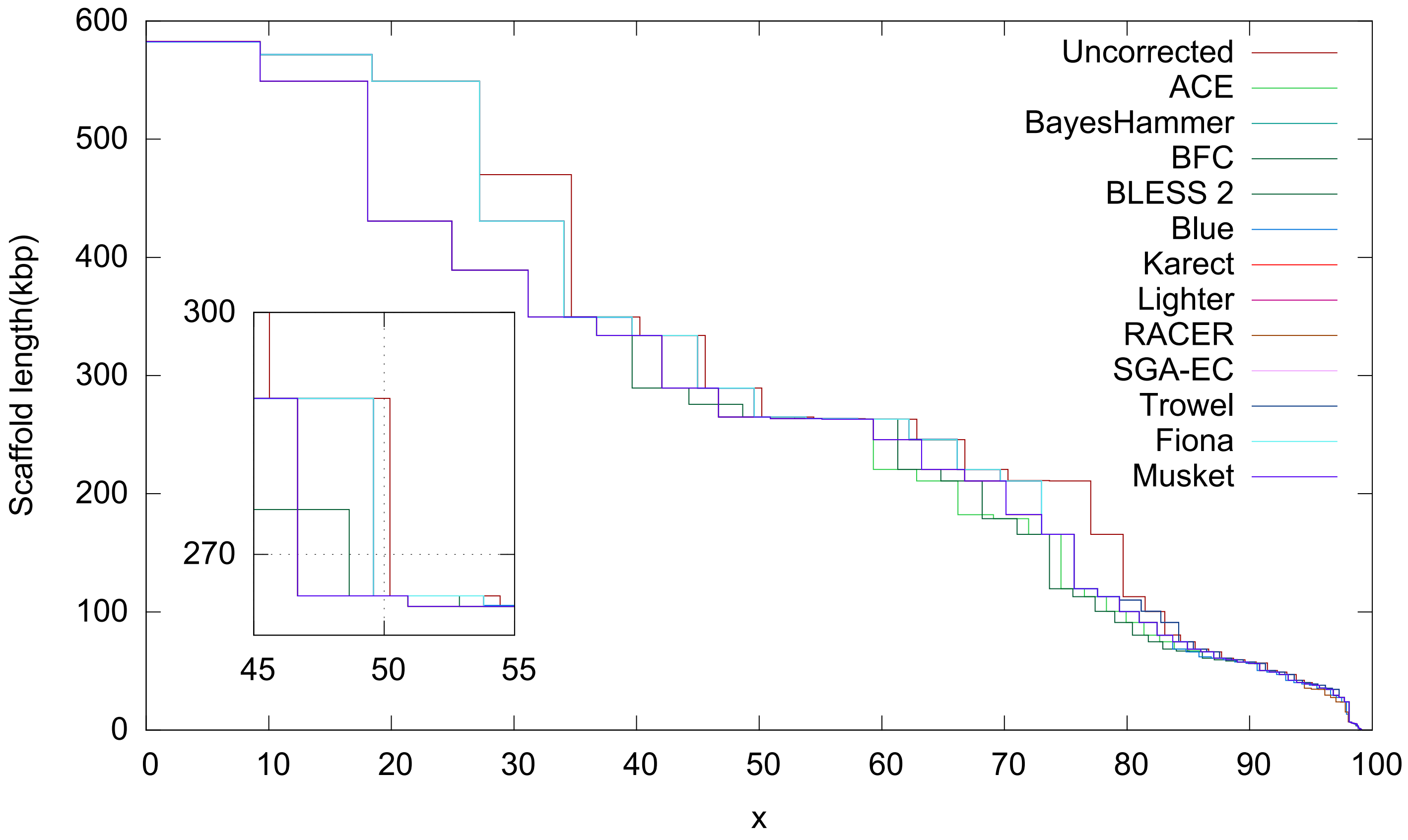

Figure 1: SPAdes assembly results for P. aeruginosa for both uncorrected and corrected data. Scaffolds with length NGAx or larger produce x% of the genome.

4.3.6 *H. sapiens* Chr. 21

Table 27 contains the Quast report after assembling dataset *H. sapiens* Chr. 21 with SPAdes .

Table 27: Assembly quality metrics for *H. sapiens* Chr. 21

Assembly	Uncorrected	ACE	BayesHammer	BFC	BLESS2	Blue	Fiona	Karect	Lighter	Musket	RACER	SGA-EC	Trowel
# contigs (≥ 0 bp)	11658	13066	10876	11247	13635	15666	11618	12646	12001	12350	13682	11803	13586
# contigs (≥ 1000 bp)	3661	3488	3376	3420	3925	3935	3368	3279	3425	3416	3879	3408	3969
# contigs (≥ 5000 bp)	1987	1961	1925	1911	2034	2085	1943	1897	1917	1929	2026	1919	2048
# contigs (≥ 10000 bp)	1145	1135	1119	1140	1098	1119	1142	1150	1137	1136	1090	1149	1103
# contigs (≥ 25000 bp)	246	250	279	282	216	210	271	295	279	274	215	270	214
# contigs (≥ 50000 bp)	24	29	27	27	12	20	32	32	23	36	17	30	17
Total length (≥ 0 bp)	34305614	34380522	34123100	34286645	34220998	**34449556**	34242236	34445755	34374980	34350021	34383404	34381972	34389916
Total length (≥ 1000 bp)	32945791	32944930	32860903	32985264	32700258	32769213	32955487	**33047117**	32997396	32974244	32838152	33005502	32846237
Total length (≥ 5000 bp)	28720164	29021061	29168621	29179374	27803086	27971993	29261315	**29545706**	29162965	29238235	27997267	29201336	27915791
Total length (≥ 10000 bp)	22672318	23065855	23340510	23624964	21088444	21094246	23475955	**24143915**	23553674	23512065	21260122	23687963	21057378
Total length (≥ 25000 bp)	8707938	9282473	10143177	10229508	7489603	7406913	10060749	**10705450**	10021930	10100507	7680611	9985753	7449787
Total length (≥ 50000 bp)	1423296	1891297	1724169	1679438	781510	1218193	2073928	2036281	1482391	**2208760**	1047851	1898371	1018260
# contigs	4303	4082	3948	4032	4584	4635	3949	3838	4038	3997	4551	4008	4702
Largest contig	74497	103923	82977	109258	82937	90065	**136866**	111589	90223	105061	85668	112665	85021
Total length	33409153	33370188	33274939	33424565	33177053	33276648	33371010	**33445850**	33437566	33390351	33323920	33437887	33379800
Reference length	46709983	46709983	46709983	46709983	46709983	46709983	46709983	46709983	46709983	46709983	46709983	46709983	46709983
GC (%)	40.79	40.82	40.76	40.80	40.75	40.79	40.81	40.82	40.81	40.81	40.79	40.81	40.78
Reference GC (%)	42.22	42.22	42.22	42.22	42.22	42.22	42.22	42.22	42.22	42.22	42.22	42.22	42.22
N50	15229	15787	16485	16484	13678	13214	15905	**17170**	16666	16419	13876	16495	13493
NG50	9391	9717	9988	10203	8262	8194	10128	**10819**	10189	10172	8448	10303	8291
N75	7932	8326	8555	8647	7180	7076	8718	**9161**	8521	8622	7219	8673	7193
L50	662	629	600	604	716	735	608	**588**	610	606	702	607	729
LG50	1216	1165	1121	1114	1347	1369	1130	**1074**	1118	1121	1318	1117	1355
L75	1412	1351	1293	1296	1546	1579	1309	**1249**	1302	1301	1529	1299	1573
# misassemblies	205	203	224	202	191	168	203	**119**	179	200	358	207	222
# misassembled contigs	196	188	207	186	183	159	179	**112**	161	184	331	196	208
Misassembled contigs length	3187244	3357505	3896326	3645633	2616542	2508072	3576383	**2217240**	3109131	3728907	5318815	3876193	3101965
# local misassemblies	148	128	153	134	**111**	121	143	134	133	146	134	141	134
# structural variations	1	0	1	1	0	1	1	0	1	1	1	1	1
# unaligned mis. contigs	2	**0**	1	1	**0**	1	**0**	1	**0**	**0**	**0**	2	**0**
# unaligned contigs	90 + 17 part	72 + 11 part	84 + 16 part	93 + 15 part	58 + 17 part	**50 + 13 part**	66 + 11 part	74 + 11 part	95 + 12 part	70 + 14 part	73 + 14 part	92 + 10 part	94 + 18 part
Unaligned length	107345	80003	115175	108173	76973	**67157**	79411	91569	108183	90024	98217	105816	111030
Genome fraction (%)	70.891	70.884	70.744	70.953	70.540	70.699	70.936	**71.080**	70.978	70.942	70.706	70.986	70.800
Duplication ratio	1.006	1.005	**1.003**	1.005	1.005	1.006	1.005	1.005	1.005	1.005	1.006	1.005	1.006
# N's per 100 kbp	12.56	13.76	11.38	12.28	28.84	14.08	**8.36**	15.66	14.59	13.19	10.92	12.46	9.72
# mismatches per 100 kbp	177.83	179.97	169.06	173.89	176.87	186.06	177.09	**168.19**	175.09	176.42	188.03	174.83	183.62
# indels per 100 kbp	37.28	37.81	37.16	**36.80**	37.01	37.70	38.14	36.96	37.49	37.43	38.00	37.48	37.17
Largest alignment	74497	103923	82445	90912	81005	90065	**136863**	111527	90220	103129	85647	90220	85007
Total aligned length	33233678	33218305	33132429	33254831	33047536	33140710	33236923	**33302749**	33270483	33245187	33141015	33274470	33199144
NA50	14406	15025	15545	15471	13158	12820	15154	**16481**	15781	15462	12579	15379	12815
NGA50	8829	9190	9443	9578	7802	7754	9491	**10302**	9618	9502	7603	9640	7863
NA75	7453	7800	8104	8018	6747	6733	8143	**8723**	8075	8070	6562	8040	6837
LA50	694	664	636	640	740	763	647	**611**	643	644	772	645	761
LGA50	1280	1231	1188	1184	1404	1428	1198	**1115**	1180	1191	1452	1188	1419
LA75	1490	1428	1371	1379	1614	1650	1388	**1297**	1375	1384	1684	1384	1648

4.3.7 *C. elegans*

Table 28 contains the Quast report after assembling dataset *C. elegans* with SPAdes .

Table 28: Assembly quality metrics for *C. elegans*

Assembly	Uncorrected	ACE	BayesHammer	BFC	BLESS2	Blue	Fiona	Karect	Lighter	Musket	RACER	SGA-EC	Trowel
# contigs (≥ 0 bp)	64272	81082	62466	64735	87989	67408	70638	65792	67043	70931	78836	64022	65391
# contigs (≥ 1000 bp)	19715	26128	19230	19773	27860	19976	21281	19728	20822	22405	24680	19671	19746
# contigs (≥ 5000 bp)	6322	5237	6324	6345	4809	6255	6277	6345	6432	6157	5674	6320	6322
# contigs (≥ 10000 bp)	2427	1174	2456	2405	972	2373	2147	2428	2246	1919	1545	2418	2404
# contigs (≥ 25000 bp)	291	123	316	298	73	302	241	308	251	201	152	294	291
# contigs (≥ 50000 bp)	69	34	73	65	8	64	54	64	58	53	39	68	66
Total length (≥ 0 bp)	116531376	112665206	115842055	116541087	112459609	116159435	115776939	116265023	115948004	115599759	114392779	116406566	**116593128**
Total length (≥ 1000 bp)	**104888717**	96488385	104286506	104761637	93758025	104056252	102742327	104449183	103511220	102076721	98489212	104803286	104798953
Total length (≥ 5000 bp)	72682147	47647948	**73360701**	72602210	40794116	71152597	67007401	72377260	69150530	63681095	54401186	72740724	72574356
Total length (≥ 10000 bp)	45311588	20068019	**46103656**	44926759	14879662	43803433	38056990	44781786	39819043	34047844	26049004	45279671	45056640
Total length (≥ 25000 bp)	13999398	5661850	**14795023**	14054191	2637052	13691065	10590733	13714349	11128377	9601227	6786678	14041231	14181993
Total length (≥ 50000 bp)	6809697	2551613	**6959909**	6455562	459382	6022022	4471501	5853071	4791869	4687814	3002620	6744537	6897053
# contigs	26846	37484	26495	27029	40819	27393	29558	26932	28780	31318	35887	26806	26974
Largest contig	240969	128379	240394	244022	66400	240394	164676	238991	232377	240394	152332	263254	**402380**
Total length	**109983290**	104619147	109484481	109949860	103011932	109370494	108661357	109600335	109201095	108455777	106522260	109900955	109964382
Reference length	100286070	100286070	100286070	100286070	100286070	100286070	100286070	100286070	100286070	100286070	100286070	100286070	100286070
GC (%)	38.47	38.28	38.40	38.45	38.19	38.44	38.33	38.41	38.34	38.38	38.57	38.46	38.46
Reference GC (%)	35.44	35.44	35.44	35.44	35.44	35.44	35.44	35.44	35.44	35.44	35.44	35.44	35.44
N50	7948	4512	**8157**	7871	3931	7708	6873	7923	7154	6334	5139	7959	7913
NG50	8930	4738	**9140**	8876	4045	8667	7551	8861	7925	6946	5518	8926	8888
N75	3654	2270	**3736**	3636	2011	3538	3208	3633	3366	2957	2436	3661	3629
NG75	4633	2514	**4710**	4615	2142	4421	3949	4573	4191	3597	2846	4631	4606
L50	3515	6219	**3411**	3538	7235	3611	4119	3554	4000	4475	5450	3505	3522
LG50	2939	5750	**2879**	2960	6894	3055	3538	2998	3408	3859	4864	2934	2945
L75	8614	14423	**8346**	8658	16402	8846	9889	8652	9540	10757	12969	8585	8648
LG75	6849	13062	**6706**	6890	15417	7121	8124	6940	7759	8881	11193	6835	6874
# misassemblies	1377	4278	1460	**1332**	1654	1603	2032	1335	2025	2103	8293	1392	1377
# misassembled contigs	1296	3858	1371	**1254**	1593	1510	1890	1257	1874	1963	6560	1310	1297
Misassembled contigs length	9735210	17244516	10432238	9391106	**5928792**	10781051	12262942	9392527	12972582	11134702	31623064	9855510	9569483
# local misassemblies	399	326	433	392	**309**	412	473	414	394	425	346	409	401
# unaligned mis. contigs	**7**	21	9	8	13	9	9	**7**	9	12	20	**7**	10
# unaligned contigs	4743 + 91 part	**4049 + 169 part**	4899 + 88 part	4789 + 86 part	4465 + 88 part	4756 + 97 part	4798 + 108 part	4807 + 79 part	4904 + 104 part	4886 + 98 part	4976 + 177 part	4770 + 92 part	4739 + 81 part
Unaligned length	16953817	14482847	16700997	16905208	**14030377**	16726451	16200042	16687086	16399595	16371253	16133966	16937864	16931221
Genome fraction (%)	92.273	87.960	92.152	**92.298**	88.334	91.876	91.576	92.187	91.949	91.161	88.855	92.212	92.276
Duplication ratio	1.005	1.022	**1.004**	1.005	**1.004**	1.005	1.007	1.005	1.006	1.007	1.014	1.005	1.005
# N's per 100 kbp	144.78	**112.56**	148.00	144.96	115.15	148.93	144.91	144.51	144.71	143.52	136.35	146.95	146.14
# mismatches per 100 kbp	33.27	117.20	**30.55**	33.15	37.24	39.77	87.49	33.57	80.67	177.01	155.25	33.51	33.23
# indels per 100 kbp	7.22	19.86	7.82	**7.17**	8.24	8.15	22.19	7.31	8.01	10.97	21.52	7.32	7.20
Largest alignment	50836	27416	**64368**	50836	27155	50836	50837	64367	50836	41089	40668	50836	50836
Total aligned length	92814637	88415367	92662358	**92838725**	88802236	92418941	92206639	92718109	92538398	91814436	89541690	92751467	92825410
NA50	5625	2979	**5795**	5605	2928	5432	4799	5623	5037	4404	2979	5640	5593
NGA50	6472	3158	**6576**	6437	3036	6183	5385	6431	5654	4952	3266	6483	6423
NA75	1878	1124	**1939**	1874	1210	1815	1658	1901	1721	1526	1037	1880	1872
NGA75	2805	1348	**2849**	2788	1338	2644	2341	2782	2488	2143	1347	2799	2787
LA50	5079	9487	**4925**	5093	9672	5224	5940	5055	5767	6448	9342	5061	5102
LGA50	4274	8780	**4181**	4288	9214	4438	5117	4280	4931	5572	8343	4265	4293
LA75	13282	23377	**12846**	13314	23124	13702	15293	13194	14769	16627	24133	13249	13343
LGA75	10140	20740	**9931**	10174	21516	10617	12131	10183	11563	13253	20188	10133	10193

4.3.8 *D. melanogaster*

Table 29 contains the Quast report after assembling dataset *D. melanogaster* with SPAdes .

Table 29: Assembly quality metrics for *D. melanogaster*

Assembly	Uncorrected	ACE	BayesHammer	BFC	BLESS2	Blue	Fiona	Karect	Lighter	Musket	RACER	SGA-EC	Trowel
# contigs ($\geq$ 0 bp)	74043	78599	69271	73234	76430	74610	80861	74173	71364	80075	86186	70023	72210
# contigs ($\geq$ 1000 bp)	5483	6789	5454	5543	6771	5503	5900	5431	5523	6199	9213	5480	5650
# contigs ($\geq$ 5000 bp)	2533	3751	2550	2593	3730	2558	2731	2456	2640	2934	4623	2530	2643
# contigs ($\geq$ 10000 bp)	1933	2754	1922	1951	2750	1915	2084	1844	1992	2212	3195	1907	2007
# contigs ($\geq$ 25000 bp)	1206	1442	1193	1211	1402	1197	1271	1177	1229	1304	1412	1182	1235
# contigs ($\geq$ 50000 bp)	704	626	701	691	615	679	711	700	711	700	498	687	692
Total length ($\geq$ 0 bp)	130030442	128478789	129365298	129911559	127133184	129521097	**130605696**	129982716	129327660	130215917	130591397	129618485	129655323
Total length ($\geq$ 1000 bp)	120972662	119245763	120687956	120958052	118182463	120423367	120885087	**121024055**	120546143	120456395	120306993	120966385	120866556
Total length ($\geq$ 5000 bp)	**114719681**	112180360	114535094	114657226	111207133	114260288	114138370	114718069	114368253	113524107	109812670	114690805	114379771
Total length ($\geq$ 10000 bp)	**110478651**	105048796	110093513	110109175	104211872	109698057	109584716	110408099	109785315	108438548	99585998	110279095	109845156
Total length ($\geq$ 25000 bp)	98656926	83306275	98285329	97985919	82067268	97987147	96415034	**99512924**	97286836	93500096	70429725	98360112	97108585
Total length ($\geq$ 50000 bp)	80423462	54558355	80415030	79115237	54255186	78950991	75950200	**81985969**	78612470	71818362	38126731	80250129	77361963
# contigs	8454	9707	8451	8575	9382	8580	8924	8361	8514	9443	12816	8461	8613
Largest contig	518444	344897	522527	518411	440228	482029	433735	518285	592584	367481	243181	598856	**686399**
Total length	123060751	121275348	122787001	123078343	119991674	122588231	123008213	**123082336**	122638571	122729429	122836325	123059826	122956447
Reference length	120381546	120381546	120381546	120381546	120381546	120381546	120381546	120381546	120381546	120381546	120381546	120381546	120381546
GC (%)	42.56	42.52	42.53	42.57	42.51	42.58	42.58	42.57	42.55	42.59	42.58	42.57	42.57
Reference GC (%)	42.42	42.42	42.42	42.42	42.42	42.42	42.42	42.42	42.42	42.42	42.42	42.42	42.42
N50	83804	43877	84315	82203	43315	83277	71630	**88533**	79363	63519	31144	84694	77879
NG50	86505	44167	85854	84499	43084	84749	73544	**91730**	81203	65332	31761	87595	80342
N75	34576	19862	34016	32845	19401	34311	30651	**36690**	32157	26676	13867	34383	31539
NG75	37360	20342	36806	35722	19244	36580	33263	**39101**	34418	28621	14802	37283	34705
L50	411	756	408	413	739	404	470	**393**	434	516	1091	397	436
LG50	395	746	394	396	743	391	452	**378**	420	497	1052	381	419
L75	990	1785	982	1015	1761	990	1122	**941**	1043	1248	2562	975	1061
LG75	934	1752	932	956	1776	944	1060	**888**	991	1185	2433	919	1003
# misassemblies	1100	1287	1133	1114	1304	1201	1152	**1098**	1152	1225	2236	1111	1163
# misassembled contigs	**846**	1063	861	856	1079	915	907	850	886	948	1751	847	889
Misassembled contigs length	53566639	40875670	55491362	53723282	**40614325**	57041407	51435560	54512203	55267465	50221260	49962445	55594508	55519157
# local misassemblies	1959	1875	**1417**	2007	2186	1985	1986	1846	1935	1953	1863	1903	1951
# unaligned mis. contigs	97	102	106	106	98	99	114	102	**87**	110	107	97	110
# unaligned contigs	3523 + 877 part	2780 + 902 part	3468 + 863 part	3507 + 899 part	**2298 + 788 part**	3556 + 838 part	3570 + 887 part	3507 + 866 part	3392 + 892 part	3689 + 885 part	3882 + 966 part	3518 + 889 part	3534 + 887 part
Unaligned length	8655611	7015647	8461083	8663161	**5974560**	8325736	8619025	8706997	8247034	8400074	8510536	8653846	8602555
Genome fraction (%)	94.389	94.126	**94.395**	94.385	94.007	94.283	94.339	94.381	94.364	94.288	94.094	94.390	94.340
Duplication ratio	1.007	1.008	**1.006**	1.007	1.008	1.007	1.007	1.007	1.007	1.007	1.009	1.007	1.007
# N's per 100 kbp	20.77	23.70	20.46	20.90	80.37	**19.31**	24.51	21.24	19.63	25.30	21.68	19.56	20.58
# mismatches per 100 kbp	550.95	566.10	550.06	549.94	562.32	552.77	553.75	**547.82**	551.94	574.04	572.70	549.10	548.28
# indels per 100 kbp	131.06	137.55	132.17	130.71	134.83	131.25	131.77	130.85	131.13	132.41	132.97	130.86	**130.28**
Largest alignment	446343	201781	357935	428474	285019	**446353**	406631	446139	375228	269466	179030	408292	408315
Total aligned length	113947282	113635974	113933062	113945277	113517804	113788556	113908464	113929648	113921610	113834640	113679126	**113951223**	113890263
NA50	59109	35127	57232	57455	35199	57276	53453	**60755**	54973	47234	23144	59109	54194
NGA50	60554	35392	58570	59441	34884	59109	54188	**62304**	56348	48694	23783	60636	55841
NA75	24325	15983	24327	23793	16032	23582	22424	**26073**	23625	20198	9811	24613	22828
NGA75	26503	16373	26218	25875	15841	25252	24142	**28090**	25252	21704	10521	26802	24540
LA50	570	945	582	575	930	576	640	**548**	606	700	1433	564	608
LGA50	548	932	561	552	936	557	616	**526**	586	676	1381	542	585
LA75	1374	2222	1393	1400	2202	1399	1517	**1307**	1449	1682	3435	1360	1474
LGA75	1295	2181	1321	1318	2221	1331	1432	**1232**	1379	1598	3254	1282	1392

4.4 Velvet

4.4.1 *B. dentium*

Table 30 contains the Quast report after assembling dataset *B. dentium* with Velvet .

Table 30: Assembly quality metrics for *B. dentium*

Assembly	Uncorrected	ACE	BayesHammer	BFC	BLESS2	Blue	Fiona	Karect	Lighter	Musket	RACER	SGA-EC	Trowel
# contigs (≥ 0 bp)	164979	73360	59552	70557	44383	36024	76459	30869	76687	68649	49049	135918	108793
# contigs (≥ 1000 bp)	5	1	556	464	792	922	193	926	242	314	916	38	67
# contigs (≥ 5000 bp)	0	0	0	0	5	27	0	27	0	0	21	0	0
# contigs (≥ 10000 bp)	0	0	0	0	0	0	0	1	0	0	1	0	0
# contigs (≥ 25000 bp)	0	0	0	0	0	0	0	0	0	0	0	0	0
# contigs (≥ 50000 bp)	0	0	0	0	0	0	0	0	0	0	0	0	0
Total length (≥ 0 bp)	**13904588**	5670761	8730630	9747006	7400138	7597563	9809249	7139160	10007466	9327465	8440471	12986853	11430326
Total length (≥ 1000 bp)	5777	1010	807224	666417	1372997	1876244	254328	**1972180**	314586	422998	1835347	43440	84111
Total length (≥ 5000 bp)	0	0	0	0	30442	167860	0	**179556**	0	0	129097	0	0
Total length (≥ 10000 bp)	0	0	0	0	0	0	0	10815	0	0	**12218**	0	0
Total length (≥ 25000 bp)	0	0	0	0	0	0	0	0	0	0	0	0	0
Total length (≥ 50000 bp)	0	0	0	0	0	0	0	0	0	0	0	0	0
# contigs	508	39	2161	2003	1767	1982	1581	1930	1713	1894	1944	925	1210
Largest contig	1429	1010	3784	4501	7416	9719	3745	10815	2583	3552	**12218**	1758	3169
Total length	303382	22970	1878464	1687429	2058670	2584183	1153196	**2634272**	1284413	1470681	2521612	591102	805395
Reference length	2636367	2636367	2636367	2636367	2636367	2636367	2636367	2636367	2636367	2636367	2636367	2636367	2636367
GC (%)	59.89	49.27	58.96	58.91	58.56	58.75	58.62	58.81	58.80	58.86	58.70	58.75	58.91
Reference GC (%)	58.54	58.54	58.54	58.54	58.54	58.54	58.54	58.54	58.54	58.54	58.54	58.54	58.54
N50	576	552	908	860	1330	1660	701	**1834**	733	768	1638	603	634
NG50	-	-	676	598	1054	1616	-	1832	-	541	1576	-	-
N75	540	519	630	617	846	930	580	**993**	585	594	945	551	557
NG75	-	-	-	-	553	891	-	992	-	-	851	-	-
L50	227	**18**	695	655	496	468	588	441	625	672	468	387	487
LG50	-	-	1184	1329	739	484	-	442	-	1601	504	-	-
L75	363	**28**	1323	1243	978	987	1045	930	1119	1221	974	644	829
LG75	-	-	-	-	1612	1030	-	932	-	-	1070	-	-
# misassemblies	**0**	**0**	**0**	**0**	1	**0**	**0**	**0**	**0**	**0**	**0**	**0**	**0**
# misassembled contigs	**0**	**0**	**0**	**0**	1	**0**	**0**	**0**	**0**	**0**	**0**	**0**	**0**
Misassembled contigs length	**0**	**0**	**0**	**0**	670	**0**	**0**	**0**	**0**	**0**	**0**	**0**	**0**
# local misassemblies	**0**	**0**	1	1	1	1	**0**	1	**0**	1	2	**0**	**0**
# unaligned mis. contigs	0	0	0	0	0	0	0	0	0	0	0	0	0
# unaligned contigs	407 + 0 part	**26 + 0 part**	495 + 0 part	444 + 0 part	73 + 0 part	442 + 0 part	393 + 0 part	478 + 0 part	412 + 0 part	437 + 0 part	433 + 0 part	429 + 0 part	432 + 0 part
Unaligned length	241295	**14816**	296562	263972	42456	264277	233261	286542	244102	259091	256233	254867	257078
Genome fraction (%)	2.354	0.309	59.850	53.862	76.212	87.487	34.830	**88.672**	39.390	45.889	85.597	12.748	20.785
Duplication ratio	**1.000**	**1.000**	1.003	1.002	1.003	1.006	1.002	1.004	1.002	1.001	1.004	**1.000**	1.001
# N's per 100 kbp	**0.00**	**0.00**	**0.00**	**0.00**	**0.00**	**0.00**	**0.00**	0.38	**0.00**	**0.00**	**0.00**	**0.00**	**0.00**
# mismatches per 100 kbp	3.22	**0.00**	1.14	0.49	0.40	0.39	**0.00**	1.41	0.67	0.41	0.44	0.30	2.55
# indels per 100 kbp	**0.00**	**0.00**	0.32	0.28	0.20	0.22	0.11	0.21	0.19	0.25	0.27	0.30	0.55
Largest alignment	1249	1010	3784	4501	7416	9719	3745	10815	2583	3552	**12218**	1442	3169
Total aligned length	62087	8154	1581896	1423454	2016200	2319904	919929	**2347650**	1040300	1211588	2265100	336235	548314
NA50	-	-	895	845	1330	1656	678	1834	715	752	1637	528	579
NGA50	-	-	638	555	1054	1611	-	1832	-	-	1575	-	-
NA75	-	-	591	579	846	918	522	985	534	549	939	-	-
NGA75	-	-	-	-	532	882	-	985	-	-	838	-	-
LA50	-	-	698	658	496	468	598	441	631	677	468	417	508
LGA50	-	-	1201	1360	739	484	-	442	-	-	504	-	-
LA75	-	-	1349	1267	978	989	1081	932	1152	1251	975	-	-
LGA75	-	-	-	-	1619	1032	-	933	-	-	1072	-	-

4.4.2 *E. coli str. K-12 substr. DH10B*

Table 31 contains the Quast report after assembling dataset *E. coli str. K-12 substr. DH10B* with Velvet .

Table 31: Assembly quality metrics for *E. coli str. K-12 substr. DH10B*

Assembly	ACE	BayesHammer	BFC	BLESS2	Blue	Fiona	Karect	Lighter	Musket	RACER	SGA-EC	Trowel
# contigs (≥ 0 bp)	509	491	465	468	451	472	458	502	500	448	174910	154264
# contigs (≥ 1000 bp)	90	87	85	84	81	85	80	89	85	82	0	0
# contigs (≥ 5000 bp)	73	72	69	68	69	69	68	73	72	65	0	0
# contigs (≥ 10000 bp)	66	65	63	62	63	63	63	68	65	62	0	0
# contigs (≥ 25000 bp)	53	54	54	52	51	52	54	56	53	52	0	0
# contigs (≥ 50000 bp)	31	29	30	33	31	31	32	30	28	32	0	0
Total length (≥ 0 bp)	4362719	4361014	4362674	4364353	4362388	4361867	4365893	4361911	4360726	4365116	**12078319**	11049421
Total length (≥ 1000 bp)	4324975	4325779	4329137	4330284	4329569	4327404	4332565	4325368	4324544	**4333539**	0	0
Total length (≥ 5000 bp)	4286089	4294343	4295276	4294887	4306420	4289532	**4307659**	4286673	4295327	4293367	0	0
Total length (≥ 10000 bp)	4232622	4236996	4248144	4248584	4259350	4240854	4268850	4246908	4240129	**4269045**	0	0
Total length (≥ 25000 bp)	4018923	4072492	4119676	4097964	4078566	4068100	4120739	4056582	4050242	**4120791**	0	0
Total length (≥ 50000 bp)	3239468	3169522	3232522	**3417084**	3366933	3325363	3348823	3104697	3150091	3404809	0	0
# contigs	97	92	89	89	85	92	85	97	93	87	13	23
Largest contig	299060	269346	269332	325957	326035	**326112**	269392	269442	269320	269372	709	772
Total length	4330157	4329486	4331937	4333660	4332755	4332343	4336380	4331396	4330245	**4337347**	7189	13177
Reference length	4686137	4686137	4686137	4686137	4686137	4686137	4686137	4686137	4686137	4686137	4686137	4686137
GC (%)	50.76	50.75	50.76	50.76	50.75	50.75	50.75	50.75	50.74	50.75	47.38	48.72
Reference GC (%)	50.78	50.78	50.78	50.78	50.78	50.78	50.78	50.78	50.78	50.78	50.78	50.78
N50	82393	86789	86856	85634	86758	86869	85563	82246	88175	**94801**	543	572
NG50	80642	82312	82298	80585	82465	82297	82028	77343	82274	**86919**	-	-
N75	48577	48022	49515	53169	**54670**	53109	53162	47476	45352	54645	523	527
NG75	41209	41209	41209	**48720**	42578	41209	43173	40034	40673	43982	-	-
L50	16	15	15	14	14	14	15	16	14	14	**7**	11
LG50	18	17	17	**16**	**16**	**16**	17	18	17	**16**	-	-
L75	32	31	31	30	29	30	31	33	31	30	**10**	17
LG75	38	37	37	**35**	**35**	36	36	40	37	**35**	-	-
# misassemblies	7	10	2	5	9	4	8	10	7	13	**0**	**0**
# misassembled contigs	5	6	2	5	7	4	6	8	6	9	**0**	**0**
Misassembled contigs length	477123	372804	92189	353248	456378	261461	439383	459105	481833	778996	**0**	**0**
# local misassemblies	64	39	34	30	46	38	31	49	50	42	**0**	**0**
# structural variations	0	0	0	0	0	0	0	0	0	0	0	0
# unaligned mis. contigs	0	0	0	0	0	0	0	0	0	0	0	0
# unaligned contigs	0 + 1 part	**0 + 0 part**	**0 + 0 part**	**0 + 0 part**	0 + 1 part	0 + 1 part	**0 + 0 part**	**0 + 0 part**	**0 + 0 part**	**0 + 0 part**	**0 + 0 part**	**0 + 0 part**
Unaligned length	1677	**0**	**0**	**0**	617	1037	**0**	**0**	**0**	**0**	**0**	**0**
Genome fraction (%)	92.269	92.246	92.323	92.398	92.347	92.360	**92.455**	92.281	92.281	92.420	0.153	0.281
Duplication ratio	1.001	1.002	1.001	1.001	1.001	1.001	1.001	1.002	1.001	1.001	**1.000**	**1.000**
# N's per 100 kbp	113.44	186.37	125.44	105.66	75.17	125.31	85.79	172.60	181.54	132.92	**0.00**	**0.00**
# mismatches per 100 kbp	23.59	46.89	22.40	19.86	21.77	32.12	32.20	33.69	34.20	30.25	**0.00**	**0.00**
# indels per 100 kbp	14.18	17.91	10.72	7.60	9.75	11.51	5.72	19.03	15.98	14.52	**0.00**	**0.00**
Largest alignment	298898	269196	269332	325947	325917	**326112**	269392	269344	269225	269372	709	772
Total aligned length	4323443	4322221	4326571	4329544	4327068	4328251	**4333032**	4323907	4323828	4331580	7189	13177
NA50	82293	85134	86856	82180	85556	85328	85563	82137	**86912**	82221	543	572
NGA50	75911	80601	**82198**	80193	80440	80741	80714	76130	82166	77233	-	-
NA75	44020	43628	48685	**48720**	47405	**48720**	48718	42528	42953	47742	523	527
NGA75	35956	38583	40090	40155	40137	**40443**	40154	33756	38652	39931	-	-
LA50	16	15	15	15	15	15	15	16	15	16	**7**	11
LGA50	19	**17**	**17**	**17**	**17**	**17**	**17**	19	**17**	18	-	-
LA75	34	33	31	31	31	31	32	34	32	33	**10**	17
LGA75	41	39	**37**	**37**	**37**	**37**	38	41	38	39	-	-

4.4.3 *E. coli str. K-12 substr. MG1655*

Table 32 contains the Quast report after assembling dataset *E. coli str. K-12 substr. MG1655* with Velvet .

Table 32: Assembly quality metrics for *E. coli str. K-12 substr. MG1655*

Assembly	Uncorrected	ACE	BayesHammer	BFC	BLESS2	Blue	Fiona	Karect	Lighter	Musket	RACER	SGA-EC	Trowel
# contigs (≥ 0 bp)	339266	626	47416	12544	556	572	605	584	621	637	566	254087	42939
# contigs (≥ 1000 bp)	0	108	0	1543	110	117	112	106	115	111	116	1	300
# contigs (≥ 5000 bp)	0	88	0	11	86	92	90	85	93	89	90	0	1
# contigs (≥ 10000 bp)	0	79	0	0	76	81	80	77	84	78	80	0	0
# contigs (≥ 25000 bp)	0	59	0	0	57	58	58	58	60	59	57	0	0
# contigs (≥ 50000 bp)	0	32	0	0	35	33	33	33	33	33	34	0	0
Total length (≥ 0 bp)	**17463920**	4568487	2369763	4817502	4566297	4565654	4564079	4562902	4566027	4566259	4567503	14472372	5409096
Total length (≥ 1000 bp)	0	4519097	0	2706314	4523572	4522624	4517645	4518802	4519150	4518503	**4524463**	1446	408072
Total length (≥ 5000 bp)	0	**4475899**	0	63787	4472985	4469468	4471178	4475747	4474711	4473760	4467668	0	5985
Total length (≥ 10000 bp)	0	4409903	0	0	4396521	4383548	4392999	**4415938**	4404793	4388487	4391238	0	0
Total length (≥ 25000 bp)	0	4089760	0	0	4089935	4022552	4051345	**4122523**	4002782	4084172	4024529	0	0
Total length (≥ 50000 bp)	0	3112482	0	0	**3283699**	3114815	3112477	3205490	3055689	3171801	3206713	0	0
# contigs	3	121	3	3067	121	128	124	118	125	124	126	67	1980
Largest contig	867	181755	840	7490	174070	179238	**232264**	208709	223529	194669	174310	1446	5985
Total length	2185	4528167	2268	3794237	4531713	4530848	4526320	4527131	4526451	4527527	**4531785**	41034	1530231
Reference length	4639675	4639675	4639675	4639675	4639675	4639675	4639675	4639675	4639675	4639675	4639675	4639675	4639675
GC (%)	44.94	50.73	45.02	50.40	50.72	50.73	50.72	50.73	50.72	50.72	50.73	38.88	47.47
Reference GC (%)	50.79	50.79	50.79	50.79	50.79	50.79	50.79	50.79	50.79	50.79	50.79	50.79	50.79
N50	808	**86050**	808	1418	80449	77816	68741	82565	67426	83207	82551	581	756
NG50	-	82691	-	1187	80449	77816	68741	82565	66935	80447	80476	-	-
N75	808	42208	620	933	**45313**	41989	42494	43670	41230	42252	42938	530	610
NG75	-	41209	-	634	42451	41057	41773	42284	39834	41187	42459	-	-
L50	**2**	20	**2**	863	20	20	20	19	20	19	20	29	716
LG50	-	21	-	1188	20	20	20	19	21	20	21	-	-
L75	**2**	39	3	1688	38	40	40	38	41	39	39	47	1283
LG75	-	41	-	2513	40	42	42	40	43	41	40	-	-
# misassemblies	-	21	-	0	5	13	23	3	13	10	9	0	0
# misassembled contigs	-	12	-	0	5	8	13	2	9	9	6	0	0
Misassembled contigs length	-	1157326	-	0	470209	704224	1125585	390367	938837	851796	543312	0	0
# local misassemblies	-	34	-	0	14	16	15	13	18	9	28	0	0
# unaligned mis. contigs	-	0	-	0	1	0	0	0	1	0	0	0	0
# unaligned contigs	3 + 0 part	0 + 1 part	3 + 0 part	4 + 0 part	**0 + 0 part**	**0 + 0 part**	**0 + 0 part**	**0 + 0 part**	0 + 1 part	**0 + 0 part**	**0 + 0 part**	4 + 0 part	4 + 0 part
Unaligned length	2185	966	2268	2876	**0**	**0**	**0**	**0**	580	**0**	**0**	2849	2885
Genome fraction (%)	-	97.314	-	81.498	97.505	97.416	97.341	97.477	97.294	97.360	97.427	0.823	32.888
Duplication ratio	-	1.003	-	1.003	1.002	1.002	1.002	1.001	1.003	1.002	1.003	1.000	1.001
# N's per 100 kbp	**0.00**	209.33	**0.00**	**0.00**	242.40	249.20	248.83	121.36	311.04	324.42	251.69	**0.00**	**0.00**
# mismatches per 100 kbp	-	28.31	-	13.38	17.00	14.25	27.50	30.23	30.46	38.63	29.07	0.00	16.38
# indels per 100 kbp	-	13.82	-	2.49	9.81	7.90	13.48	7.83	18.59	21.58	14.16	0.00	1.77
Largest alignment	-	165214	-	7490	174070	164078	173882	165316	163894	173710	173960	1446	5985
Total aligned length	-	4516869	-	3790989	4525352	4521152	4517437	4522868	4514747	4519081	4521328	38185	1527204
NA50	-	63339	-	1418	67256	67120	61390	80344	60834	60803	67089	568	755
NGA50	-	63339	-	1187	66500	63276	60805	80344	59372	60803	65967	-	-
NA75	-	39943	-	933	42223	39213	39194	43540	35870	41187	41209	513	609
NGA75	-	35877	-	633	41781	35852	38151	42197	33397	39885	39168	-	-
LA50	-	24	-	863	21	22	25	20	24	22	22	30	716
LGA50	-	24	-	1189	22	23	26	20	25	22	23	-	-
LA75	-	47	-	1688	42	45	48	39	49	44	43	49	1284
LGA75	-	50	-	2514	44	47	50	41	51	46	45	-	-

4.4.4 *S. enterica*

Table 33 contains the Quast report after assembling dataset *S. enterica* with Velvet .

Table 33: Assembly quality metrics for *S. enterica*

Assembly	Uncorrected	ACE	BayesHammer	BFC	BLESS2	Blue	Fiona	Karect	Lighter	Musket	RACER	SGA-EC	Trowel
# contigs (≥ 0 bp)	236914	606	610	702	553	516	784	494	928	679	490	185562	137268
# contigs (≥ 1000 bp)	0	123	125	219	119	89	265	74	337	188	74	0	2
# contigs (≥ 5000 bp)	0	88	80	131	76	62	159	57	170	116	53	0	0
# contigs (≥ 10000 bp)	0	74	69	89	64	54	103	49	110	83	45	0	0
# contigs (≥ 25000 bp)	0	50	49	37	46	41	38	42	35	45	37	0	0
# contigs (≥ 50000 bp)	0	32	31	19	25	30	21	31	19	28	25	0	0
Total length (≥ 0 bp)	**16980980**	4778207	4780385	4785569	4782707	4785804	4780122	4782938	4783429	4783406	4785994	14944644	12032854
Total length (≥ 1000 bp)	0	4725851	4728507	4722859	4734464	4738927	4715446	4740353	4696102	4726461	**4740565**	0	2320
Total length (≥ 5000 bp)	0	4640618	4619869	4486056	4627089	4672336	4442614	**4703375**	4251262	4534404	4691878	0	0
Total length (≥ 10000 bp)	0	4536709	4542588	4173914	4535891	4615276	4042426	**4642891**	3826601	4294979	4634956	0	0
Total length (≥ 25000 bp)	0	4128922	4247177	3356126	4269112	4428501	3068026	**4533152**	2717304	3707912	4513196	0	0
Total length (≥ 50000 bp)	0	3506624	3601272	2725881	3527369	4046246	2475977	**4169579**	2145483	3149161	4084512	0	0
# contigs	9	136	139	246	133	103	290	86	382	207	86	53	208
Largest contig	684	376781	383910	329670	330926	465834	297231	395892	251830	297189	**590715**	977	1164
Total length	4938	4734253	4737412	4741900	4743751	4748024	4733128	4747774	4729466	4739328	**4748477**	31165	125991
Reference length	4888768	4888768	4888768	4888768	4888768	4888768	4888768	4888768	4888768	4888768	4888768	4888768	4888768
GC (%)	51.44	52.15	52.14	52.13	52.13	52.13	52.13	52.13	52.13	52.13	52.12	48.67	50.77
Reference GC (%)	52.09	52.09	52.09	52.09	52.09	52.09	52.09	52.09	52.09	52.09	52.09	52.09	52.09
N50	543	77913	113532	79951	121679	148114	64795	159595	38266	79565	**170051**	571	597
NG50	-	75950	113466	74996	115686	116587	63079	159595	34856	77960	170051	-	-
N75	503	49652	50887	19679	49854	**72911**	15489	72527	12322	28154	72692	533	538
NG75	-	41430	46030	17932	46529	72565	13399	71471	10899	26138	72527	-	-
L50	**5**	16	14	14	12	10	20	10	25	16	8	24	91
LG50	-	17	15	15	13	11	21	10	27	17	8	-	-
L75	**7**	33	31	46	26	23	64	22	85	40	18	38	148
LG75	-	36	33	52	28	25	72	23	95	44	19	-	-
# misassemblies	**0**	17	14	16	20	18	12	19	12	15	21	**0**	**0**
# misassembled contigs	**0**	14	12	13	13	11	10	13	12	13	11	**0**	**0**
Misassembled contigs length	**0**	1661007	1372517	1655245	1842140	2131028	1196243	2286736	975040	1582981	2523514	**0**	**0**
# local misassemblies	**0**	76	75	51	61	53	47	39	32	51	59	**0**	**0**
# unaligned mis. contigs	**0**	**0**	1	**0**	**0**	**0**	**0**	**0**	**0**	**0**	**0**	**0**	**0**
# unaligned contigs	2 + 0 part	5 + 6 part	6 + 7 part	7 + 2 part	5 + 5 part	5 + 5 part	8 + 3 part	5 + 6 part	10 + 4 part	5 + 7 part	4 + 5 part	**0 + 0 part**	2 + 0 part
Unaligned length	1004	59327	61619	60007	59656	60395	60053	59745	58892	59686	61859	**0**	1049
Genome fraction (%)	0.080	95.526	95.561	95.648	95.687	95.799	95.483	**95.843**	95.387	95.622	95.803	0.637	2.553
Duplication ratio	**1.000**	1.001	1.001	1.001	1.001	1.001	1.001	1.001	1.002	1.001	1.001	**1.000**	1.001
# N's per 100 kbp	**0.00**	99.57	110.04	72.86	116.15	59.86	82.42	76.23	53.28	95.82	76.49	**0.00**	**0.00**
# mismatches per 100 kbp	177.94	19.79	19.16	14.20	18.98	19.30	16.82	21.45	14.60	17.11	18.58	22.46	**10.41**
# indels per 100 kbp	**0.00**	5.16	4.99	2.95	3.42	4.44	4.48	4.74	3.07	5.01	4.29	**0.00**	**0.00**
Largest alignment	684	183671	218248	215441	233900	219065	215581	233926	215296	215652	**377102**	977	1164
Total aligned length	3934	4670996	4671740	4678407	4679569	4685321	4669888	**4685535**	4667366	4675991	4684623	31165	124824
NA50	543	74294	84701	57033	106301	100709	44474	113291	35004	69369	**113670**	571	596
NGA50	-	73998	82002	52943	106301	100709	42286	113291	32911	64433	106015	-	-
NA75	502	40494	44347	18740	43571	**67823**	15059	62786	11835	27284	67822	533	536
NGA75	-	37947	40502	17163	40561	60633	13120	60793	10626	24011	62447	-	-
LA50	**5**	20	17	19	16	17	25	15	29	21	12	24	91
LGA50	-	21	18	21	16	17	27	15	31	22	13	-	-
LA75	**7**	40	36	56	34	31	73	29	92	48	26	38	148
LGA75	-	43	39	63	36	33	82	31	103	53	28	-	-

4.4.5 *P. aeruginosa*

Table 34 contains the Quast report after assembling dataset *P. aeruginosa* with Velvet .

Table 34: Assembly quality metrics for *P. aeruginosa*

Assembly	Uncorrected	ACE	BayesHammer	BFC	BLESS2	Blue	Fiona	Karect	Lighter	Musket	RACER	SGA-EC	Trowel
# contigs (≥ 0 bp)	6427	2157	1659	1464	1172	1207	1371	1117	2156	1783	1115	2128	1982
# contigs (≥ 1000 bp)	1632	563	414	419	254	291	368	239	654	506	249	730	638
# contigs (≥ 5000 bp)	112	137	140	148	124	129	136	124	142	141	128	174	154
# contigs (≥ 10000 bp)	36	74	71	76	80	75	74	81	70	70	81	69	69
# contigs (≥ 25000 bp)	13	44	49	43	47	49	48	43	43	43	47	44	34
# contigs (≥ 50000 bp)	4	25	29	23	29	28	29	29	22	26	27	22	23
Total length (≥ 0 bp)	**6346020**	6285231	6268654	6260914	6262363	6263784	6259600	6259177	6272493	6266341	6262912	6267223	6264247
Total length (≥ 1000 bp)	4750667	5952470	6034708	6062139	6116375	6114471	6080305	**6124380**	5908040	6005704	6123214	5934797	5955713
Total length (≥ 5000 bp)	1814873	4976928	5382652	5396892	5807885	5708795	5484607	**5839731**	4694888	5145507	5829866	4634435	4821830
Total length (≥ 10000 bp)	1315345	4541808	4910354	4890158	5487031	5314653	5030382	**5528596**	4208048	4660558	5491925	3914787	4262171
Total length (≥ 25000 bp)	949575	4069767	4608549	4378326	4984773	4933517	4665902	4956980	3749942	4253437	**4985637**	3558546	3731171
Total length (≥ 50000 bp)	637615	3413547	3872046	3689217	4333071	4222801	4022261	**4458330**	2969521	3619810	4248604	2767450	3372642
# contigs	2880	769	537	528	319	361	462	298	901	658	315	952	838
Largest contig	226239	328059	570411	322996	339286	**571909**	321732	571477	510496	539052	390592	359236	353318
Total length	5653892	6098246	6123822	6140602	6161700	6162690	6147408	6164910	6088141	6113380	**6168877**	6093952	6100522
Reference length	6264404	6264404	6264404	6264404	6264404	6264404	6264404	6264404	6264404	6264404	6264404	6264404	6264404
GC (%)	66.51	66.57	66.58	66.59	66.58	66.58	66.59	66.58	66.58	66.58	66.57	66.59	66.59
Reference GC (%)	66.56	66.56	66.56	66.56	66.56	66.56	66.56	66.56	66.56	66.56	66.56	66.56	66.56
N50	2619	64599	88629	109138	120313	**127693**	108906	110138	47837	78352	119291	38051	84256
NG50	2265	60742	78726	89930	**119565**	117737	108906	110138	46385	70540	118598	37313	69066
N75	1298	9231	25007	18252	41167	32431	25484	**42712**	5758	11152	38042	5176	6390
NG75	1040	7832	17828	14456	36170	31710	21018	**37686**	4988	9608	36985	4699	5662
L50	404	19	17	15	13	13	17	**11**	24	17	14	29	18
LG50	529	20	18	16	14	14	17	**11**	26	18	15	31	19
L75	1187	78	49	54	36	39	46	**33**	118	63	36	162	111
LG75	1581	92	54	60	38	41	50	**35**	143	74	38	188	131
# misassemblies	**9**	36	25	10	25	34	31	24	25	33	28	25	26
# misassembled contigs	**6**	18	13	9	17	16	20	15	17	23	20	17	16
Misassembled contigs length	**231350**	2447728	1089713	1025513	2079225	1690556	1712040	1562762	1779078	2011805	2230473	1542709	1746394
# local misassemblies	**100**	224	169	121	161	139	135	154	183	187	151	126	150
# unaligned mis. contigs	**0**	**0**	**0**	**0**	**0**	**0**	**0**	**0**	1	**0**	**0**	**0**	**0**
# unaligned contigs	5 + 2 part	2 + 5 part	1 + 3 part	**1 + 2 part**	1 + 3 part	2 + 4 part	1 + 4 part	2 + 3 part	2 + 7 part	2 + 6 part	1 + 6 part	1 + 5 part	1 + 7 part
Unaligned length	12588	14810	10655	**10101**	10880	15012	13650	11686	15543	15307	14447	14247	16755
Genome fraction (%)	89.452	96.792	97.408	97.657	97.964	97.931	97.733	**98.057**	96.676	97.177	98.040	96.808	96.870
Duplication ratio	1.007	1.003	**1.002**	**1.002**	**1.002**	**1.002**	**1.002**	**1.002**	1.003	**1.002**	**1.002**	1.003	1.003
# N's per 100 kbp	**118.04**	244.58	219.83	161.30	192.12	176.45	192.72	197.88	224.50	227.53	192.55	186.97	218.33
# mismatches per 100 kbp	39.63	15.06	21.86	11.38	**11.13**	13.09	16.15	23.75	20.34	17.68	15.42	26.70	34.38
# indels per 100 kbp	**12.03**	17.37	18.63	12.91	13.22	12.68	16.53	15.61	18.92	18.20	14.52	17.36	20.25
Largest alignment	226050	298012	570020	322354	339246	**571467**	321649	571031	395446	478427	311783	288241	352989
Total aligned length	5636890	6074306	6105782	6124234	6144447	6142551	6129358	6146957	6066401	6092744	**6148772**	6074595	6078072
NA50	2594	51100	73325	89839	105318	92668	88992	**110076**	45356	60272	103516	34022	52053
NGA50	2249	46410	72650	89169	92378	90062	88992	103328	40119	58932	**103516**	33437	47995
NA75	1291	8505	17828	16034	32144	29497	19133	31936	5555	10085	**33354**	5109	6104
NGA75	1023	7399	13949	13414	29880	27418	15730	29786	4829	8845	**30147**	4426	5399
LA50	411	27	20	17	16	17	19	**12**	29	20	19	34	25
LGA50	537	29	21	18	17	18	19	**13**	31	21	19	37	26
LA75	1201	99	57	59	44	48	57	**40**	132	78	46	178	129
LGA75	1598	114	63	65	47	51	62	**42**	158	90	48	205	150

4.4.6 ***H. sapiens* Chr. 21**

Table 35 contains the Quast report after assembling dataset *H. sapiens* Chr. 21 with Velvet .

Table 35: Assembly quality metrics for *H. sapiens* Chr. 21

Assembly	Uncorrected	ACE	BayesHammer	BFC	BLESS2	Blue	Fiona	Karect	Lighter	Musket	RACER	SGA-EC	Trowel
# contigs ($\geq$ 0 bp)	58213	58395	61700	57945	57438	57170	57769	57668	57427	57852	58297	57344	57247
# contigs ($\geq$ 1000 bp)	6812	6402	6920	6367	6368	6277	6297	6350	6638	6471	6424	6519	6622
# contigs ($\geq$ 5000 bp)	1664	1772	1615	1817	1811	1835	1821	1824	1704	1756	1804	1775	1730
# contigs ($\geq$ 10000 bp)	404	496	386	477	488	535	533	516	446	471	509	470	463
# contigs ($\geq$ 25000 bp)	11	26	16	31	28	26	32	22	19	25	21	22	20
# contigs ($\geq$ 50000 bp)	0	0	0	0	0	0	0	0	0	0	0	0	0
Total length ($\geq$ 0 bp)	31597631	31965979	31766176	32002529	31938926	**32041901**	32038486	31957612	31672081	31818334	32019235	31818763	31734651
Total length ($\geq$ 1000 bp)	26827458	27507605	26550208	27599231	27535385	**27711114**	27675394	27554984	27105876	27343993	27574676	27341004	27179349
Total length ($\geq$ 5000 bp)	14469620	16175954	13958509	16512649	16457706	**16892313**	16750500	16554128	15163420	15814857	16386813	15851231	15456691
Total length ($\geq$ 10000 bp)	5646166	7235250	5447698	7068887	7173080	7760310	**7764712**	7437243	6381022	6825114	7312274	6776893	6569323
Total length ($\geq$ 25000 bp)	305814	741358	449079	873737	799632	725036	**941610**	607723	524852	700429	569855	610919	568491
Total length ($\geq$ 50000 bp)	0	0	0	0	0	0	0	0	0	0	0	0	0
# contigs	8747	8004	9061	7959	7994	7845	7855	7958	8450	8151	8059	8235	8402
Largest contig	31896	39067	39290	34106	44280	32458	**46072**	34407	32368	34493	38599	33802	38545
Total length	28232361	28675142	28101089	28756929	28718924	**28852292**	28810553	28726889	28425853	28563707	28760555	28590292	28479294
Reference length	46709983	46709983	46709983	46709983	46709983	46709983	46709983	46709983	46709983	46709983	46709983	46709983	46709983
GC (%)	39.53	39.66	39.26	39.69	39.68	39.74	39.72	39.73	39.62	39.64	39.73	39.65	39.58
Reference GC (%)	42.22	42.22	42.22	42.22	42.22	42.22	42.22	42.22	42.22	42.22	42.22	42.22	42.22
N50	5159	5885	4963	5993	6024	**6163**	6125	5985	5395	5688	5921	5646	5524
NG50	2054	2425	1914	2472	2440	**2543**	2519	2456	2194	2342	2435	2324	2217
N75	2676	3007	2563	3031	3024	**3099**	3088	3035	2812	2938	2992	2911	2815
L50	1595	1434	1634	1429	1430	**1393**	1397	1424	1522	1469	1437	1481	1499
LG50	4437	3835	4640	3772	3790	**3647**	3685	3775	4176	3953	3808	3980	4114
L75	3512	3153	3616	3122	3122	**3037**	3061	3113	3360	3219	3148	3246	3319
# misassemblies	612	620	**545**	610	732	715	653	573	668	650	644	663	682
# misassembled contigs	467	449	**403**	449	526	502	484	431	478	449	481	515	482
Misassembled contigs length	3326586	3566126	**3019786**	3568048	4202668	4253263	4091809	3584831	3642250	3563055	3822684	3936298	3766679
# local misassemblies	1010	1047	**932**	979	1023	1130	1091	1045	1063	1155	1100	1043	964
# structural variations	0	0	0	0	0	0	0	0	0	0	0	0	0
# unaligned mis. contigs	79	**44**	166	**44**	61	51	50	45	59	58	52	49	62
# unaligned contigs	324 + 573 part	173 + 432 part	547 + 925 part	178 + 385 part	168 + 395 part	168 + 435 part	**162 + 396 part**	169 + 380 part	194 + 477 part	195 + 508 part	174 + 413 part	196 + 420 part	245 + 486 part
Unaligned length	1322048	800832	2326663	748706	778800	807098	788398	**726780**	939040	983333	803632	833188	1008440
Genome fraction (%)	56.047	57.891	53.531	58.259	58.108	58.288	58.223	**58.336**	57.182	57.292	58.184	57.824	57.083
Duplication ratio	**1.028**	1.031	1.031	1.029	1.029	1.030	1.030	**1.028**	1.029	1.031	1.029	**1.028**	1.030
# N's per 100 kbp	3319.46	3550.95	4309.26	3339.67	3350.99	3317.24	3364.41	3182.06	3335.42	3523.72	**3166.64**	3175.33	3480.50
# mismatches per 100 kbp	231.08	227.19	225.64	222.01	220.04	227.97	221.99	223.26	**217.30**	226.65	235.20	230.24	224.21
# indels per 100 kbp	105.34	96.56	107.85	98.84	**95.34**	100.22	102.30	97.87	98.32	101.21	101.90	104.69	102.08
Largest alignment	29853	31527	31348	31451	31659	31447	29919	31822	31611	31801	31288	**32138**	31294
Total aligned length	26211189	27039009	25050677	27217144	27152844	27236838	27204838	**27244840**	26727737	26769049	27180503	27028047	26686854
NA50	4224	4851	3905	4988	4887	5006	4988	**5025**	4475	4661	4859	4712	4525
NGA50	1367	1756	994	1845	1786	1865	1845	**1871**	1566	1653	1807	1718	1566
NA75	2027	2350	1675	2416	2361	2396	2399	**2436**	2177	2245	2360	2295	2163
LA50	1888	1685	1957	1663	1694	1659	1649	**1647**	1808	1743	1697	1731	1785
LGA50	5584	4720	6417	4591	4699	4556	**4555**	4556	5180	4941	4689	4865	5141
LA75	4284	3814	4658	3746	3816	3743	3730	**3710**	4085	3945	3827	3908	4065

4.4.7 *C. elegans*

Table 36 contains the Quast report after assembling dataset *C. elegans* with Velvet .

Table 36: Assembly quality metrics for *C. elegans*

Assembly	Uncorrected	ACE	BayesHammer	BFC	BLESS2	Blue	Karect	Lighter	RACER	SGA-EC
# contigs (≥ 0 bp)	313458	247789	260292	223399	195321	207345	200640	291328	193008	228459
# contigs (≥ 1000 bp)	21506	25791	25237	26495	26687	26077	25766	23368	25455	26639
# contigs (≥ 5000 bp)	452	562	556	1374	1915	2107	2236	434	2549	1384
# contigs (≥ 10000 bp)	104	46	17	103	190	285	269	73	394	123
# contigs (≥ 25000 bp)	5	1	0	1	7	11	10	5	27	5
# contigs (≥ 50000 bp)	0	0	0	0	0	0	0	0	2	0
Total length (≥ 0 bp)	**100514891**	94677328	95949746	96223212	94442104	97069690	95356117	98710361	95898310	97728152
Total length (≥ 1000 bp)	37905692	48928957	48505197	58787718	64099874	64609003	64449307	41827510	**67692343**	59067924
Total length (≥ 5000 bp)	3832338	3819461	3531390	9485659	13824216	15914879	16622537	3441481	**19988122**	9813852
Total length (≥ 10000 bp)	1501701	613323	208156	1312103	2566271	3941268	3712693	1104325	**5832892**	1651044
Total length (≥ 25000 bp)	144182	26729	0	30830	198141	346721	302882	156627	**902471**	142834
Total length (≥ 50000 bp)	0	0	0	0	0	0	0	0	**124006**	0
# contigs	59201	55137	54557	49896	46077	46120	44909	58648	42732	50765
Largest contig	34936	26729	23739	30830	33772	38086	49047	37692	**70494**	34846
Total length	64279025	69730098	69246748	75436498	77994023	78900340	78118154	66601578	**80035157**	76221496
Reference length	100286070	100286070	100286070	100286070	100286070	100286070	100286070	100286070	100286070	100286070
GC (%)	38.74	38.50	37.36	37.78	37.65	37.84	37.60	38.67	37.64	37.96
Reference GC (%)	35.44	35.44	35.44	35.44	35.44	35.44	35.44	35.44	35.44	35.44
N50	1161	1469	1488	1925	2229	2304	2365	1247	**2625**	1897
NG50	735	966	953	1323	1610	1658	1661	804	**1917**	1329
N75	771	907	903	1078	1222	1221	1247	808	**1356**	1064
NG75	-	-	-	505	596	620	600	-	686	527
L50	16144	14108	13793	11163	9769	9315	9018	15713	**8214**	11284
LG50	35856	27027	26915	18971	15665	14807	14640	32673	**12741**	18887
L75	33287	29329	28847	24365	21616	21165	20519	32435	**18870**	24795
LG75	-	-	-	49455	40994	39514	39613	-	34527	48803
# misassemblies	**3096**	6019	5842	6981	8317	7594	8174	4317	8798	6525
# misassembled contigs	**2571**	4736	4640	5488	6301	5792	6221	3504	6669	5157
Misassembled contigs length	**3102213**	8112983	8555204	12333155	16017169	15172010	16532475	4808532	19081800	11271909
# local misassemblies	**507**	922	809	1241	1705	1479	1544	718	1429	1153
# unaligned mis. contigs	93	87	**47**	65	92	77	67	87	87	71
# unaligned contigs	5589 + 220 part	6249 + 393 part	6479 + 341 part	5193 + 420 part	4282 + 567 part	4503 + 532 part	4249 + 480 part	6133 + 347 part	**3909 + 565 part**	5144 + 398 part
Unaligned length	11242801	10009213	**7771997**	9601446	8858941	10229836	9092027	11050356	9511857	10527959
Genome fraction (%)	52.408	58.937	60.701	64.935	68.154	67.705	68.038	54.835	**69.539**	64.807
Duplication ratio	**1.009**	1.010	1.010	1.011	1.012	1.011	1.012	1.010	1.011	1.011
# N's per 100 kbp	**228.09**	487.99	461.80	435.10	527.78	490.59	484.83	348.43	484.22	434.66
# mismatches per 100 kbp	**17.40**	36.43	31.51	32.18	41.89	35.48	38.08	25.36	73.35	32.31
# indels per 100 kbp	**13.79**	28.27	26.68	29.41	34.82	32.06	33.98	19.61	38.98	28.71
Largest alignment	10782	10826	13458	16311	20974	17720	19637	7927	**21899**	14320
Total aligned length	52796255	59255569	61056499	65312826	68475519	68063868	68398255	55196310	**69843929**	65182944
NA50	865	1100	1207	1386	1589	1570	1639	929	**1755**	1336
NGA50	524	670	724	898	1075	1066	1094	565	**1205**	882
NA75	556	618	679	698	771	732	771	568	**791**	673
LA50	22902	18373	16311	14821	13371	13316	12657	21561	**12008**	15481
LGA50	49744	36229	33004	26009	21931	21611	20964	44967	**18983**	26594
LA75	46161	39575	35559	34141	31114	31843	30160	44628	**29099**	35712

4.4.8 *D. melanogaster*

Table 37 contains the Quast report after assembling dataset *D. melanogaster* with Velvet .

Table 37: Assembly quality metrics for *D. melanogaster*

Assembly	Uncorrected	ACE	BayesHammer	BFC	BLESS2	Blue	Fiona	Karect	Lighter	Musket	RACER	SGA-EC	Trowel
# contigs (≥ 0 bp)	100458	88911	119593	85549	79158	82740	84689	81344	93233	90739	80832	87971	89636
# contigs (≥ 1000 bp)	11374	9011	17268	8526	8241	8091	8111	8240	9556	9147	7913	8601	9091
# contigs (≥ 5000 bp)	5987	5107	6804	4877	4733	4635	4720	4722	5364	5155	4613	4931	5143
# contigs (≥ 10000 bp)	3535	3326	3069	3279	3147	3153	3151	3196	3412	3337	3117	3279	3373
# contigs (≥ 25000 bp)	1026	1250	452	1308	1323	1330	1307	1319	1216	1263	1326	1327	1279
# contigs (≥ 50000 bp)	170	326	41	370	385	430	424	399	289	343	420	352	330
Total length (≥ 0 bp)	**119882706**	118726428	119848583	118515422	117590011	118625990	118503169	117997496	118883364	118809330	118363831	119238372	118992862
Total length (≥ 1000 bp)	110945820	111997301	106901197	111963831	111698533	112321082	112161292	111864739	111467757	111860476	112364368	**112403232**	111949002
Total length (≥ 5000 bp)	97402353	102268382	81335021	102855188	102982775	103667092	103651250	103091852	100907891	101846130	**104026229**	103212029	102074935
Total length (≥ 10000 bp)	79831793	89406719	54899046	91272984	91527739	92966207	92357632	92058152	86780805	88726766	**93238860**	91212888	89264983
Total length (≥ 25000 bp)	40228323	56092233	15731892	59703455	61872079	63667742	62631987	61838260	51841237	55635456	**64062266**	59834606	55761936
Total length (≥ 50000 bp)	11289515	24250126	2493053	27166693	28985837	**32552918**	32110811	30144152	20129253	24524703	32425265	26052606	23142099
# contigs	14234	10680	23134	10165	9704	9648	9635	9757	11551	10876	9352	10330	10889
Largest contig	135981	199070	109411	221622	178719	206598	**310228**	201437	209745	192773	244683	181525	132175
Total length	112964497	113189748	111071275	113126301	112747850	113439099	113252292	112945003	112879620	113089933	113390785	**113629454**	113223579
Reference length	120381546	120381546	120381546	120381546	120381546	120381546	120381546	120381546	120381546	120381546	120381546	120381546	120381546
GC (%)	42.60	42.72	42.58	42.67	42.70	42.66	42.68	42.69	42.69	42.63	42.67	42.62	42.62
Reference GC (%)	42.42	42.42	42.42	42.42	42.42	42.42	42.42	42.42	42.42	42.42	42.42	42.42	42.42
N50	17913	24554	9867	26837	28286	29247	28679	27863	22711	24530	**29535**	26543	24455
NG50	16416	22835	8837	24772	26047	27206	26378	25842	20639	22616	**27373**	24862	22520
N75	8526	11868	4675	12862	13459	13756	13529	13415	10623	11586	**14073**	12604	11792
NG75	6914	9612	3561	10392	10598	11144	10940	10722	8757	9402	**11526**	10375	9573
L50	1795	1271	3134	1187	1117	1074	1083	1116	1409	1300	**1055**	1210	1314
LG50	2012	1422	3632	1328	1258	1197	1213	1255	1583	1455	**1179**	1342	1466
L75	4064	2910	7212	2710	2550	2481	2514	2562	3206	2974	**2430**	2744	2972
LG75	4786	3416	8915	3182	3027	2900	2953	3025	3787	3498	**2841**	3188	3478
# misassemblies	2610	2678	**2245**	2943	3004	2763	2805	2964	2837	2865	3015	2763	2752
# misassembled contigs	1702	**1591**	1642	1744	1767	1618	1613	1697	1758	1716	1708	1625	1652
Misassembled contigs length	37525466	45699234	**22889624**	50047465	51430351	51426960	51283937	51124905	44579745	47627338	53793688	48252389	46125881
# local misassemblies	4593	4126	3264	3497	3007	3631	**3003**	3514	3533	5132	3031	4441	4116
# unaligned mis. contigs	140	105	92	81	**68**	91	69	72	113	168	71	87	111
# unaligned contigs	1907 + 1478 part	1626 + 1164 part	2385 + 1185 part	1528 + 931 part	1376 + 859 part	1477 + 937 part	1458 + 809 part	1461 + 835 part	1806 + 1109 part	1672 + 1467 part	**1374 + 807 part**	1632 + 1147 part	1703 + 1267 part
Unaligned length	7337009	6218645	6918913	5229492	4762484	5188181	**4750697**	4835002	6477471	7387838	4789677	6035593	6870105
Genome fraction (%)	86.998	88.216	85.890	89.083	89.225	89.341	89.640	89.211	87.808	87.092	**89.757**	88.695	87.712
Duplication ratio	1.009	1.007	1.007	1.006	**1.005**	1.007	**1.005**	1.007	1.007	1.008	**1.005**	1.008	1.007
# N's per 100 kbp	980.75	835.91	988.73	760.40	831.13	731.83	725.02	754.48	944.90	947.85	**668.91**	817.92	925.11
# mismatches per 100 kbp	564.79	558.01	**555.27**	559.91	559.86	560.81	565.23	560.81	559.92	561.03	571.05	561.40	560.87
# indels per 100 kbp	175.12	171.61	**161.22**	169.16	163.26	167.67	171.56	167.46	173.98	175.49	172.57	171.33	174.66
Largest alignment	111277	**161258**	93672	121105	144032	139852	143266	128118	156286	111130	148210	156346	120063
Total aligned length	104800288	106279684	103443181	107269667	107402520	107600578	107947498	107465148	105741535	104875059	**108061294**	106822163	105640715
NA50	13000	17418	8022	17962	19023	**19579**	19232	18786	15721	16411	19489	18071	16834
NGA50	12003	15974	7179	16511	17330	18078	17860	17351	14419	15066	**18095**	16663	15320
NA75	5801	7874	3504	8262	8529	8942	8896	8589	7186	7128	**8961**	8189	7513
NGA75	4472	6214	2430	6613	6684	7203	7226	6872	5628	5584	**7236**	6642	5864
LA50	2444	1828	3807	1758	1647	**1608**	1631	1656	2002	1908	1622	1779	1891
LGA50	2741	2044	4420	1968	1856	**1793**	1824	1862	2251	2140	1808	1974	2114
LA75	5663	4240	8975	4058	3831	**3745**	3784	3867	4646	4488	3750	4095	4392
LGA75	6750	5011	11352	4790	4589	**4393**	4452	4590	5530	5353	4400	4780	5199

5 Ability of EC tools to improve genome assembly

Error correction does not always lead to a more fragmented assembly. Fig. 2 shows a case where Karect corrects sequencing errors around a poorly covered region. In the initial reads there was a true 21-mer missing, and the error correction allowed the assembler to assemble the two sets of reads into a single contig.

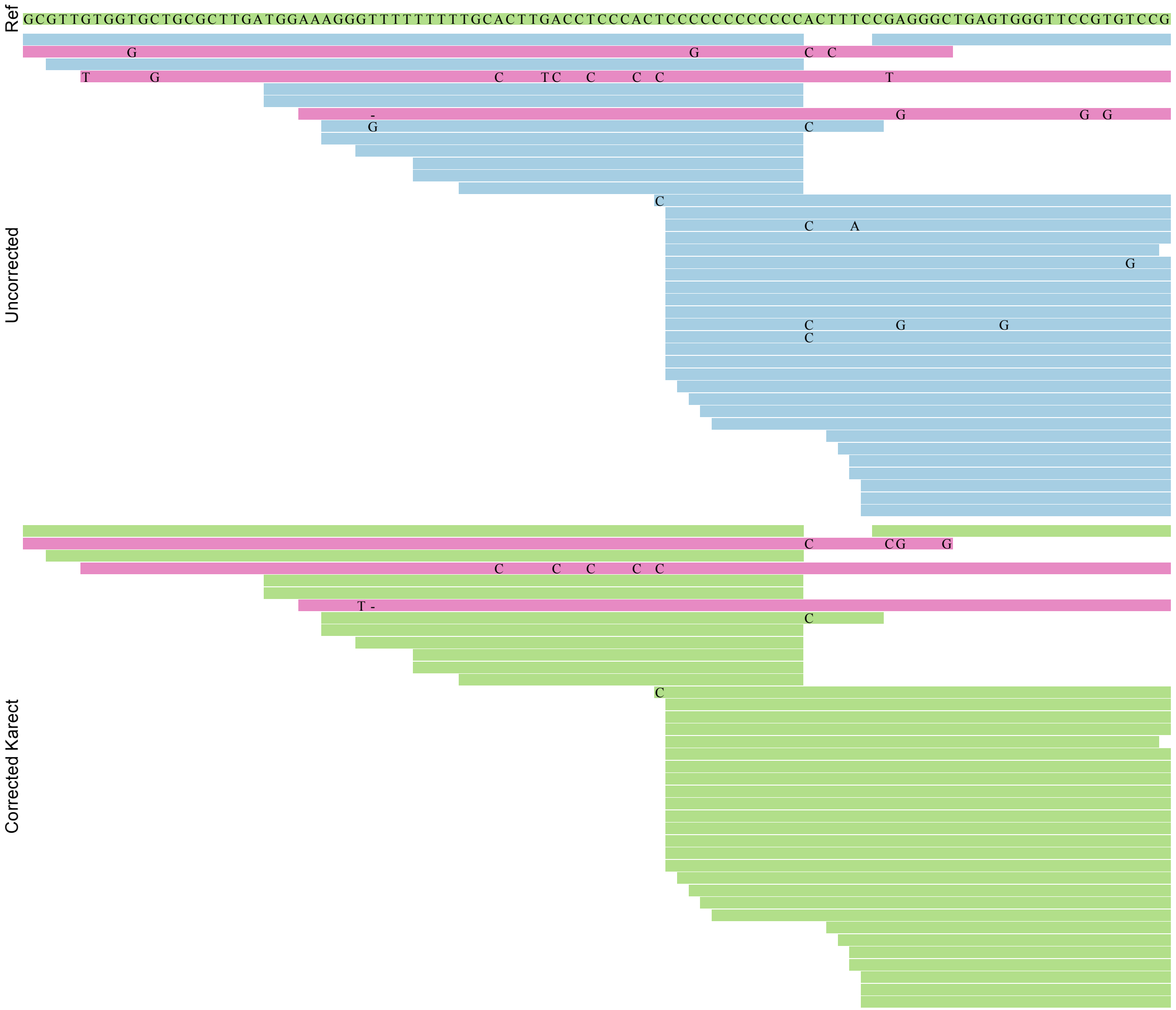

Figure 2: Error correction with Karect resolves a breakpoint in the uncorrected data assembly. The first track (Ref) shows a part of the reference genome, which is assembled into a single contig from Karect-corrected reads. The second track (Uncorrected) shows the alignment of the uncorrected reads to the reference. The third track (Corrected Karect) uses these same alignment positions, but with the sequence content of reads corrected by Karect. The short overlap between the uncorrected reads is less than 21, i.e., a true 21-mer is missing from the uncorrected data. There are three reads which expand along this region but they contain some errors which are highlighted in purple. After error correction those three reads are partially cleaned which suffices to connect the two groups of reads.

6 Memory and Runtime

6.1 Real Data

6.1.1 Memory

In the main paper peak memory usage was measured for all EC tools. Table 38 shows the exact numbers for reference. Furthermore, the possible effects of error correction on the memory usage of assemblers have also been measured. Results are shown for DISCOVAR and SPAdes. Fig. 3 shows the peak memory usage of the EC tools next to the the peak memory usage of SPAdes. While Fig. 4 shows the peak memory usage of the EC tools next to the the peak memory usage of DISCOVAR.

Table 38: Memory usage of EC tools (GB)

Tool	*B. dentium*	*E. coli DH10B*	*E. coli MG1655*	*S. enterica*	*P. aeruginosa*	*H. sapiens* Chr. 21	*C. elegans*	*D. melanogaster*
BayesHammer	9.97	18.87	20.68	15.06	18.48	17.00	21.78	27.38
BFC	0.95	1.06	1.47	0.96	1.18	2.43	5.16	5.22
BLESS2	3.88	3.90	3.89	2.95	3.90	3.90	3.90	3.90
Blue	1.43	2.15	2.42	1.41	1.30	3.66	8.12	8.48
Fiona	6.52	14.08	20.49	3.35	7.11	10.03	43.61	48.06
Karect	22.76	43.98	67.36	10.01	23.62	29.49	135.56	144.18
Lighter	0.35	0.36	0.36	0.36	0.37	0.55	0.82	0.90
Musket	0.78	0.66	0.98	0.74	0.51	0.76	2.40	3.04
SGA-EC	0.27	0.51	0.97	0.19	0.34	0.54	1.95	2.17
Trowel	0.49	0.57	0.90	0.44	0.50	0.52	1.79	2.14

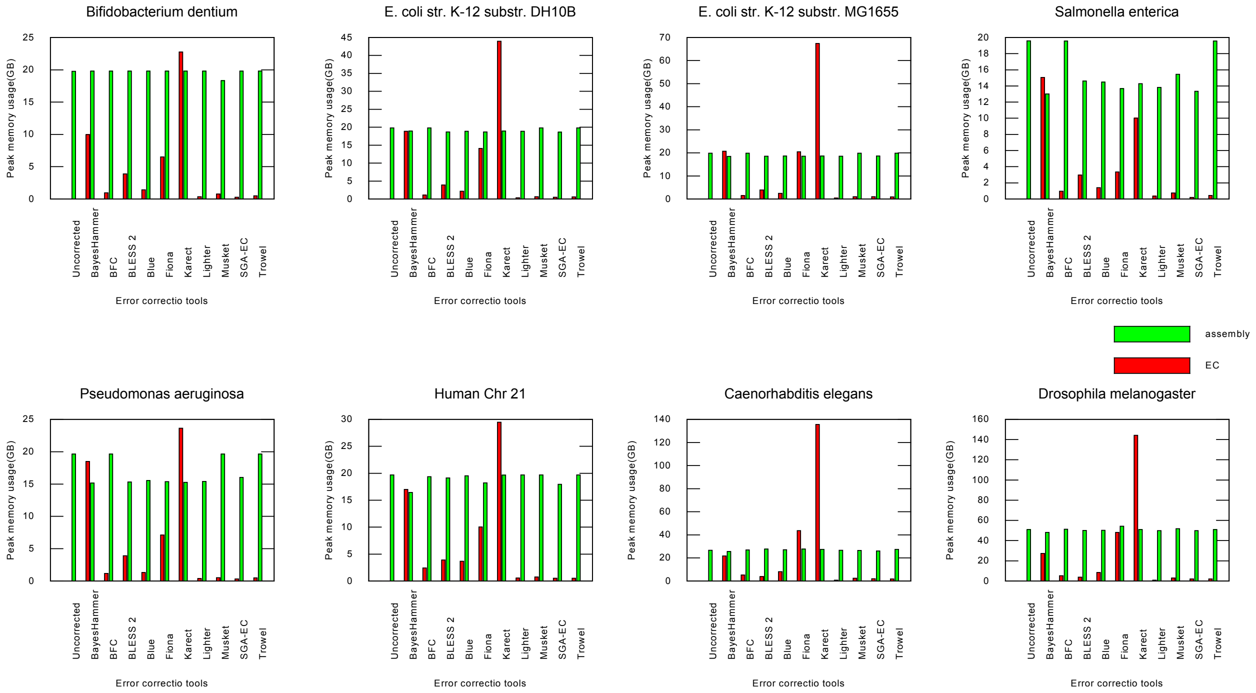

Figure 3: Peak memory usage of SPAdes and EC tools.

6.1.2 Runtime

The runtime plot of the EC tools is shown in the paper, Table 39 shows the exact numbers.

We also recorded the possible effects of error correction on the assembly runtime for DISCOVAR and SPAdes. Fig. 5 shows the total runtime for error correction plus SPAdes assembly. Fig. 6 shows the same graph for DISCOVAR.

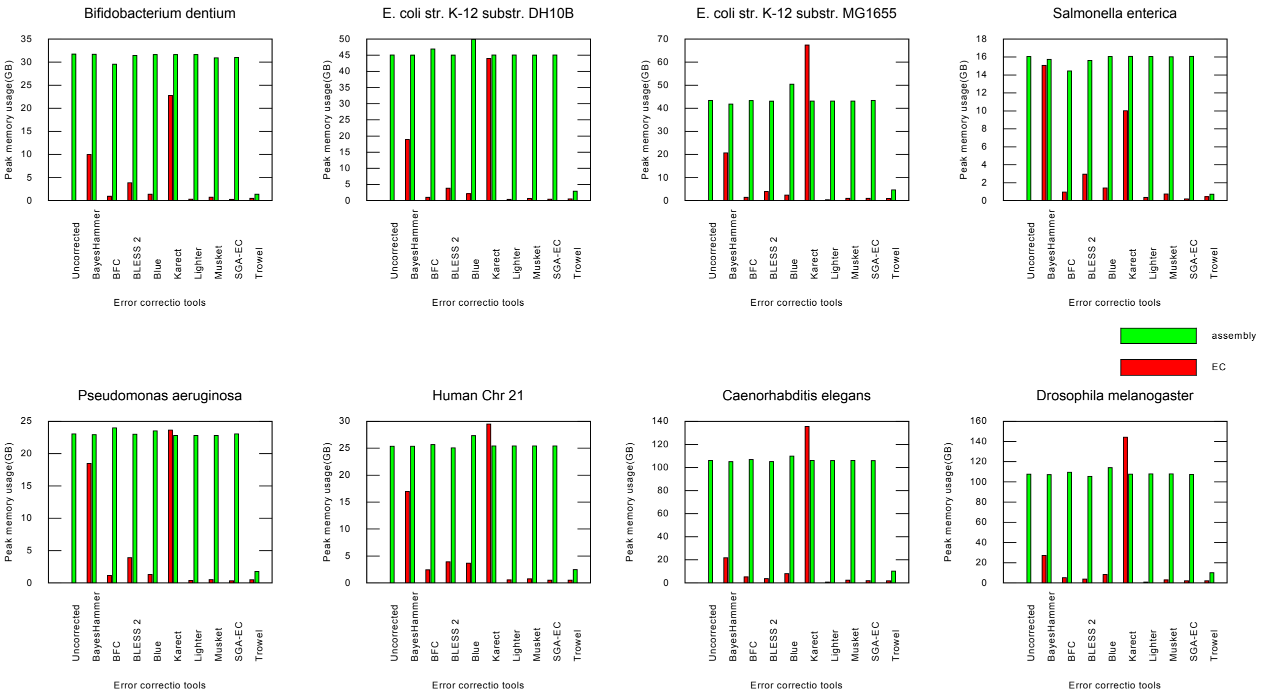

Figure 4: Peak memory usage of DISCOVAR and EC tools.

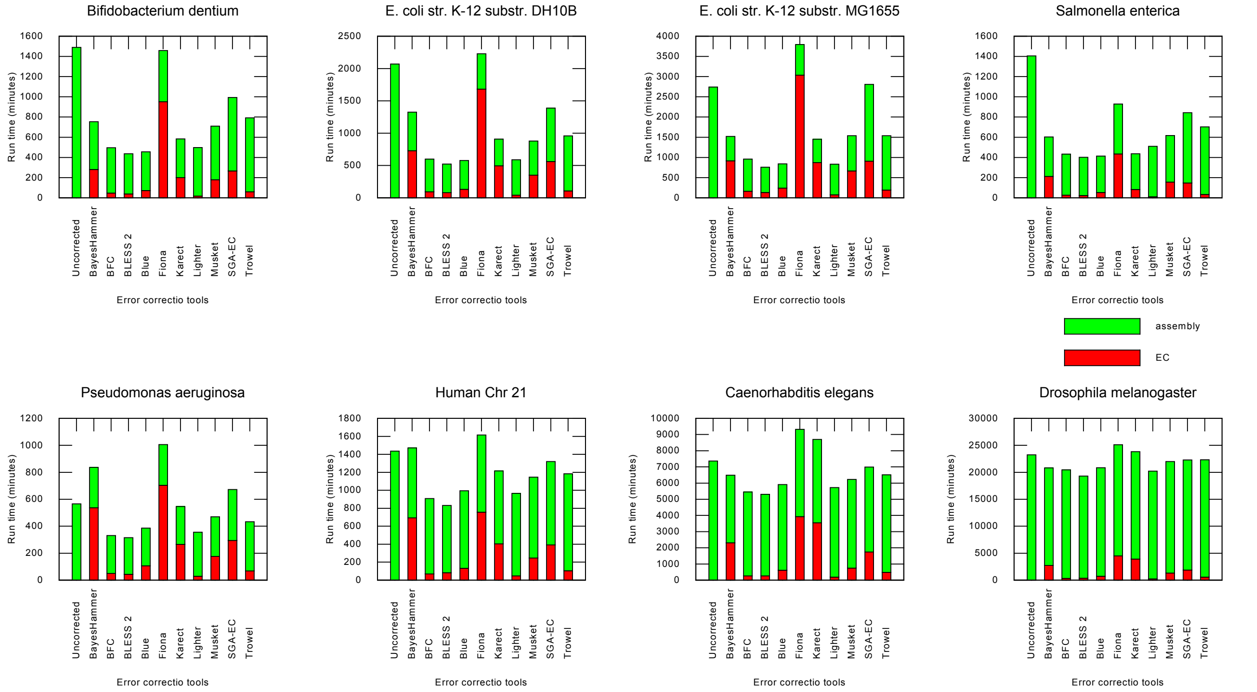

Figure 5: Runtime of SPAdes plus EC tools.

Table 39: Runtime of EC tools (min)

Tool	*B. dentium*	*E. coli DH10B*	*E. coli MG1655*	*S. enterica*	*P. aeruginosa*	*H. sapiens* Chr. 21	*C. elegans*	*D. melanogaster*
BayesHammer	4.66	12.15	15.30	3.54	8.94	11.55	38.50	45.64
BFC	0.78	1.55	2.67	0.43	0.84	1.17	4.44	5.54
BLESS2	0.64	1.31	2.17	0.37	0.73	1.38	4.44	6.02
Blue	1.18	2.20	4.00	0.87	1.78	2.21	10.27	12.26
Fiona	15.87	28.01	50.63	7.24	11.73	12.60	65.50	74.72
Karect	3.33	8.26	14.54	1.39	4.43	6.72	59.08	65.13
Lighter	0.28	0.70	1.26	0.17	0.49	0.78	3.01	3.87
Musket	2.99	5.83	11.10	2.61	2.93	4.11	12.36	21.94
SGA-EC	4.44	9.34	15.13	2.45	4.91	6.52	28.96	31.21
Trowel	1.00	1.79	3.21	0.54	1.13	1.74	7.98	9.13

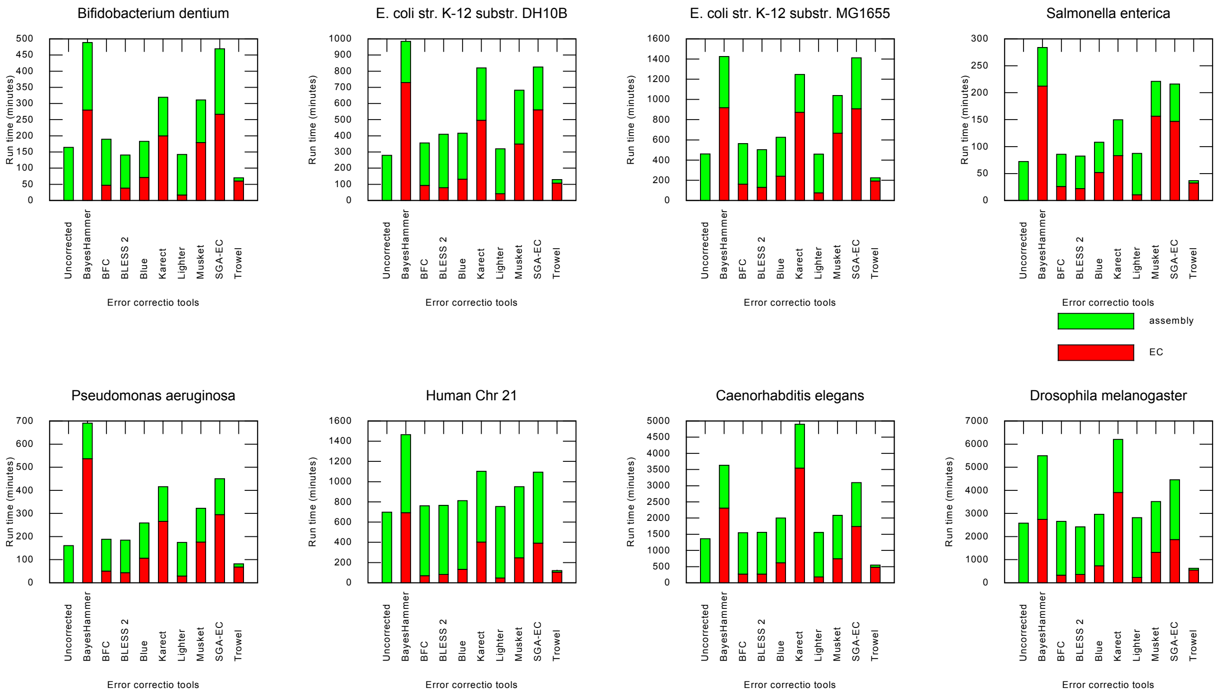

Figure 6: Runtime of DISCOVAR plus EC tools.

6.2 Simulated Data

6.2.1 Memory

Table 40 shows the peak memory usage for all tools in simulated data.

Table 40: Peak memory usage of EC tools.

Tool	*B. dentium*	*E. coli DH10B*	*E. coli MG1655*	*S. enterica*	*P. aeruginosa*	*H. sapiens* Chr. 21	*C. elegans*	*D. melanogaster*
BFC	1.65	1.21	3.40	1.07	1.24	3.00	5.20	7.35
BLESS 2	3.90	3.90	3.91	2.95	3.90	3.89	3.90	3.90
Fiona	6.82	14.03	22.08	3.26	7.79	10.69	43.50	49.33
Karect	22.74	43.99	67.31	10.05	23.64	29.66	135.89	146.49
Lighter	0.35	0.36	0.36	0.36	0.37	0.56	0.81	0.90
Musket	1.05	0.62	1.77	0.76	0.49	1.10	1.58	7.75
SGA-EC	0.32	0.51	1.02	0.21	0.35	0.56	1.86	2.53
Trowel	0.49	0.75	0.88	0.46	0.49	0.51	1.70	1.85

6.2.2 Runtime

We recorded elapsed (wall clock) time to measure the runtime. Table 41 shows the runtime of EC tools on simulated data.

Table 41: Runtime of the EC tools.

Tool	*B. dentium*	*E. coli DH10B*	*E. coli MG1655*	*S. enterica*	*P. aeruginosa*	*H. sapiens* Chr. 21	*C. elegans*	*D. melanogaster*
BFC	0.92	1.74	2.78	0.44	0.87	1.47	4.99	6.75
BLESS 2	0.80	1.24	2.27	0.37	0.71	1.37	4.21	5.57
Fiona	27.58	22.82	53.40	6.84	11.10	12.49	64.93	70.48
Karect	3.29	7.36	16.10	1.30	3.22	9.54	27.81	41.47
Lighter	0.28	0.73	1.39	0.16	0.46	0.91	3.00	3.47
Musket	4.12	2.19	8.09	1.47	1.32	3.78	7.59	17.29
SGA-EC	5.52	8.61	14.32	2.43	4.62	7.34	27.95	34.69
Trowel	1.06	2.00	3.29	0.55	1.18	2.11	6.67	9.96
